# Quantitative PET imaging and modeling of molecular blood-brain barrier permeability

**DOI:** 10.1038/s41467-025-58356-7

**Published:** 2025-03-30

**Authors:** Kevin J. Chung, Yasser G. Abdelhafez, Benjamin A. Spencer, Terry Jones, Quyen Tran, Lorenzo Nardo, Moon S. Chen, Souvik Sarkar, Valentina Medici, Victoria Lyo, Ramsey D. Badawi, Simon R. Cherry, Guobao Wang

**Affiliations:** 1 Department of Radiology, University of California Davis Health, Sacramento, CA USA; 2 Department of Internal Medicine, University of California Davis Health, Sacramento, CA USA; 3 Division of Gastroenterology and Hepatology, University of California Davis Health, Sacramento, CA USA; 4 Department of Surgery, University of California Davis Health, Sacramento, CA USA; 5 Center for Alimentary and Metabolic Sciences, University of California Davis Health, Sacramento, CA USA; 6 Department of Biomedical Engineering, University of California at Davis, Davis, CA USA

**Keywords:** Biomedical engineering, Neurology

## Abstract

Neuroimaging of blood-brain barrier permeability has been instrumental in identifying its broad involvement in neurological and systemic diseases. However, current methods evaluate the blood-brain barrier mainly as a structural barrier. Here we developed a non-invasive positron emission tomography method in humans to measure the blood-brain barrier permeability of molecular radiotracers that cross the blood-brain barrier through its molecule-specific transport mechanism. Our method uses high-temporal resolution dynamic imaging and kinetic modeling for multiparametric imaging and quantification of the blood-brain barrier permeability-surface area product of molecular radiotracers. We show, in humans, our method can resolve blood-brain barrier permeability across three radiotracers and demonstrate its utility in studying brain aging and brain-body interactions in metabolic dysfunction-associated steatotic liver inflammation. Our method opens new directions to effectively study the molecular permeability of the human blood-brain barrier in vivo using the large catalogue of available molecular positron emission tomography tracers.

## Abstract (web summary)

Chung et al. show that total-body dynamic positron emission tomography can allow non-invasive quantitative imaging of the molecular permeability of the human blood-brain barrier using various molecular radiotracers.

## Introduction

**Fig. 1:**
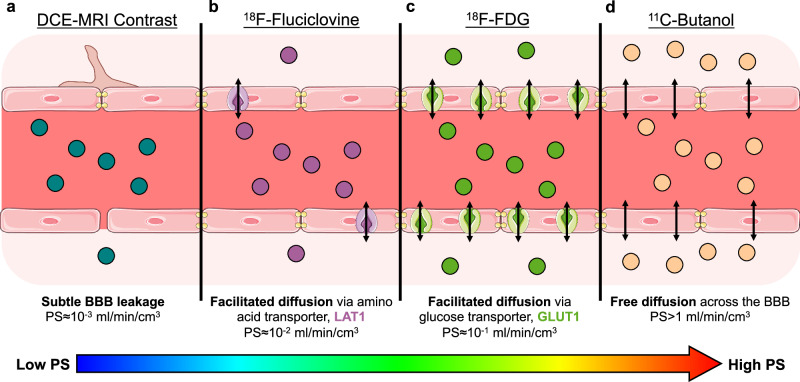
The blood-brain barrier (BBB) is commonly treated as a structural barrier (e.g., tightly lined endothelial cells, tight junction proteins, astrocyte end feet) but also comprises various transport systems and mechanisms associated with molecular BBB permeability^1–3^ that can be imaged using the proposed method with many available positron emission tomography (PET) radiotracers. **a** Current methods of BBB permeability imaging mainly evaluate its structural integrity with gadolinium contrast-enhanced dynamic magnetic resonance imaging (DCE-MRI), where an increase in DCE-MRI measures of permeability reflect non-specific BBB leakage. The BBB permeability-surface area (PS) product with gadolinium DCE-MRI is on the order of 10^−3^ ml/min/cm^3^^8^. **b** ^18^F-fluciclovine is a radiolabeled analogue of an essential amino acid thought to cross the BBB via facilitated diffusion through the large neutral amino acid transporter 1 (LAT1; purple transporter)^71^, which we show has a BBB PS on the order of 10^−2^ ml/min/cm^3^. **c** ^18^F-fluorodeoxyglucose (FDG) is the ubiquitous glucose analogue that mainly crosses the BBB via facilitated diffusion through glucose transporter 1 (GLUT1; green transporter)^72^ and a BBB PS on the order of 10^−1^ ml/min/cm^3^. **d** ^11^C-butanol is a lipophilic radiolabeled alcohol that freely diffuses across the BBB^17,18^ with a BBB PS on the order of >1 ml/min/cm^3^. Of note, the non-specific BBB leakage depicted in (**a**) can be present in (**b**–**d**) but can be neglected due to the relative difference in scales of BBB PS.

The blood-brain barrier (BBB) regulates molecular exchange between the blood and the brain. The BBB not only comprises a structural barrier that tightly restricts blood-to-brain solute diffusion, but also numerous molecular transport systems that support nutritive transport for brain function (Fig. 1)^1–3^. BBB dysfunction is accordingly often associated with a change in BBB permeability, for example, through loss of blood solute filtration during BBB breakdown or through altered BBB transport systems^1–3^.

Neuroimaging of BBB permeability has been instrumental in identifying BBB dysfunction as a hallmark of many neurological and systemic disorders^1,4^. However, current in vivo methods mainly focus on assessing the BBB as a structural barrier. Dynamic contrast-enhanced (DCE) magnetic resonance imaging (MRI) uses gadolinium contrast agents to assess the structural integrity of the BBB under the assumption that these agents do not effectively cross a normal BBB and accordingly have very low normal BBB permeability^5^. The permeability-surface area (PS) product is a specific kinetic measure of BBB permeability^6,7^ with an order of magnitude of 10^−3^ ml/min/cm^3^ for gadolinium MRI contrast agents^8^. Despite certain technical challenges (e.g., signal drift and low signal-to-noise ratio), DCE-MRI measures of BBB permeability have been shown to increase with aging^8^, cognitive impairment^9^, and Alzheimer’s disease due to subtle BBB leakage^10^.

BBB transport also occurs through molecular transporter mechanisms but measuring the associated permeability remains less explored in humans in vivo^1–3^. We hypothesize that measuring the BBB PS of PET molecular radiotracers may open new opportunities to probe the human BBB at the molecular level and advance our basic understanding of BBB physiology. There are numerous PET radiotracers^11^, each with distinct molecular BBB permeability properties stemming from their individual BBB transport mechanisms. For example, the ubiquitous glucose metabolism radiotracer ^18^F-fluorodeoxyglucose (FDG) crosses the BBB mainly via glucose transporter 1 (GLUT1) with a PS on the order of 10^−1^ ml/min/cm^3^^12,13^. Thus, ^18^F-FDG PET has the potential to assess both cellular metabolism and molecular BBB function, with applications in Alzheimer’s disease to study glucose permeability and metabolism^1,14^. The use of other PET radiotracers may allow us to study the BBB permeability of that specific molecule in addition to its molecular target for multiparametric imaging.

However, the BBB permeability of radiotracers has received limited attention in part due to the lack of efficient imaging tools. Currently, two PET scans with two radiotracers are required to measure the BBB permeability of a PET tracer^15–17^, one for measuring BBB transport rate of the target tracer and the other for measuring cerebral blood flow (CBF) using a highly-extracted flow radiotracer (e.g., ^15^O-water^13,17–25^, ^11^C-butanol^17,18,26^, or ^15^O-butanol^20,27^). This approach has faced limited use because dual-tracer protocols are costly, demand extensive infrastructure, and are challenging to execute in part due to the short half-lives of many flow radiotracers. Furthermore, conventional PET scanners have short axial coverage and limited spatial resolution to non-invasively obtain an accurate arterial input function for tracer kinetic analysis^28^, necessitating invasive arterial blood sampling^29^. These factors collectively contributed to the limited exploration of the BBB PS of radiotracers despite the potential ability to probe the molecular permeability of the human BBB in vivo using the large existing catalogue of molecular PET tracers.

Here, we developed a non-invasive multiparametric PET method to image and quantify the molecular BBB PS of radiotracers without a flow tracer PET scan. Our approach is enabled by the recent advent of high-sensitivity long axial field-of-view PET^30–32^ that provides both high-temporal resolution (HTR) dynamic brain imaging^24,32–34^ and arterial blood pool imaging^29,35,36^. Using advanced HTR kinetic modeling, the proposed method jointly estimates CBF and tracer-specific BBB transport rate K_1_ from a single HTR dynamic scan, which in turn provides quantification of the molecular PS of the radiotracer. We tested this method across three very different PET radiotracers and evaluated its application in healthy aging and in patients with metabolic dysfunction-associated steatohepatitis (MASH).

## Results

### High-temporal resolution dynamic PET enables single-scan imaging of CBF and tracer-specific BBB transport rate

**Fig. 2:**
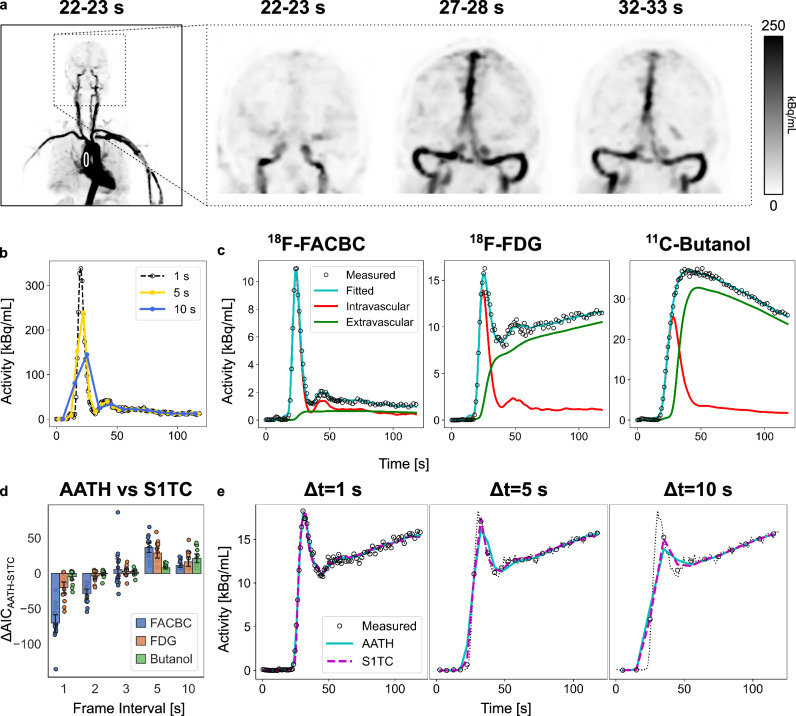
Total-body positron emission tomography (PET)-enabled high-temporal resolution (HTR) dynamic imaging and kinetic modeling for non-invasive quantification of molecular blood-brain barrier (BBB) transport kinetics. **a** Maximum-intensity coronal projections of three 1-s frame dynamic reconstructions (kBq/mL). The extended axial field of view allowed non-invasive measurement of the image-derived input function from the ascending aorta (white outline). **b** A representative image-derived input function illustrating the importance of high temporal resolution to accurately sample the rapid transport of tracer through the blood pool. **c** Representative fits to high temporal resolution time-activity curves of ^18^F-fluciclovine (FACBC), ^18^F-fluorodeoxyglucose (FDG), and ^11^C-butanol using the adiabatic approximation to the tissue-homogeneity (AATH) model. Fitted curves (teal) were decomposed into their intravascular (red) and extravascular tissue (green) distributions according to the AATH model. **d** The difference in Akaike Information Criterion (AIC) between the AATH and the standard one-tissue compartment (S1TC) model time-activity curves frame averaged to different intervals (*N* = 15 samples per tracer from 5 subjects × 3 brain subregions [grey matter, white matter, cerebellum]). Box plot centres indicate average differences and error bars indicate standard deviation of the differences. The AATH model was preferred over the S1TC for 1 to 2 s HTR frame intervals (negative AICs), but not for the 3, 5, 10 s intervals, illustrating the importance of total-body PET in enabling the non-invasive single-tracer BBB PS imaging method. **e** Representative AATH and S1TC fits to an FDG time activity curve (dashed black line: original) in the grey matter at 1, 5, and 10 s frame intervals, with progressively poorer early peak fitting at greater frame intervals.

The ultra-high sensitivity of total-body PET scanners^30–33,37,38^ enables high temporal resolution dynamic brain PET imaging (e.g., 1 to 2 s per frame)^24,32–34^ compared to conventional PET scanners, which are practically limited to 5 to 20 s temporal resolution. Their extended axial field-of-view also allows a fully quantitative whole-blood image-derived input function to be obtained from a major blood pool (e.g., the ascending aorta) while synchronously imaging the brain, obviating the need for invasive arterial blood sampling with minimal delay and dispersion effects^39^ for PET kinetic analysis (Fig. 2a) of radiotracers without significant radiometabolites^29^. The importance of HTR imaging to sample the radiotracer’s rapid first pass in the blood pool with high fidelity (Fig. 2b) is illustrated in ascending aorta data shown at 1 s, 5 s, and 10 s frame durations.

Standard compartmental models assume that a tracer instantaneously crosses and uniformly mixes in local blood vessels, neglecting the finite transit time required for the tracer to traverse the blood volume at a rate equal to blood flow. This assumption is reasonably valid for dynamic PET data at standard temporal resolutions (e.g., 5 to 20 s) but is less suitable for modeling HTR data as indicated by very early studies^18,40,41^ and our initial studies^42,43^. Hence we combined HTR dynamic imaging enabled by total-body PET with advanced kinetic modeling approaches to resolve the tracer’s rapid vascular transit via CBF as well as its extravascular transport. Despite their application in dynamic contrast-enhanced MRI and CT^44,45^, HTR kinetic modeling has historically received little attention in PET^18,40,41^ until recently^24,42,43^ due to the limited count levels of conventional PET scans.

Here, we used the adiabatic approximation to the tissue homogeneity (AATH) model^46^ on the first two minutes of HTR dynamic PET scans (60 × 1 s frames then 30 × 2 s frames) to jointly estimate CBF and tracer-specific BBB transport rate K_1_ from the same model using a single dynamic HTR scan. The AATH model accounts for both intravascular transport and tracer exchange across the BBB. It comprises a total of five model parameters. In addition to CBF and K_1_, the three other parameters are: the BBB clearance rate (k_2_), the mean vascular transit time (T_c_) of tracer travelling through the entire intravascular volume residing in a voxel or region of interest, and the time delay (t_d_) between radiotracer arrival at regional cerebral vessels and the ascending aorta where the image-derived arterial input function was extracted. Additional details are in the Methods.

We tested this method on total-body early dynamic PET scans of fifteen human subjects scanned with either ^18^F-fluciclovine (*N* = 5; mean age 64.4 ± 6.7 y; 5 males, 0 females), ^18^F-FDG (*N* = 5; age 63.6 ± 6.9 y; 5 males, 0 females), or ^11^C-butanol (*N* = 5; age 61.6 ± 6.4 y; 3 males, 2 females) on the uEXPLORER total-body PET/CT system^30^. These radiotracers (Fig. 1) were selected to span a wide range of molecular BBB PS values according to their known ranges of BBB transport rate K_1_ values^12,17,47^. The AATH model accurately fit the measured brain TACs for all investigated radiotracers at HTR (Fig. 2c). By the Akaike Information Criterion (AIC)^48^, the AATH model better fit the measured data at 1 to 2 s HTR, but the standard one-tissue compartment model was favoured at 5 to 10 s temporal resolution (Fig. 2d), highlighting the need for HTR to enable our method.

The average estimated regional CBF across the fifteen included subjects was 0.476 ± 0.100, 0.173 ± 0.036, and 0.427 ± 0.069 ml/min/cm^3^ in the cortical grey matter, white matter, and cerebellum, respectively. These values were within the expected ranges of average regional CBF previously established with flow-tracer PET (grey matter: 0.44 to 0.83 ml/min/cm^3^^17–20,26,27^; white matter: 0.16 to 0.32 ml/min/cm^3^^17–19,26,27^; cerebellum: 0.41 to 0.56 ml/min/cm^3^)^19,21,22^. White-to-grey matter CBF ratios were 0.359 ± 0.048, 0.354 ± 0.069, and 0.395 ± 0.032 for ^18^F-fluciclovine, ^18^F-FDG, and ^11^C-butanol, respectively, in agreement with other flow-tracer brain PET studies^19,20,25^. There were no significant differences (*P* > 0.50) in regional CBF across PET tracers (Supplementary Table 1), suggesting our method could estimate CBF consistently across radiotracers. In contrast, BBB K_1_ was significantly different between radiotracers (*P* < 0.001), as expected, with increasing values from ^18^F-fluciclovine, ^18^F-FDG, to ^11^C-butanol. ^11^C-butanol K_1_ was nearly equal to CBF estimates in all brain regions (Supplementary Table 1). Of note, we follow the unit convention used by the original AATH model and in DCE-MRI literature in which CBF, K_1_, and PS are expressed per unit voxel volume rather than extravascular tissue volume as commonly used in PET. This generally has negligible impact on quantification in the brain due to its small regional blood volume fraction (Supplementary Table 1) but may have greater impact on regions that intersect with larger cerebral vessels.

Estimated mean vascular transit time T_c_ values ranged on average from 4 to 7 s for ^18^F-fluciclovine and ^18^F-FDG with longer vascular transit times in the white matter, agreeing with previous dual-tracer PET estimates^25^. For ^11^C-butanol, our estimates of T_c_ and cerebral blood volume were greater than the expected physiological range (Supplementary Table 1). This may be due to the rapid extravasation and complete BBB extraction of ^11^C-butanol causing the intravascular and extravascular spaces to become indistinguishable. Nonetheless, we consider T_c_ a method parameter and despite an overestimation of T_c_, our estimates of CBF and K_1_ using ^11^C-butanol were within expected physiological ranges and quantitative values.

### Molecular BBB PS differs across PET radiotracers

The joint estimation of CBF and BBB K_1_ enables calculation of the tracer extraction fraction (E; E = K_1_ / CBF) and the BBB PS using

1 $${{\rm{PS}}}=-{{\rm{CBF}}}\cdot {\mathrm{ln}}\left(1-{{\rm{E}}}\right)$$

based on the Renkin-Crone equation^49,50^. Note that existing dual-tracer methods with conventional PET scanners require an additional flow-specific radiotracer to separately estimate CBF to calculate E and PS. As expected, extraction fractions differed significantly between ^18^F-fluciclovine, ^18^F-FDG, and ^11^C-butanol (*P* < 0.001). The median (interquartile range, IQR) E across all brain regions were 4.7% (IQR: 3.7 to 5.1%), 32.6% (IQR: 29.9 to 38.4%), and 100% (IQR: 94.7 to 100%) for ^18^F-fluciclovine, ^18^F-FDG, and ^11^C-butanol, respectively.

**Fig. 3:**
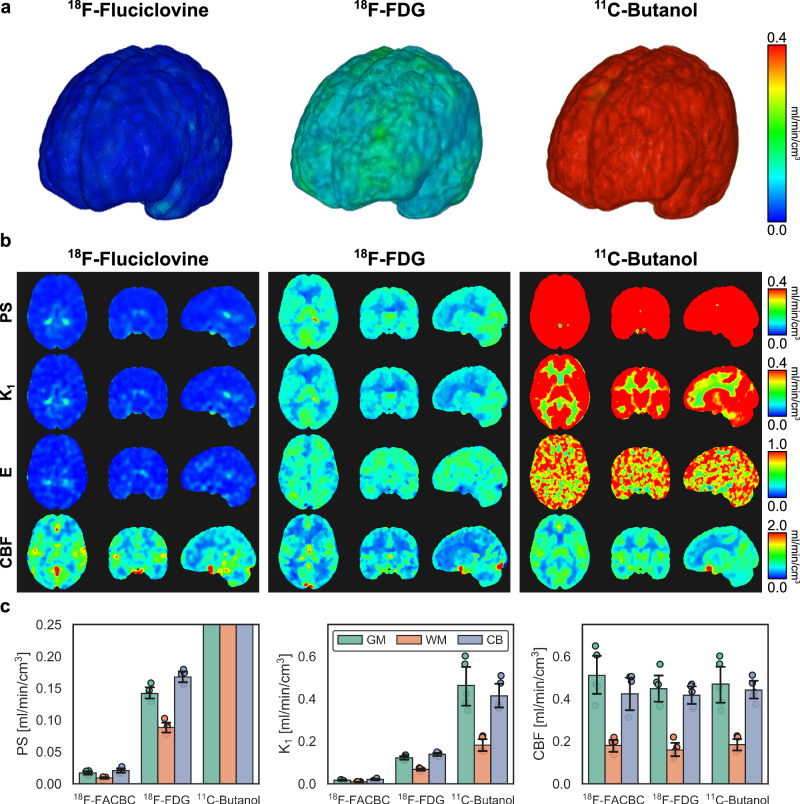
Imaging and quantifying the molecular blood-brain barrier (BBB) permeability-surface area (PS) product of three positron emission tomography (PET) radiotracers. **a** 3D renderings of molecular BBB PS maps with ^18^F-fluciclovine (FACBC), ^18^F-fluorodeoxyglucose (FDG), and ^11^C-butanol illustrating the spectrum of molecular BBB PS across PET radiotracers. **b** Orthogonal slices of tracer PS, BBB transport rate (K_1_), extraction fraction (E), and cerebral blood flow (CBF) for each tracer. Parametric images were aligned to the Montreal Neurological Institute-152 space for visualization. Of note, the hot spots in the ^18^F-fluciclovine PS and K_1_ maps are near the choroid plexus, reflecting the higher inherent permeability of the blood-cerebrospinal fluid (CSF) barrier compared to the BBB, while for the butanol PS map, the cold spots are at CSF pools in the ventricles. **c** Regional quantification at the grey matter (GM), white matter (WM), and cerebellum (CB) shows substantial differences in PS and K_1_ between tracers (averaged across *N* = 5 subjects per tracer; error bars indicate standard deviation of measurement across subjects) while CBF appears comparable between tracers. PS, K_1_ and CBF are in units of ml/min/cm^3^; E is unitless.

We found that BBB PS greatly differed between the three investigated PET tracers (Fig. 3a), with BBB PS on the order of 10^−2^, 10^−1^, and >1 ml/min/cm^3^ for ^18^F-fluciclovine, ^18^F-FDG, and ^11^C-butanol, respectively. The mean ± standard deviation whole-brain PS of ^18^F-fluciclovine and ^18^F-FDG were 0.016 ± 0.003 and 0.132 ± 0.010 ml/min/cm^3^, respectively, while that of ^11^C-butanol was indeterminately high due to its free apparent diffusion across the BBB (i.e., Eq. (1) is indeterminate when E is 1)^17^. The radiotracers with higher apparent BBB PS had greater extravascular distribution according to the area underneath the intravascular and extravascular subcomponents of the fitted curve (Fig. 2c) in part due to their differing BBB permeabilities. Regional BBB PS was significantly different between ^18^F-fluciclovine and ^18^F-FDG (*P* < 0.001) in the grey matter, white matter, and cerebellum. Average regional brain kinetics are summarized in Supplementary Table 1.

Regional differences in BBB PS (Fig. 3b) were observed in parametric imaging, particularly for ^18^F-FDG, which had substantial grey-white matter contrast and elevated PS in the cerebellum, both of which were corroborated by regional kinetic analysis (Fig. 3c). For ^18^F-fluciclovine, BBB PS, K_1_, and E overall had small values with the exception of hot spots seen at the blood-cerebrospinal fluid (CSF) barrier of the choroid plexus, which is known to be inherently permeable in part due to fenestrations at its vasculature^51^. In contrast, ^11^C-butanol had very high PS values and many voxels had E values of 100% (Fig. 3b). The cold spots in the ^11^C-butanol PS map were at ventricular CSF pools leading to lower PS. CBF maps (Fig. 3b) appeared visually comparable between PET tracers, though the prominence of veins (e.g., sagittal sinus) appeared to decrease for radiotracers with higher PS possibly due to their higher tissue extraction leaving less radiotracer concentration cleared through the venous circulation. Of note, E is not always coupled with CBF, as higher ^18^F-fluciclovine E in the choroid plexus did not result in a higher CBF, and conversely, low ^18^F-fluciclovine E in the brain parenchyma did not result in a low CBF.

### AATH model parameters are identifiable across experimental conditions

The AATH model comprises five parameters compared to four parameters in the standard one-tissue compartment model. Note that E and PS are calculated from the AATH model parameters. To characterize the identifiability of AATH model parameters, we conducted practical identifiability analysis^52^ across a wide range of tissue kinetics. Practical identifiability analysis showed that molecular BBB transport kinetics were exceptionally identifiable, with <5% parameter absolute bias and <15% error standard deviation for tissue kinetics spanning our three investigated radiotracers (Supplementary Table 2). Parameter estimation accuracy differed between radiotracers, (e.g., standard deviation of PS and K_1_ estimates were lower for ^18^F-FDG than ^18^F-fluciclovine), but relative errors were small nonetheless. Our estimates of brain kinetics therefore appear reliable across radiotracers.

We then characterized the identifiability of PS, K_1_, E, and CBF for different simulated values of E, T_c_, and CBF (Supplementary Fig. 1). The practical identifiability of PS was generally excellent (absolute mean error <5%, standard deviation <15%) across a wide range of extraction fractions, but deteriorated at very low (E < 2.5%) and high (E > 75%) extraction fractions (Supplementary Fig. 2). At very low values of E, the identifiability of K_1_ and E worsened as only a small proportion of radiotracer is extravasated into the brain; accordingly, the identifiability of PS is poor. Similarly, for high values of E, a small change in extraction fraction can cause a large change in PS (Eq. 1).

The mean error of CBF was generally consistent across extraction fractions, but the standard deviation of the error decreased at higher extraction fractions. However, the identifiability of CBF differed with T_c_ and was worse for smaller values of T_c_. Of note, a negative mean CBF error was observed for T_c_ = 3.0 s because the lower bound of T_c_ estimates was set at 3.0 s (Methods). The PS product—the main parameter of interest in this work—as well as the K_1_, were not strongly affected by changes in T_c_. The identifiability of all parameters improved with greater simulated CBF. Equations (2) and (4) show that CBF is a linear scaling factor of the AATH time-activity curve when E is fixed; accordingly, a greater CBF improves the signal-to-noise ratio of the time-activity curve when other parameters are kept constant. Estimation of PS was also consistent when simulating a fixed PS while manipulating CBF (Supplementary Fig. 2b).

Sensitivity analysis of the AATH model parameters suggested that CBF and K_1_ could be independently estimated (Supplementary Fig. 3 and Supplementary Table 3). Errors in t_d_ and T_c_ estimations appeared strongly correlated with that of CBF and E, but only weakly with PS or K_1_ (Supplementary Fig. 3). Underestimating t_d_ led to an overestimation of PS and K_1_, whereas this led to an underestimation of E and CBF. In contrast, underestimating T_c_ generally led to an underestimation of PS and K_1_ and overestimation for E and CBF, though the trend was less predictable at high extraction fractions. Despite these correlations, our practical identifiability analysis showed that our parameters of interest had low bias (< 5%) and standard deviation (< 15%) across a wide range of kinetics.

The three investigated radiotracers had negligible radiometabolites during the 2-min analysis time frame^26,47,53^ and radiotracers were rapidly injected as a sharp bolus. To understand the effects of nonnegligible radiometabolite fraction and slower injections, we further studied how the characteristics of the arterial input function impacts the identifiability of AATH parameters. First, we conducted practical identifiability analysis using tissue time-activity curves generated with a radiometabolite-corrected plasma input function based on a population-based parent fraction of ^18^F-florbetaben^54^ (Supplementary Fig. 4). Here, approximately 80% of the parent compound remained in plasma at 2 min (Supplementary Fig. 4a). Using the uncorrected plasma input function resulted in a systematic bias of approximately 5% in PS, K_1_, and E, with marginal changes to CBF and the standard deviation of the parameter estimation errors. Despite the relatively small error, in practice, a population-based model may be used to correct the fraction of metabolites for the plasma input function, like many other studies using conventional kinetic modeling. Second, we studied the effect of using a slower injection protocol by simulating three levels of dispersion in the arterial input function (Supplementary Fig. 5). The simulated slower injections had the most impact on the identifiability of CBF, with error mean and standard deviation increasing with greater levels of dispersion. This also affected the standard deviation of E estimates, but otherwise appeared to have a small effect on the identifiability of PS and K_1_.

### BBB PS of ^18^F-FDG decreases in healthy aging

**Fig. 4:**
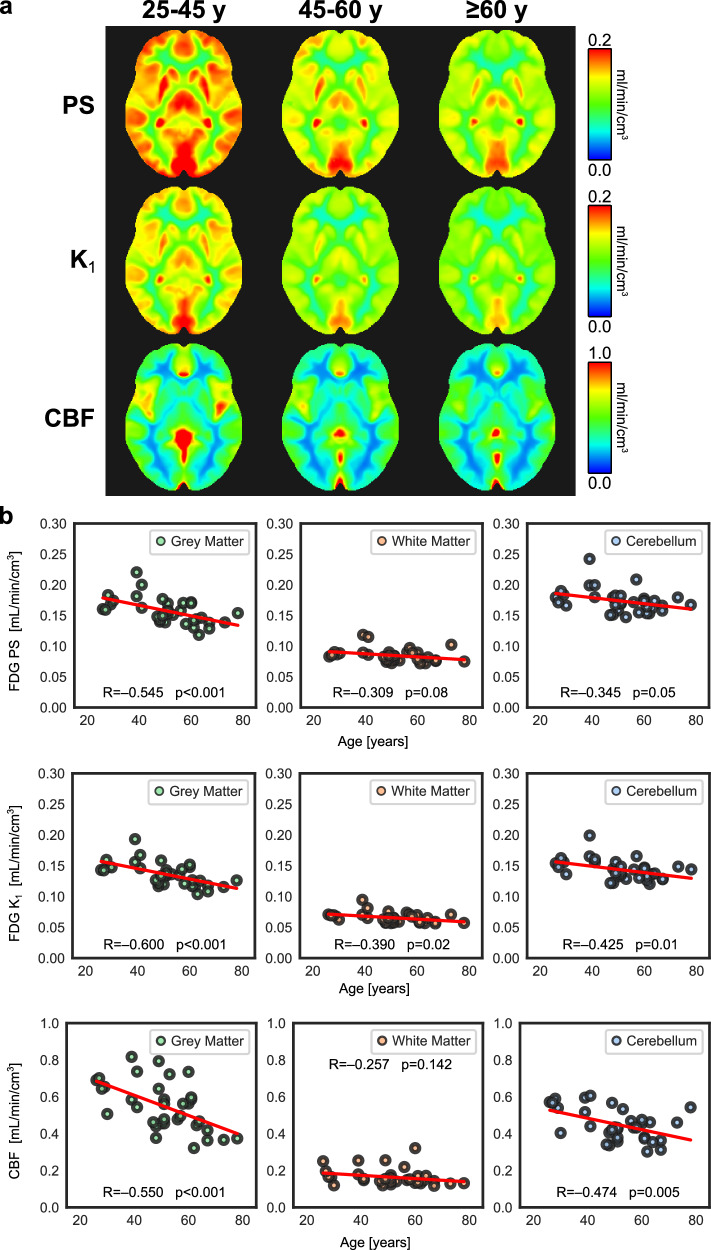
Blood-brain barrier (BBB) permeability-surface area (PS) product, BBB transport rate (K_1_), and cerebral blood flow (CBF) in healthy aging with ^18^F-fluorodeoxyglucose (FDG) positron emission tomography (PET). **a** Parametric images of FDG BBB PS, K_1_, and CBF non-rigidly registered to the Montreal Neurological Institute-152 space and averaged across healthy subjects in three age groups (25–45 y, *N* = 9; 45–60 y, *N* = 14; ≥60 y, *N* = 11). **b** Regional analysis shows significant decreases in FDG BBB PS in grey matter with age, while decreasing trends approached significance in white matter and cerebellum. FDG BBB transport K_1_ significantly decreased in all brain regions likely due to joint decrease of CBF and PS with age. PS, K_1_, and CBF are in units of ml/min/cm^3^. Pearson correlations (R) between regional brain kinetics and age were computed with two-tailed significance testing.

To demonstrate a potential application of the proposed method, we studied the association between age and the BBB permeability of ^18^F-FDG in healthy subjects. BBB breakdown and decreased glucose metabolism have been associated with aging^8,55,56^, but it remains unclear whether BBB permeability changes at the molecular level in aging brains. We analyzed thirty-four healthy subjects in their mid-20s to late-70s (mean age: 51.0 ± 13.3 years; 13 males, 21 females) who underwent total-body dynamic FDG-PET. Regional HTR kinetic analysis of cortical grey matter showed that FDG BBB PS was significantly associated with age (*P* < 0.001) (Fig. 4). Linear regression predicted a cohort decrease in cortical FDG BBB PS of 8.58 × 10^−4^ ml/min/cm^3^ per year of older age, corresponding to a 0.23% decrease in cortical FDG BBB PS per year. A decreasing trend with age was similarly seen for FDG BBB PS in white matter and cerebellum but associations only approached significance (*P* = 0.08 and *P* = 0.05, respectively). Other demographic factors such as sex and body mass index (BMI) were not significantly associated with FDG BBB PS at other brain subregions in our investigation. BBB transport rate K_1_ showed significant associations with age in all studied brain regions (Fig. 4; *P* < 0.05), likely related to the joint decrease of FDG BBB PS and CBF with age (Fig. 4). Linear regression predicted that K_1_ decreased at a rate of 0.27%, 0.15%, and 0.15% per year in the cortical grey matter, white matter, and cerebellum, respectively, in our cohort. Regional CBF and FDG BBB PS were generally correlated in our healthy cohort (Supplementary Fig. 6a) but note that they differ in scale and each represents a distinct physiological feature.

To visualize inter-subject parametric averages across demographics, we non-rigidly transformed each subject’s brain parametric images into the Montreal Neurological Institute (MNI)-152 space^57,58^ and averaged across three age ranges (25–45 years, *N* = 9; 45–60 years, *N* = 14; ≥60 years, *N* = 11). Inter-subject average parametric images showed progressively decreasing FDG BBB PS and K_1_ with age, particularly in the grey matter. Similar decreases in CBF were observed with age (Fig. 4) as expected from prior work^59^, but no significant associations were detected for FDG extraction fraction. Our non-invasive single-tracer method resolved multiparametric associations with age and may have utility in studies of the aging human BBB.

### Evaluating brain-body crosstalk in metabolic dysfunction-associated steatohepatitis

We leveraged total-body dynamic PET and our ^18^F-FDG BBB permeability imaging method to explore brain-body crosstalk in systemic disease states. We studied metabolic dysfunction-associated steatotic liver disease (MASLD), the most common chronic liver disease globally^60^ with potential associations with cognitive impairment^61^. However, there is a paucity of data on the involvement of the BBB in MASLD-related cognitive impairment in humans, especially at the molecular level. Here, we conducted a human BBB study in MASLD with total-body dynamic FDG-PET, applying our FDG BBB PS method in thirty patients with biopsy-graded MASLD-related liver inflammation (i.e., MASH)^62^ and compared against thirteen age-matched healthy controls.

**Fig. 5:**
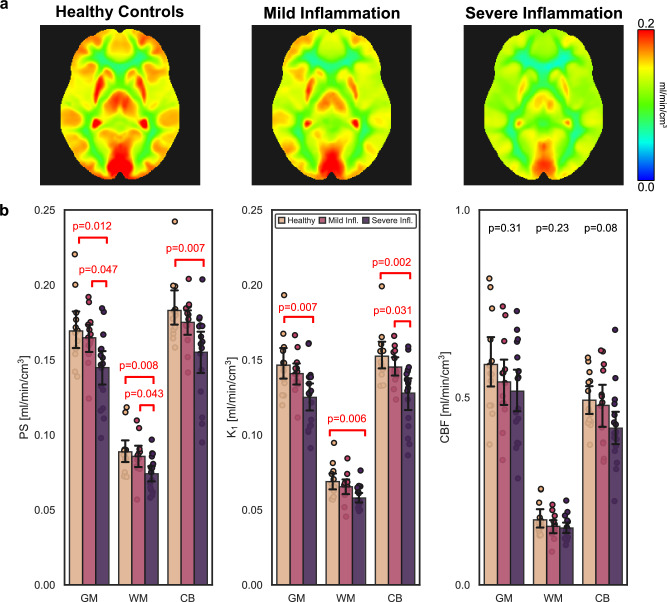
Blood-brain barrier (BBB) permeability-surface area (PS) product of ^18^F-fluorodeoxyglucose (FDG) in metabolic dysfunction-associated steatotic liver disease (MASLD). **a** FDG BBB PS parametric images non-rigidly registered to the Montreal Neurological Institute-152 space and averaged across subjects grouped as age-matched controls (*N* = 13) and patients with mild (*N* = 13) and severe (*N* = 17) MASLD-related lobular liver inflammation. The average FDG BBB PS of patients with severe lobular inflammation was significantly lower than that of mild inflammation and controls. **b** Regional analysis of FDG BBB PS, FDG BBB transport rate (K_1_), and cerebral blood flow (CBF) in the same subjects (*N* = 13 for controls, *N* = 13 for mild inflammation, *N* = 17 for severe inflammation; box plot centres indicate averages, error bars indicate standard deviation) also supported significant decreases in FDG K_1_ mainly between controls and severe inflammation, but no significant differences were observed with CBF. GM indicates grey matter, WM white matter, CB cerebellum. Two-tailed one-way analysis of variance with post hoc Bonferroni-corrected pairwise comparisons were used for statistical analysis of regional kinetics.

Parametric imaging of FDG BBB PS and regional analysis showed decreased FDG BBB PS in patients with severe hepatic lobular inflammation (*N* = 17; mean age 51.0 ± 11.0 y; 3 males, 14 females) compared to those with mild inflammation (*N* = 13; age 52.4 ± 13.0 y; 5 males, 8 females) and age-matched controls (*N* = 13; age 49.6 ± 12.5 y; 3 males, 10 females; Fig. 5). Mean grey matter FDG PS was 0.145 ± 0.025 ml/min/cm^3^ in the severe inflammation cohort, which was significantly lower than that of mild inflammation (0.165 ± 0.017 ml/min/cm^3^; *P* = 0.047) and age-matched controls (0.169 ± 0.022 ml/min/cm^3^; *P* = 0.013). Significant differences mostly persisted in white matter and cerebellum. Similarly, FDG BBB K_1_ significantly differed (*P* < 0.01) between healthy controls and severe liver lobular inflammation groups in all brain regions of interest, but not between mild and severe inflammation groups except in the cerebellum (*P* = 0.031). CBF did not significantly differ (*P* > 0.05) between the three groups in any brain regions of interest, suggesting an effect on the BBB but perhaps not CBF in our cohort of MASLD patients with severe liver inflammation. Accordingly, CBF and FDG BBB PS were not strongly correlated in this cohort at the regional level in contrast to our analysis of healthy volunteers (Supplementary Fig. 6). Taken together, severe liver inflammation may be a contributing factor to MASLD-related BBB dysregulation, possibly through proinflammatory cytokines circulating in blood and disrupting BBB transport^4^.

### BBB PS of ^18^F-FDG is associated with fasting blood glucose

**Fig. 6:**
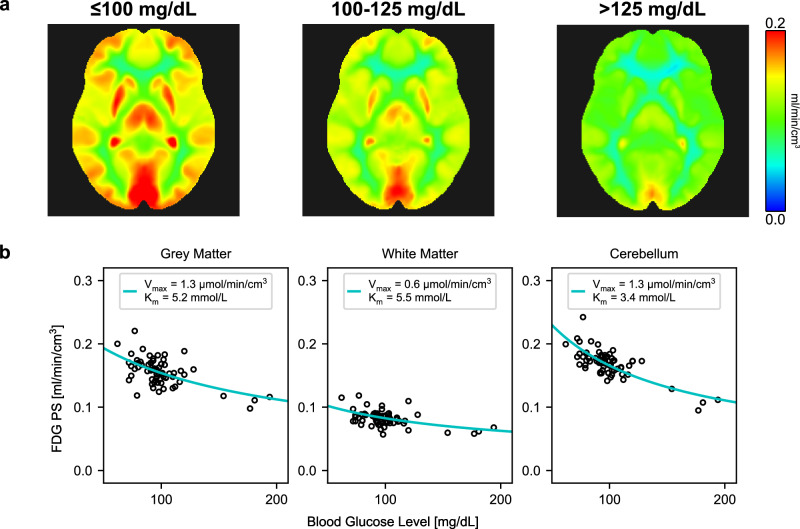
Blood-brain barrier (BBB) permeability-surface area (PS) product of ^18^F-fluorodeoxyglucose (FDG) and blood glucose level. **a** FDG BBB PS parametric images averaged across blood glucose ranges showed that FDG PS decreased with higher blood glucose levels, with notable decreases at hyperglycemia (> 125 mg/dL; *N* = 5). **b** Cohort-level Michaelis-Menten transporter kinetics across 64 analyzed subjects with total-body dynamic FDG positron emission tomography. PS is in units of ml/min/cm^3^.

Chronic hyperglycemia is known to downregulate glucose transporter 1 (GLUT1) expression at the BBB, leading to reduced BBB glucose transport^63^. This may have significant clinical implications in diseases including diabetes mellitus and MASLD in which hyperglycemia is common. In our MASLD analyses, we also found that fasting blood glucose level was a significant covariate between the inflammation groups (*P* < 0.001) and group-level comparisons of FDG PS were not significant after adjusting for blood glucose (*P* = 0.279). Averaged inter-subject FDG BBB PS parametric images for three blood glucose ranges (normal, medium, and high) showed progressively lower FDG BBB PS with higher blood glucose levels (Fig. 6a). Our data suggests that BBB dysregulation may be multifactorial or glucose mediated.

To quantify the relationship between FDG BBB PS and blood glucose, we computed population-based Michaelis-Menten transporter kinetics^64^ by utilizing the range of blood glucose levels (62 to 194 mg/dl) across all 64 analyzed subjects with total-body dynamic FDG-PET (Fig. 6b). The fitted maximal transport rate (V_max_) for FDG was 1.3, 0.7, and 1.3 µmol/min/cm^3^, in grey matter, white matter, and cerebellum, respectively, closely agreeing with preclinical estimates of FDG Michaelis-Menten kinetics^64^ and a human MR spectroscopy study showing brain glucose V_max_ is 2–3× greater in grey matter than that of white matter^65^. The half-saturation constant (K_m_) ranged from 3.4 to 5.5 mmol/L between regions in general agreement with prior work^64^. These data cross-validated the proposed HTR method for FDG BBB PS imaging. While it is difficult to measure regional blood glucose levels in the brain, FDG BBB PS provides detailed spatial information beyond a single blood glucose measure, possibly related to BBB transporter expression and function.

## Discussion

The BBB is the primary site of molecular exchange between the systemic circulation and brain parenchyma; however, in vivo molecular probing of the human BBB has thus far been limited by the lack of efficient translational imaging methods to specifically measure molecular BBB permeability. Herein, we developed a non-invasive multiparametric PET method to measure the molecular BBB PS of radiotracers with a single-tracer dynamic PET scan. We demonstrated regional and voxel-wise measurement of the molecular BBB PS of three PET tracers (^18^F-fluciclovine, ^18^F-FDG, ^11^C-butanol) spanning a wide range of BBB permeabilities. Focusing on FDG, we then demonstrated three clinical applications: BBB PS associations with age in healthy subjects, BBB dysregulation in MASLD-related liver inflammation, and an investigation of FDG BBB PS associations with blood glucose levels. Each of these studies has important implications in studying and characterizing healthy aging, brain-body crosstalk in chronic liver disease^60^, and diabetes, respectively. The results collectively point to the critical need for in vivo molecular probing of the human BBB to elucidate the functional health of this dynamic barrier. We present a paradigm to non-invasively study BBB function at the molecular level with a single dynamic PET scan.

Our non-invasive single-tracer method is a significant advancement over existing methods for imaging BBB PS. Past efforts with DCE-MRI have been limited to inert contrast agents with low extraction fraction^9,10^, mainly assessing the BBB as a structural barrier. The complexity of serial dual-tracer PET imaging^13,15,16^ has limited its widespread use in both preclinical and human imaging research^7,66^ despite the PS product prevailing as the most specific measure of BBB permeability^6^. We show our method can measure BBB PS across three orders of permeability magnitude, opening opportunities to apply this method to the numerous molecular PET tracers already available for research and clinical use and which cross the BBB through a diverse set of transport mechanisms.

The proposed method was enabled by HTR dynamic imaging in combination with advanced kinetic modeling for joint estimation of CBF and tracer-specific BBB transport rate. Such a HTR method was challenging, if not impossible, using past-generation PET scanners due to their poor temporal resolution, insufficient statistical quality of dynamic data, and lack of a reliable image-derived input function^18^. Though our demonstration of molecular BBB PS imaging was performed with an advanced total-body scanner, the clinical and research adoption of this and similar high-sensitivity scanners^30,31,67,68^ is rapidly growing with over fifty installations worldwide. Advances in brain-dedicated PET imagers^68^ and image reconstruction methods^69,70^ are also imminent, bringing higher spatial and temporal resolution for dynamic imaging to enable our HTR kinetic modeling method into broader settings. These advances may also enable lower-dose PET studies, mitigating potential concerns about radiation dose and encouraging broader adoption of our method. Our MASLD study with total-body PET used approximately half the injected activity used for conventional FDG-PET and nevertheless our proposed method was able to detect significant differences in FDG BBB PS across clinically-relevant groups. In the future, we will investigate the feasibility of our proposed method for both regional quantification and voxel-wise parametric imaging with reduced injected activities.

The interpretation of the BBB PS depends on the specialized molecular transport mechanism of the tracer and the vascular environment^1^. For example, ^11^C-butanol freely diffuses across the BBB, leading to an extraction fraction of ≈100% as previously suggested^17,18^ and confirmed with our method. For ^18^F-fluciclovine and ^18^F-FDG, BBB transport is passively facilitated primarily by the sodium-independent L-type large neutral amino acid transporter 1 (LAT1)^71^ and glucose transporter 1 (GLUT1)^72^, respectively. The greater BBB PS of ^18^F-FDG over ^18^F-fluciclovine can partially be explained by the two-order of magnitude greater expression of GLUT1 found over LAT1 in a post-mortem proteomic study in humans^73^. Further quantitation with Michaelis-Menten transporter kinetics could explain differences in molecular PS for facilitative transport^64^. To further characterize the biological significance of the BBB PS, a future study may investigate correlations between regional molecular BBB PS and transporter gene expression such as in the Allen Human Brain Atlas^74^.

In comparison, DCE-MRI measures of BBB permeability mainly represent non-specific leakage of contrast material associated with BBB breakdown^8–10^ as gadolinium contrast agents are not known to cross an intact BBB effectively^5^. Differences in BBB transport mechanisms may explain why BBB permeability changes in different ways for each tracer. DCE-MRI-derived PS was shown to increase with age due to increased vascular leakage^8^ while our normal aging study showed that FDG BBB PS decreases with age possibly due to reduced GLUT1 transporter expression^75^. Although both processes likely occur simultaneously in aging, the resulting changes to DCE-MRI and FDG BBB PS greatly differ in scale (10^−3^ and 10^−1^ ml/min/cm^3^, respectively). The effects of subtle BBB leakage were therefore likely obscured by the much greater changes in FDG BBB PS. Our current approach cannot explicitly differentiate the contribution of each transport mechanism to the measured BBB PS. Using DCE-MRI in conjunction with our PET method may help study molecular permeability when there is a more severe breakdown of the BBB, such as in brain tumours^76^.

Multiparametric imaging of CBF, molecular BBB permeability, and transport can augment a radiotracer’s standard use, opening opportunities otherwise challenged by the complexity of multi-tracer imaging. For instance, impaired CBF, dysregulated BBB permeability and transport, and reprogrammed cellular metabolism (using the standardized uptake value or net uptake rate, K_i_)^28^, which are common markers of neurovascular dysfunction^2^, can now be efficiently assessed with our multiparametric imaging method from a single dynamic FDG-PET scan. Importantly, each parameter represents a distinct physiological feature and a dysfunction of one parameter may point to a specific pathophysiological mechanism, for example, in our MASLD study, where FDG BBB PS differed between severities of liver inflammation but not CBF (Fig. 5). With the prevalence of FDG-PET in oncology, cancer-related cognitive impairment^77^ may be an important future target to study with our multiparametric brain imaging method.

Beyond ^18^F-FDG, our single-tracer method efficiently can add multiparametric depth for studying Alzheimer’s disease with amyloid^78^ and tau^79^ radiotracers (e.g., ^18^F-florbetaben and ^18^F-PI-2620, respectively), synaptic density in major depression with radioligands (^11^C-UCB-J)^80^, and the neuroimmune system with ^18^F-DPA-714 for neuroinflammation^81^ or ^18^F-AraG for imaging T-cell activation^82^. However, radiotracers with radiometabolites may require arterial blood sampling for metabolite correction if they cannot be neglected in the early 2-min dynamic scan or if population-based corrections are insufficient. Our simulation study showed that a systematic bias of approximately 5% was observed for PS, K_1_, and E when neglecting radiometabolites in the arterial input function (Supplementary Fig. 4).

Advanced kinetic models and other approaches to measure CBF from early dynamic imaging have been described previously^18,40,41,44,45^, but had limited applications in PET until recently^24,42,43^ with the advent of high-sensitivity long axial field-of-view PET. Here, we used the AATH model as it modeled both CBF and K_1_ with a relatively simple closed-form time-domain solution^46^. Alternative approaches include the one-barrier distributed parameter model^18,40,44^ and model-independent deconvolution^24,45^. Implementation and parameter estimation with the one-barrier distributed parameter is challenging, and model-independent deconvolution requires regularization^24,45^, possibly biasing parameter estimates. Estimating K_1_ with model-independent deconvolution also requires an alternative method such as standard compartmental modeling or an impulse response function fitting procedure^24^. However, deconvolution does not require the specific kinetic model to be known, was demonstrated on five different PET tracers, and is not limited to only the first two minutes as in our approach^24^. The use of the first two minutes for our method was guided by prior studies using early-dynamic FDG-PET to approximate blood flow using FDG K_1_^83,84^ as well as to minimize the effect of phosphorylation in the model^34,85^. In future work, we will optimize the protocols and compare the strengths and weaknesses of each method in measuring and quantifying CBF and PS. Furthermore, the AATH model will be extended to allow additional tissue compartments such as the phosphorylation of ^18^F-FDG.

A major limitation of this work is the lack of ground truth values in humans for validation of our PS measurements. This in part reflects the practical difficulties of measuring the molecular BBB PS of radiotracers in humans using existing methods. However, several of our results characterized the proposed method indirectly. First, the measured CBF was consistent across three very different radiotracers and were all comparable to population-based values reported in the literature^17,18,26^. The method was also able to accurately estimate an extraction fraction equal or close to 100% for the freely diffusible tracer ^11^C-butanol^17^ and a small extraction fraction for ^18^F-fluciclovine, the latter known to have low uptake in the brain^47,53^. Second, FDG BBB PS in healthy subjects was also comparable to those reported in the literature by other methods^12,13^. Our observed negative association of FDG BBB PS with age is concordant with evidence that the expression of GLUT1 at the BBB decreases with age^75^. Third, we also used the FDG BBB PS estimates to derive its theoretical Michaelis-Menten transporter kinetics, which agreed with those derived from preclinical and human data^64,65,86^. These results increase confidence in our proposed method. To further validate our method, future work includes a test-retest study to characterize the variability and repeatability of BBB PS estimates, validating the quantitative accuracy of CBF estimates against the gold standard (e.g., by ^11^C-butanol PET)^17,26^, as well as experiments manipulating CBF, BBB transporters (e.g., inducing hyperglycemia for ^18^F-FDG), or transiently opening the BBB using MR-guided focused ultrasound^87^ in the same subject to study resulting changes in BBB PS and other transport parameters. As well, it will be necessary to demonstrate our method across a wide range of in vivo physiological and pathophysiological conditions, such as in cerebral small vessel disease with prolonged T_c_^88^ or for radiotracers that are rapidly excluded by the BBB.

This study also had other limitations. It is possible that our PS estimates not only comprise molecular transport through the BBB, but also through parenchymal cell membranes. This challenge persists in other methods^7,12,89^ and we mitigated this by studying only the first two minutes of the dynamic scan. Our PS estimates may therefore be marginally overestimated. Future work will comprise further optimization of scan duration and extension of the HTR kinetic model from one tissue compartment to two tissue compartments to better capture the full kinetics of metabolic tracers like ^18^F-FDG. In addition, our studies of age, MASLD, and blood glucose were exploratory and not specifically designed to answer a biological hypothesis. For example, our pilot study of MASLD showed an association of FDG BBB PS with liver inflammation but the result may be confounded by blood glucose. Liver inflammation, insulin resistance, and diabetes may collectively contribute to the dysregulation of the BBB^3,4,90^ and the complex multivariate interactions could not be fully resolved with our relatively small sample size. Our main aim was instead to showcase the potential of our method. Future studies with additional controls and complementary data will better elucidate the biological and clinical significance of the molecular BBB PS. Lastly, we did not scan the same participant with each of our three investigated radiotracer when comparing BBB PS across tracers. As such, subject-specific CBF and extraction may have potentially confounded our PS comparisons, but these effects were likely small relative to the order of magnitude differences in BBB PS we observed between radiotracers.

The proposed method has many potential applications beyond the demonstrations in this paper. In drug development, PS remains the key parameter describing drug permeability across the BBB^6,7^ and our method may revitalize its adoption in both preclinical drug development studies and in human studies. Broader quantification of molecular permeability may also support the development of in silico methods for drug delivery and discovery^7,66,89^. Beyond the brain, quantifying vascular permeability may add a dimension to study cardiovascular disease^91^, design treatment delivery systems, and monitor the vascular toxicity of systemic cancer therapies^92^. Furthermore, changes in gut vascular permeability have been observed due to pathogenic bacteria^93^ and gut-brain interactions have shown a dysregulation of the BBB in mice lacking gut microbiota^94^. Measuring molecular permeability at systemic capillaries such as at gut vasculature^93^, liver sinusoids^95^, and the blood-tumor barrier^76^ may require further development as transport mechanisms likely differ relative to the highly controlled BBB^1^. Such methodological advances in combination with total-body PET may enable vascular permeability studies along the brain-body axis, with potential applications to design and monitor the delivery of systemic therapies. Thus, our developed method may serve as a powerful translational framework to study the role of molecular barrier function in neurological and systemic diseases.

## Methods

### Study design

This study was approved by Institutional Review Board (IRB) at the University of California, Davis and written informed consent was obtained for all study participants. The primary objective of this study was to develop a single-tracer method of quantifying and imaging the BBB PS of PET radiotracers and to demonstrate its importance in characterizing molecular BBB permeability. To this end, this study was divided into five experiments. The first experiment focused on demonstrating the need and capability of high temporal resolution (HTR) dynamic imaging and more advanced kinetic modeling enabled by total-body PET to jointly estimate CBF and tracer-specific BBB transport rate K_1_. The second experiment was to use CBF and K_1_ to quantify and image differences in BBB PS between PET radiotracers. We included HTR dynamic PET studies scanned with three radiotracers thought to encompass a wide range of BBB PS due to their previously reported BBB transport rate values^12,17,47^. In the third experiment, we investigated the molecular BBB PS of ^18^F-FDG and its association with aging. In the fourth experiment, we conducted an exploratory analysis of FDG BBB PS to investigate a potential brain-body crosstalk in patients with MASLD enrolled for an imaging trial of liver inflammation. Lastly, the relationship between FDG BBB PS and fasting blood glucose was investigated using the pooled healthy subjects and MASLD patients.

### High-temporal resolution dynamic imaging with total-body PET

Total-body positron emission tomography (PET) was conducted on all human participants using the 194-cm axial field of view uEXPLORER total-body PET/CT system (United Imaging Healthcare). The uEXPLORER PET/CT system has exceptional detection sensitivity and high spatial resolution (≈ 3.0 mm full width at half maximum resolution by the NEMA standard). Its performance characteristics^30^ and ability to perform HTR dynamic imaging have been reported previously^33–35^. All participants received either an ultra low-dose or low-dose total-body CT (140 kVp with dose modulation at 5 or 50 mAs maximum tube current-exposure time product, respectively, corresponding to effective doses of ≈1 mSv or ≈10 mSv) for attenuation correction and anatomical localization. Dynamic PET imaging commenced immediately prior to bolus injection of the radiotracer. The bolus was rapidly injected by hand in 1 to 2 s for radiotracer volumes <1 ml for ^18^F-FDG, <2 ml for ^18^F-fluciclovine, and <6 ml for ^11^C-butanol. We pooled total-body dynamic PET scans from several human studies with IRB approval and written informed consent from all study participants.

For brain kinetic modeling of each dynamic PET scan, we performed HTR reconstructions of the first two minutes (framing: 60× s, 30× s) using vendor-provided reconstruction software and standard corrections for attenuation, scatter, randoms, dead time, and decay^30^. Specifically, a time-of-flight ordered subset expectation-maximum algorithm with 4 iterations and 20 subsets was used to reconstruct each dynamic image. An image-derived arterial input function^29,35,36^ was non-invasively obtained from the ascending aorta.

The effective dose of PET scans varied depending on the radiotracer and injected activity. For the investigated radiotracers in this study, approximate effective doses per unit activity were 19 µSv/MBq for ^18^F-FDG, 22 µSv/MBq for ^18^F-fluciclovine, and 4 µSv/MBq for ^11^C-butanol^11^, resulting in a mean effective dose of 7.0 mSv, 6.8 mSv and 1.1 mSv for the three studies, respectively. These effective doses are well within the acceptable range for healthy subjects as compared to the average annual natural background radiation of 3.1 mSv in the United States. Protocols were approved by our IRB and ethics committee.

### High-temporal resolution kinetic modeling and measuring BBB PS

The early kinetics of a radiotracer in the brain were quantified by using the AATH model^46^ applied on the first two minutes of HTR dynamic PET data. The AATH model offers a closed-from time-domain solution to a distributed kinetic model comprised of a spatiotemporally distributed intravascular space and a compartmental extravascular space^46^. The impulse response function of the AATH model is (Supplementary Fig. 7a)

2 $${R}^{{{\rm{AATH}}}}\left(t\right)=\left\{\begin{array}{cc}{{\rm{CBF}}},& 0\le t < {T}_{c},\\ {K}_{1}{e}^{-{k}_{2}\left(t-{T}_{c}\right)},& t\ge {T}_{c},\end{array}\right.$$

2a $${R}^{{{\rm{AATH}}}}\left(t\right)={{\rm{CBF}}}\cdot \left[H\left(t\right)-H\left(t-{T}_{c}\right)\right]+{K}_{1}{e}^{-{k}_{2}\left(t-{T}_{c}\right)}H\left(t-{T}_{c}\right),$$

where CBF is the cerebral blood flow (ml/min/cm^3^ voxel), *T*_*c*_ is the mean vascular transit time (s), *K*_1_ is the BBB transport rate (ml/min/cm^3^ voxel) of the radiotracer and equal to the product of CBF and extraction fraction $\left({K}_{1}={{\rm{CBF}}}\cdot E\right)$, *k*_2_ is in BBB clearance rate (min^−1^), and $H(t)$ is the Heaviside step function. This solution describes a vascular phase $\left(0\le t < {T}_{c}\right)$ during which time the tracer traverses the intravascular space while permeating to the extravascular space. Of note, the mean vascular transit time T_c_ describes the average time required for tracer to traverse the entire intravascular volume residing in a voxel, including arteries, arterioles, capillaries, venules, and veins. The tissue phase $\left(t\ge {T}_{c}\right)$ follows and describes the return of extracted tracer to the intravascular space and subsequent venous clearance. The cerebral blood volume fraction is accordingly the product of CBF and mean vascular transit time $\left({v}_{b}={{\rm{CBF}}}\cdot {T}_{c}\right)$, and accounts for the total intravascular volume fraction.

For a general arterial input, ${C}_{a}(t)$, the tissue time-activity curve is

3 $$Q\left(t\right)={C}_{a}\left(t-{t}_{d}\right)\otimes R\left(t\right)$$

where *t*_*d*_ is a time delay parameter accounting for the time difference between tracer arrival at the ascending aorta and the regional cerebral artery. A parametric form of the AATH time-activity curve can be derived by substituting (2) into (3):

4 $$\begin{array}{c}Q\left(t\right)={{\rm{CBF}}}\cdot \left[{\int }_{{t}_{d}}^{t}{C}_{{wb}}\left(\tau -{t}_{d}\right)\,d\tau -{\int }_{{t}_{d}+{T}_{c}}^{t}{C}_{{wb}}\left(\tau -{t}_{d}-{T}_{c}\right)\,d\tau \right]+\\ {K}_{1}{\int }_{{t}_{d}+{T}_{c}}^{t}{C}_{p}\left(\tau -{t}_{d}-{T}_{c}\right){e}^{-{k}_{2}\left(t-\tau \right)}H\left(t-\tau \right)\,d\tau \end{array}$$

where ${C}_{{wb}}(t)$ and ${C}_{p}(t)$ are the whole-blood and plasma arterial input functions, respectively, and the distinction accounts for whole-blood flowing through the blood vessels whereas tracer exchange with the extravascular space occurs in plasma. Each integral is zero when *t* is less than its respective lower limit of integration. The first integral describes the accumulation of tracer in blood and tissue due to its arterial delivery by CBF. The second integral describes the tracer’s venous clearance by CBF after the mean vascular transit time, T_c_. The third integral describes tracer extraction across the BBB, return of the extracted tracer back to blood, and its subsequent venous clearance from the voxel volume.

We used the least-squared curve fitting formulation with a basis function algorithm to estimate the parameters of the time delay-corrected AATH model:

5 $${{\boldsymbol{\theta }}}=\mathop{{{\rm{argmin}}}}\limits_{{{\boldsymbol{\theta }}}}\mathop{\sum}\limits_{m=1}^{M}{{w}_{m}\left(Q\left({t}_{m}\right)-\hat{Q}\left({t}_{m}\right)\right)}^{2}$$

where ${{\boldsymbol{\theta }}}=\left[{{\rm{CBF}}},\,{K}_{1},\,{k}_{2},\,{T}_{c},\,{t}_{d}\right]$ are the model parameters to be estimated, $Q(t)$ and $\hat{Q}(t)$ are the measured and fitted time-activity curves, respectively, *M* is the number of time frames, *t*_*m*_ is the midpoint time of the *m*th frame, and *w*_*m*_ is the residual weighting factor (${w}_{m}=1$ in this work). Specifically, we performed a naïve grid search of ${t}_{d}\in [{\mathrm{0,16}}]\,{{\rm{s}}}$ and ${T}_{c}\in \left[3,\,16\right]\,{{\rm{s}}}$ at 0.25 s intervals and used 100 logarithmically spaced ${k}_{2}\in \left[0.006,\,3\right]\,{\min }^{-1}$, resulting in 344,500 combinations of ${t}_{d}$, ${T}_{c}$, and ${k}_{2}$. Substituting the grid-searched values of ${t}_{d}$, ${T}_{c}$, and ${k}_{2}$ into Eq. 4 results in CBF and ${K}_{1}$ as the remaining two unknowns. ${CBF}$ and ${K}_{1}$ are linear scaling factors and were estimated by a non-negative linear least squares algorithm^96^, which minimized the sum of squared deviation between the measured $Q(t)$ and the fitted AATH time-activity curve. Based on time-delay and mean vascular transit time estimates from regional kinetic analysis, we reduced the grid search interval of ${t}_{d}$ and ${T}_{c}$ to 0.5 s to reduce computation time for voxel-wise parametric imaging. We interpreted the CBF term as the intravascular basis and the ${K}_{1}$ term as the extravascular tissue basis to resolve the distributions of the fitted time-activity curve.

The standard one-tissue compartment (S1TC) model was used for comparison against existing methods. The impulse response function of the S1TC model is (Supplementary Fig. 7b)

6 $${R}^{{{\rm{S}}}1{{\rm{TC}}}}\left(t\right)=\left\{\begin{array}{cc}{v}_{b} & t=0\\ {K}_{1}{e}^{-{k}_{2}t} & t > 0\end{array}\right.$$

6a $${R}^{{{\rm{S}}}1{{\rm{TC}}}}\left(t\right)={v}_{b}\delta \left(t\right)+{K}_{1}{e}^{-{k}_{2}t}$$

where ${v}_{b}$ is the cerebral blood volume fraction (ml/cm^3^ voxel). The S1TC differs from the AATH model as it assumes instantaneous distribution of tracer in the intravascular space and neglects the finite transit time required for tracer to traverse the blood vessel volume (i.e., T_c_ = 0). As such, the S1TC response function lacks a finite-length vascular phase and the model describes that tracer is immediately cleared from tissue at $t > 0$. This is consistent with the observation that the AATH impulse response function is equal to that of the S1TC model when substituting ${CBF}={v}_{b}/{T}_{c}$ in Eq. (2) and taking the limit as T_c_ approaches 0.

A parametric form of the S1TC time-activity curve can be derived by substituting (6) into (3):

7 $${Q}^{{{\rm{S}}}1{{\rm{TC}}}}\left(t\right)={v}_{b}{C}_{{wb}}\left(t-{t}_{d}\right)+{K}_{1}{\int }_{{t}_{d}}^{t}{C}_{p}\left(\tau -{t}_{d}\right){e}^{-{k}_{2}\left(t-\tau \right)}H\left(t-\tau \right)\,d\tau$$

Of note, the tissue volume fraction, ${v}_{t}=1-{v}_{b}$, commonly seen as a scaling factor of the second term of Eq. (7), is included as a part of K_1_. This was chosen to be consistent with the original AATH model^46^ and is also commonly used in DCE-MRI studies^44,97^. K_1_ is therefore expressed per unit voxel volume as opposed to tissue volume. CBF and PS also follow this convention. As the brain has a cerebral blood volume fraction of ≈5%^19,25^, differences in absolute values due to following this convention is negligible.

The four parameters of the time delay-corrected S1TC model were estimated in a manner similar to the AATH model by a basis function method where ${v}_{b}$ and ${K}_{1}$ were estimated by a non-negative linear least squares algorithm^96^. For all investigated tracers and for both AATH and S1TC methods, we assumed an absence of metabolites^26,47,53^ and that the whole-blood tracer activity was equal to that in blood plasma over the first two minutes of the dynamic PET scan. Similar to a recent study that also used 1-s high-temporal resolution dynamic PET^24^, we assumed that arterial input function dispersion was negligible due to the relatively short distance between the ascending aorta and the brain.

An advantage of the AATH model^46^ and other distributed models^40^ is their ability to jointly estimate CBF and the tracer-specific BBB transport rate K_1_ from HTR dynamic PET data whereas the S1TC model can only estimate K_1_. The PS product (Eq. (1)) can then be calculated from the AATH CBF and AATH K_1_ using a single-tracer dynamic PET scan by rearranging the Renkin-Crone equation^49,50^

8 $$E\equiv {K}_{1}/{{\rm{CBF}}}=1-{e}^{-{PS}/{{\rm{CBF}}}},$$

where *E* is the extraction fraction.

### Imaging the molecular BBB PS of different PET tracers

To demonstrate that our method can measure across different radiotracers with a wide range of BBB permeabilities, we included fifteen age-matched participants scanned with one of either ^18^F-fluciclovine, ^18^F-FDG, or ^11^C-butanol (*N* = 5 each). No statistical method was used to predetermine sample size and participants were an age-matched subset of a study of biochemically recurrent prostate cancer (^18^F-fluciclovine; UC Davis IRB # 1470016) and healthy volunteers (^18^F-FDG IRB #1714742; ^11^C-butanol IRB #1783992). These three radiotracers were chosen to span a wide range of low to very high BBB PS as expected from their previously reported BBB transport rate values^12,17,47^. ^18^F-fluciclovine is a radiolabeled analogue of leucine, an essential amino acid, which has demonstrated low brain uptake and BBB transport rate on the order of 10^−2^ ml/min/cm^3^ ^47,53^. ^18^F-FDG is a glucose analogue with moderate BBB PS on the order of 10^−1^ ml/min/cm^3^^12,13^. ^11^C-butanol is a lipophilic alcohol and considered a favorable flow radiotracer due to its predictably high extraction fraction of ≈100% owing to its free apparent diffusion across the BBB^17,18^. ^18^F-fluciclovine, ^18^F-FDG, and ^11^C-butanol PET studies were scanned using a mean (± standard deviation) activity of 309 ± 8 MBq (range: 298 to 318 MBq), 370 ± 16 MBq (349 to 395 MBq), and 282 ± 10 MBq (267 to 296 MBq). The mean age of the participants was 64.4 ± 6.7 y, 63.6 ± 6.9 y, and 61.6 ± 6.4 y for ^18^F-fluciclovine, ^18^F-FDG, and ^11^C-butanol, respectively (global mean: 63.2 ± 6.3 y; range: 54 to 73 y) and there were no significant differences in age between tracer groups (*P* = 0.796). Thirteen of fifteen participants were male, and both female participants were scanned with ^11^C-butanol.

### Model comparison and practical identifiability analysis

To assess the need for HTR dynamic imaging, our original measured regional time-activity curves were frame averaged at 1 to 10 s intervals and fitted with the AATH and S1TC models. The AIC^48^ was computed to statistically determine which model produced a better fit at different temporal resolutions. A lower AIC indicated better statistical fit after adjusting for the trade-off between model complexity and residual model fitting error. The AIC was computed as

9 $${AIC}=M{\mathrm{ln}}\frac{{\sum }_{m=1}^{M}{\left(Q\left({t}_{m}\right)-\hat{Q}\left({t}_{m}\right)\right)}^{2}}{M}+2n+\frac{2n(n+1)}{M-n-1}$$

where *n* is the number of model parameters ($n=5$ for AATH, $n=4$ for S1TC).

Practical identifiability analysis was conducted to assess the reliability of our parameter estimates^52^. For each subject’s AATH fitted curves, 1024 realizations of time-varying noise were added and AATH parameters were estimated from these simulated noisy time-activity curves. Parametric error mean and standard deviation were computed across the 1024 noise realizations. We reported error mean and standard deviation for each radiotracer group and brain region. Time-varying noise accounted for frame duration, radionuclide decay, and time-varying activity concentration, and the noise was scaled by the estimated standard deviation of the normally distributed fitting residuals as described in prior work^52^.

Additional simulations were conducted to determine the practical identifiability of PS, K_1_, CBF, and E when simulating different values of extraction fraction, T_c_, and CBF. Using an average ascending aorta curve determined from our dataset, we simulated tissue time-activity curves using the AATH model with extraction fraction varied from 0.01 to 0.99, T_c_ from 3 to 15 s, and CBF at 0.25, 0.50, and 0.75 ml/min/cm^3^. For this experiment, time delay t_d_ was fixed at 2 s and extravascular distribution volume V_e_ was also varied from 0.25 to 1.0 ml/cm^3^ from which k_2_ = K_1_/V_e_ was derived. A noise scale factor of 4.8 were used as estimated from the measured regional time-activity curves as described. This experiment also used 1024 noise realizations to compute parametric error mean and standard deviation for each parameter set. We also studied the consistency of PS estimates when CBF was manipulated while PS, t_d_, T_c_, and k_2_ were fixed at 0.15 ml/min/cm^3^, 2 s, 5 s, and 0.25 min^−1^, respectively.

Sensitivity analysis was conducted as previously described^98^ to study the covariance of AATH model parameters. Briefly, normalized sensitivity functions were computed using:

10 $${\hat{S}}_{{\theta }_{k}}(t)=\frac{\partial Q(t)/Q(t)}{\partial {\theta }_{k}/{\theta }_{k}}$$

where ${\theta }_{k}$ is an AATH model parameter. The partial derivatives were numerically estimated using a ± 2.5% change in ${\theta }_{k}$. A sensitivity matrix with elements $S{M}_{{ij}}$ was then obtained by numerically integrating the product of sensitivity function pairs, ${S}_{{\theta }_{i}}(t)$ and ${S}_{{\theta }_{j}}(t)$. The parameter correlation matrix was calculated by inverting the sensitivity matrix and normalizing each element by the square root of the product of the corresponding row and column diagonal elements. Supplementing this first-order sensitivity analysis, we also computed the Pearson coefficient between each pair of parameters estimated across 1024 noise realizations in our practical identifiability experiment to assess whether the estimated value of one parameter is correlated with the estimation of another.

We also studied the effect of the arterial input function on the practical identifiability of PS, K_1_, E, and CBF. First, we studied the effect of plasma radiometabolites on parameter estimation accuracy. Using a population-based parent fraction of ^18^F-florbetaben^54^, we generated a representative metabolite-free plasma input function and used it and the whole-blood arterial input function to simulate AATH time-activity curves without contamination of radiometabolites. Here we assumed a representative case where metabolites do not cross the BBB. We then conducted practical identifiability analysis with model parameters estimated using the metabolite-corrected and uncorrected plasma input functions. Second, we studied how the shape of the arterial input function may affect parameter estimation accuracy. Using an average ascending aorta arterial input function from our cohort, we simulated dispersed arterial input functions using a mono-exponential dispersion function^99^:

11 $${C}_{d}\left(t\right)={C}_{a}\left(t\right)\otimes {k}_{d}{e}^{-{k}_{d}t}$$

where ${C}_{d}(t)$ is the dispersed arterial input function and ${k}_{d}$ is the dispersion rate constant. Three levels of dispersion (*k*_*d*_ = 30, 10, 5 min^−1^) were simulated for this experiment. Practical identifiability analysis with 1024 noise realizations was conducted to benchmark the accuracy of parameter estimates when simulating AATH time-activity curves with each dispersed arterial input function.

### FDG BBB PS in aging

To investigate the association of FDG BBB PS with age, we included thirty-four healthy subjects (21 females, 13 males; mean age: 51.0 ± 13.3 years, range: 26 to 78 years) who received 60-min total-body dynamic FDG-PET (mean activity: 358 ± 33 MBq) pooled without exclusion from two healthy volunteer studies (IRB #1341792, 1714742). No data were excluded from the analyses. We explored associations between age and FDG BBB PS, K_1_, and CBF by the Pearson coefficient and linear regression. Linear regression slopes were reported to indicate parametric change per year change in age. Percent changes were computed by regressing age with the logarithm of the parameter. We compared parametric images across three age groups (25–45 years, *N* = 9; 45–60 years, *N* = 14; ≥60 years, *N* = 11), aiming for a similar number of subjects in each group.

The five FDG-PET participants from the three-radiotracer experiment were a subset of those from this experiment. All healthy subjects had no history of major disease within the last five years or ongoing acute inflammation. Participants fasted for at least 6 h prior to FDG-PET (mean: 11 ± 2 h) and had a mean fasting blood glucose level of 91 ± 12 mg/dl (range: 62–116 mg/dl). The mean BMI was 28.0 ± 5.6 kg/m^2^ (range: 17.5–37.6 kg/m^2^) with approximately equal distributions in the number of subjects with healthy weight (18.5 to 24.9 kg/m^2^; *N* = 11), overweight (25.0 to 29.9 kg/m^2^; *N* = 10), and obesity (≥ 30 kg/m^2^; *N* = 12).

### FDG BBB PS in MASLD

This opportunistic analysis of BBB permeability in systemic disease included 30 consecutive patients (mean age 53.2 ± 7.3 y; 8 males, 22 females) receiving liver biopsy and 60-min total-body dynamic FDG-PET (mean activity: 186 ± 13 MBq) between July 2020 and February 2023 (IRB #840422). All consecutive patients were included without a predetermined sample size. Liver biopsy and FDG-PET were obtained within a median of 5.0 (IQR: 2.1 to 11.6) weeks of one another. An expert pathologist graded the liver biopsies by the MASLD activity score (MAS; ranging from 0 to 8 where a higher value indicates greater severity of MASLD), equal to the sum of sub-component scores for steatosis (0 to 3), lobular inflammation (0 to 3), and ballooning degeneration (0 to 2)^62^. Patients fasted for at least 6 h prior to FDG-PET.

Here, we dichotomized patients into mild and severe lobular inflammation biopsy scores (e.g., mild included scores 0 and 1, and severe included scores 2 and 3) and their FDG early brain kinetic parameters were compared. To serve as a healthy control group, we also included 13 age-matched healthy subjects from the thirty-four participants described in the healthy aging experiment. These participants were assumed to have no abnormal liver findings as supported by qualitative readings of their FDG-PET and CT scans by a nuclear medicine physician. The mean age was 49.6 ± 12.5 y, 52.4 ± 13.0 y, and 51.0 ± 11.0 y in the healthy control (3 males, 10 females), mild lobular inflammation (5 males, 8 females), and severe lobular inflammation (3 males, 14 females) groups, respectively, and did not significantly differ by a one-way ANOVA (*P* = 0.84). We used the univariate general linear model tool in SPSS for multivariable regression analysis to adjust for blood glucose level when comparing FDG brain kinetics between age-matched healthy controls (mean blood glucose level: 90.0 ± 10.8 mg/dl; *N* = 13), mild lobular inflammation (98.0 ± 15.8 mg/dl; *N* = 13), and severe lobular inflammation (118.6 ± 35.8 mg/dl; *N* = 17). We also examined groupings by fasting blood glucose levels between 70 and 100 mg/dl (normoglycemia; *N* = 25), 100 to 125 mg/dl (*N* = 13), and >125 mg/dl (hyperglycemia; *N* = 5).

### Michaelis-Menten transporter kinetics with FDG BBB PS

To cross-validate our FDG BBB PS estimates against literature values, we computed Michaelis-Menten transporter kinetic parameters across all total-body dynamic FDG-PET scans analyzed in our study. A total of 64 subjects were included of whom 34 were healthy subjects (mean age 51.0 ± 13.3 y; 13 males, 21 females) and 30 were patients with MASLD (mean age 53.2 ± 7.3 y; 8 males, 22 females) as described in the previous subsections. Blood glucose levels were measured by a fingerstick test prior to FDG-PET and ranged from 62 to 194 mg/dl in our cohort. We used a non-saturable Michaelis-Menten facilitative transporter kinetics model described previously^64^:

12 $${{\rm{PS}}}=\frac{{V}_{\max }}{{K}_{m}+\frac{{K}_{m}}{{K}_{m,{glc}}}\left[{Glc}\right]}+{K}_{d}$$

where ${V}_{\max }$ is the maximal transport rate (µmol/min/cm^3^) of FDG, ${K}_{m}$ is the half saturation constant (mmol/l) of FDG, ${K}_{m}/{K}_{m,{glc}}$ is the ratio of half saturation constants between FDG and glucose, and $[{Glc}]$ is the blood glucose concentration (mmol/l) in competitive transport with FDG, and ${K}_{d}$ is the non-saturable transport rate (ml/min/cm^3^). A Levenberg-Marquardt algorithm with a maximum of 100 iterations was used to solve for ${V}_{\max }$, ${K}_{m}$, and ${K}_{m}/{K}_{m,{glc}}$ using initial parameters 1.0 µmol/min/cm^3^, 5.0 mmol/L, and 1, respectively. Due to the relatively narrow range of blood glucose levels available in this study, we fixed ${K}_{d}$ to 0.022 ml/min/cm^3^ based on prior data^64^.

### Image analysis

Regions of interest were delineated using 3D Slicer^100^ and by referring to a combination of dynamic and static PET frames and the attenuation correction CT. An image-derived input function was obtained from the ascending aorta for kinetic analysis of all radiotracers. For regional kinetic analysis, regions of interest in the grey matter, white matter, and cerebellum were manually segmented to extract average regional time-activity curves. Cerebellar grey and white matter were not explicitly distinguished in our segmentations. Large cerebral vessels such as at the Circle of Willis and sagittal sinus were avoided. For segmentation, we used a circular brush and the adjusted brush diameter according to the subject-specific size of the anatomical region. The median region of interest volume was ≈70 cm^3^ for brain subregions and 6 cm^3^ for the image-derived input function.

Voxel-wise parametric imaging was performed with the basis function method on the reconstructed dynamic images of 2.344-mm isotropic voxels. Dynamic images and generated parametric images were smoothed using the post-reconstruction kernel method, which is equivalent to a type of nonlocal means noise reduction^101,102^. The kernel matrix was built for each PET scan from four composite image priors derived from the full dynamic study and 49-nearest neighbours within a 9 × 9 × 9 voxel space. For visualization, parametric images were aligned to the Montreal Neurological Institute (MNI)-152 space^57,58^ using the nifty_reg registration toolbox^103,104^. For registration, the two-minute static PET images were cropped to the brain and were used to compute rigid and affine transformations to the MNI-152 space. These computed transformations were then used to resample the parametric images to the MNI-152 space. Deformable registration was only used when generating group-averaged parametric images. For ^11^C-butanol, which often had K_1_ estimates equal to CBF, PS maps were generated by clipping extraction fraction values to 99.9% to avoid indeterminate outputs. This was only for visualization purposes and we accordingly did not quantify the PS of ^11^C-butanol.

### Statistical analysis

Statistical analyses were performed with IBM SPSS Statistics 29 using a two-tailed alpha of 0.05 for statistical significance. A one-way analysis of variance (ANOVA) with post hoc Bonferroni-corrected pairwise comparisons was used to compare differences in means between groups with more than two categories. A *t*-test was used to compare the molecular BBB PS of ^18^F-fluciclovine and ^18^F-FDG due to the absence of PS values for ^11^C-butanol. Pearson coefficients were computed with two-tailed significance testing.

### Reporting summary

Further information on research design is available in the Nature Portfolio Reporting Summary linked to this article.

## Supplementary information

Supplementary Information

Reporting Summary

Transparent Peer Review file

## Source data

Source Data

## Data Availability

All the data generated in this study are present in the paper or the Supplementary Materials. Source Data are provided with this paper. The raw data of human subjects used in this paper are protected and are not available due to data privacy laws. Image data can be provided pending scientific review and a completed data transfer agreement. Requests for data should be submitted to G.W. A response is expected within 1–2 weeks. Source data are provided with this paper.

## Code availability

Python code for kinetic analysis using the AATH model can be downloaded from Github (https://github.com/kjch03/bbb-permeability-pet) and Zenodo (10.5281/zenodo.14954958)^105^ with instructions and testing examples provided.
