# Supplementary material for: Quantitative PET imaging and modeling of molecular blood-brain barrier permeability
Source: Nat Commun. 2025 Mar 30;16:3076. doi: 10.1038/s41467-025-58356-7 (PMC11955546; doi:10.1038/s41467-025-58356-7)
Supplement: Supplementary file 1 — Supplementary Information [file 41467_2025_58356_MOESM1_ESM.pdf]

Quantitative PET imaging and modeling of molecular blood-brain barrier permeability

Supplementary Materials

Supplementary Table 1. Average ± standard deviation of early kinetics across PET tracers and brain regions (N=5 per tracer).

	^{18}F-Fluciclovine			^{18}F-FDG			^{11}C-Butanol		
	GM	WM	CB	GM	WM	CB	GM	WM	CB
PS	0.017±0.003	0.009±0.002	0.021±0.004	0.142±0.011	0.088±0.010	0.168±0.011		*	
K_1	0.017±0.003	0.009±0.002	0.020±0.004	0.121±0.010	0.067±0.007	0.138±0.009	0.463±0.114	0.181±0.038	0.414±0.072
CBF	0.510±0.115	0.179±0.035	0.423±0.101	0.448±0.084	0.158±0.040	0.416±0.054	0.469±0.112	0.183±0.036	0.441±0.054
E	0.035±0.012	0.051±0.010	0.049±0.014	0.276±0.038	0.437±0.082	0.334±0.036	0.986±0.031	0.988±0.027	0.935±0.073
CBV	0.038±0.006	0.019±0.003	0.034±0.007	0.034±0.006	0.015±0.003	0.029±0.003	0.077±0.018	0.029±0.005	0.053±0.028
T_c	4.6±0.8	6.6±0.9	5.0±1.1	4.6±0.5	6.2±2.1	4.3±0.6	9.9±0.2	9.5±0.8	7.2±3.5
t_d	2.2±0.3	1.9±0.5	2.7±0.7	2.4±0.3	2.4±0.4	3.5±0.4	2.2±0.4	2.3±0.5	2.9±0.7

* ^{11}C-Butanol PS was not reported due to estimated *E* = 1 leading to indeterminate values (Equation (1))

GM indicates grey matter; WM, white matter; CB, cerebellum; FDG, fluorodeoxyglucose; PS, permeability-surface area product [ml/min/cm^3]; K_1, blood-brain barrier transport rate [ml/min/cm^3]; CBF, cerebral blood flow [ml/min/cm^3]; E, extraction fraction; T_c, mean vascular transit time [s]; CBV, cerebral blood volume [ml/cm^3]; t_d, time delay [s]

Supplementary Table 2. Practical identifiability analysis of molecular BBB transport kinetics of three radiotracers.

Parameter	Mean ± Standard Deviation [%]		
	**^{18}F-fluciclovine**	**^{18}F-FDG**	**^{11}C-butanol**
PS	0.2±11.5	–0.1±3.4	–––
CBF	1.8±12.2	1.2±11.5	2.0±8.3
K_1	0.2±11.3	–0.1±2.2	–0.1±2.0
E	–0.5±13.9	–0.1±10.8	–1.5±6.3

Error percent mean and standard deviation of the permeability-surface area (PS) product, blood-brain barrier transport rate K_1, cerebral blood flow (CBF), and radiotracer extraction fraction (E) for the three investigated radiotracers, ^{18}F-fluciclovine, ^{18}F-fluorodeoxyglucose (FDG), and ^{11}C-butanol. The identifiability of PS could not be computed for ^{11}C-butanol due to its median extraction fraction of 100%, which leads to indeterminate PS values (Equation (1)). A positive value indicates our method overestimated the true value.

Supplementary Table 3. Correlation matrix of the adiabatic approximation to the tissue homogeneity (AATH) model parameter sensitivity functions

	t_d	T_c	CBF	K_1	k_2
t_d	1.00	–0.74	0.86	0.11	0.08
T_c		1.00	–0.85	–0.44	–0.32
CBF			1.00	0.14	0.11
K_1				1.00	0.92
k_2					1.00

t_d indicates time delay; T_c, mean vascular transit time; CBF, cerebral blood flow; K_1, blood-brain barrier transport rate; k_2, blood-brain barrier clearance rate. Representative model parameters t_d = 2 s, T_c = 5 s, CBF = 0.50 ml/min/cm^3, K_1 = 0.125 min/min/cm^3, and k_2 = 0.25 min^{-1} were used, similar to that of ^{18}F-fluorodeoxyglucose (FDG).

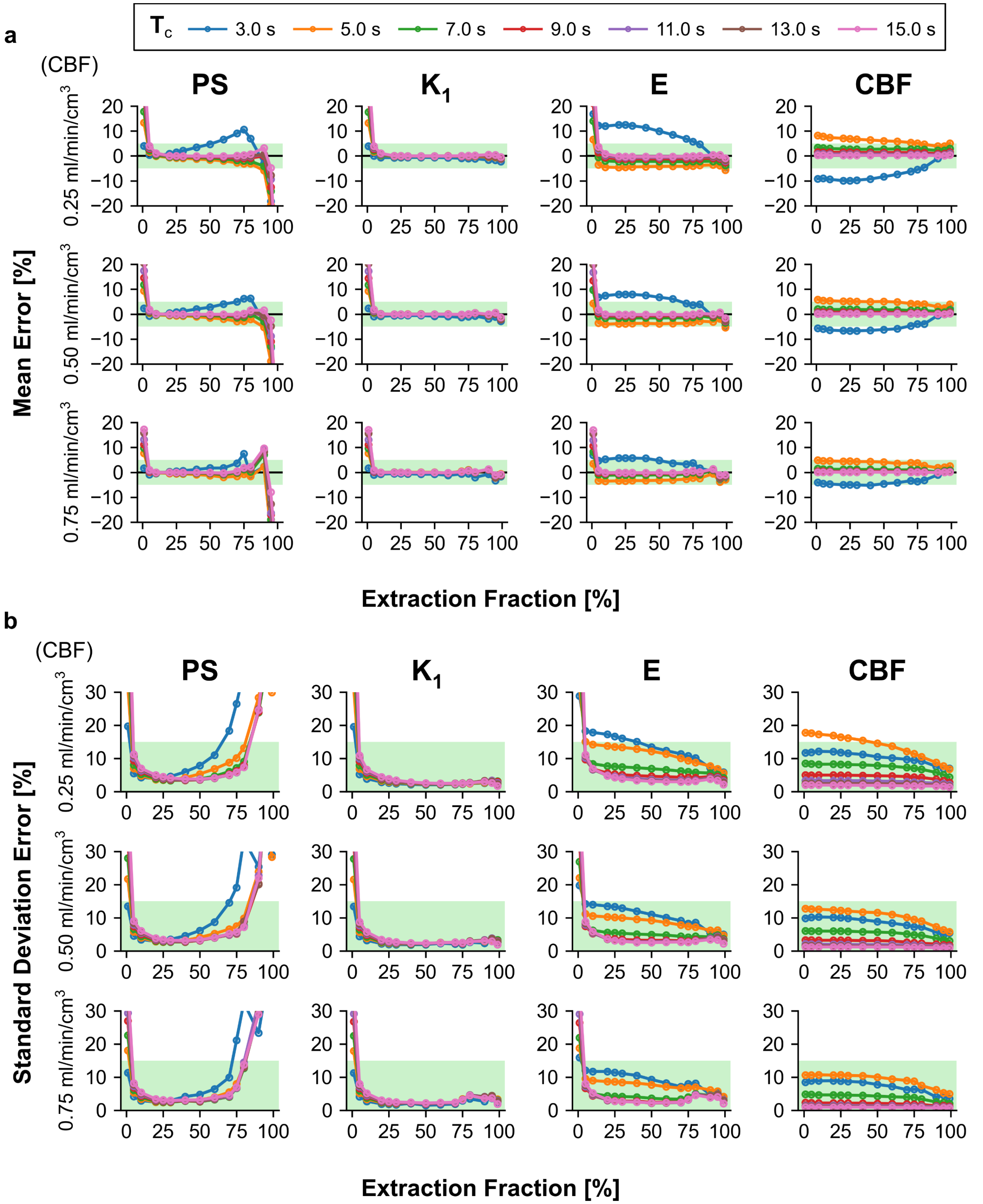

Supplementary Figure 1. Percent mean error (panel a) and standard deviation (panel b) of the blood-brain barrier (BBB) permeability-surface area (PS) product, BBB transport rate (K_1), extraction fraction (E), and cerebral blood flow (CBF) obtained from practical identifiability analysis

across a wide range of simulated extraction fractions (E, 0.01 to 0.99), mean vascular transit times (T_c, 3 to 15 s), and cerebral blood flows (CBF, 0.25 to 0.75 ml/min/cm^3). The green boxes indicate our thresholds for practical identifiability.

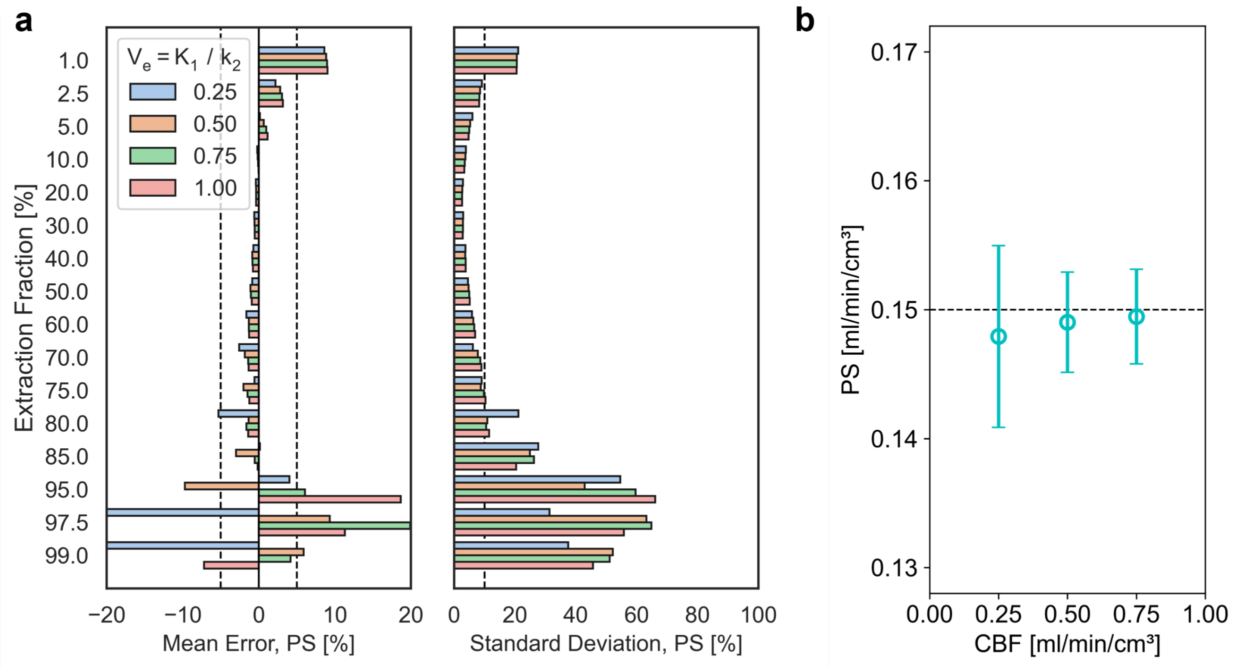

Supplementary Figure 2. Practical identifiability analysis of the permeability-surface area (PS) product. (a) The percent mean and standard deviation of errors for PS estimates when varying extraction fraction and extravascular distribution volume (V_e) where the dashed black lines indicate our identifiability thresholds. (b) Average and standard deviation of PS estimates in a simulation study fixing PS = 0.15 ml/min/cm^3 (dashed black line) and varying cerebral blood flow (CBF).

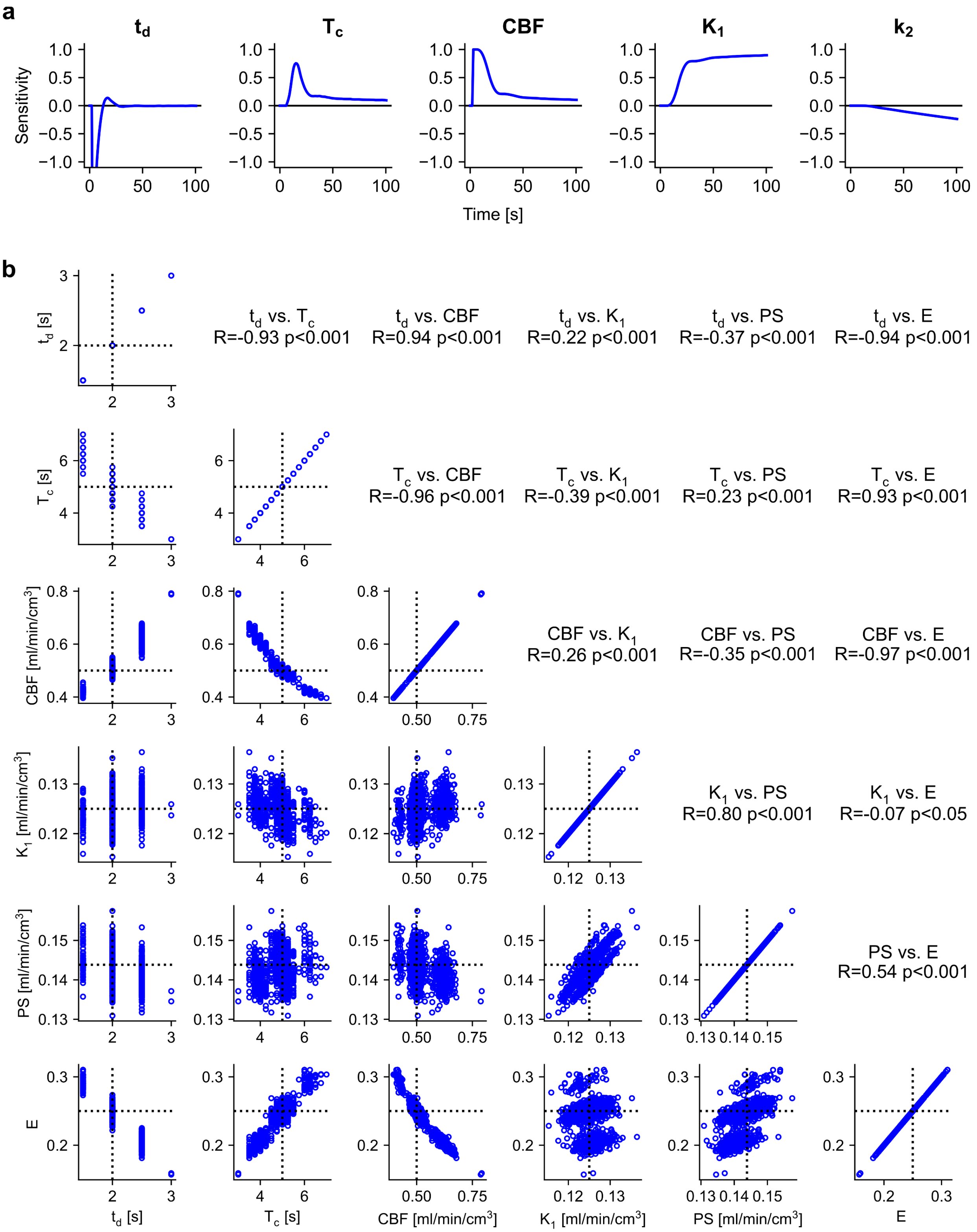

Supplementary Figure 3. Sensitivity analysis of the adiabatic approximation to the tissue homogeneity (AATH) model. (a) Normalized sensitivity functions of the five AATH model

parameters: time delay (t_d), mean vascular transit time (T_c), cerebral blood flow (CBF), blood-brain barrier (BBB) transport rate (K_1), and BBB clearance rate (k_2). The BBB permeability-surface area (PS) product and extraction fraction (E) were calculated from CBF and K_1 and their sensitivity functions had the same shape as that of K_1. (b) Scatter plots of parameter estimates across N=1024 noise realizations in Monte Carlo simulations for practical identifiability analysis. The dashed line indicates the simulated true model parameters. Each data point indicates the estimated value at a particular noise realization. The Pearson correlation coefficient (R) with two-tailed significance testing is indicated on the opposing diagonal. For both (a) and (b), t_d = 2 s, T_c = 5 s, CBF = 0.50 ml/min/cm^3, K_1 = 0.125 min/min/cm^3, PS = 0.144 ml/min/cm^3, E = 0.25, k_2 = 0.25 min^{-1} similar to that of ^{18}F-fluorodeoxyglucose (FDG).

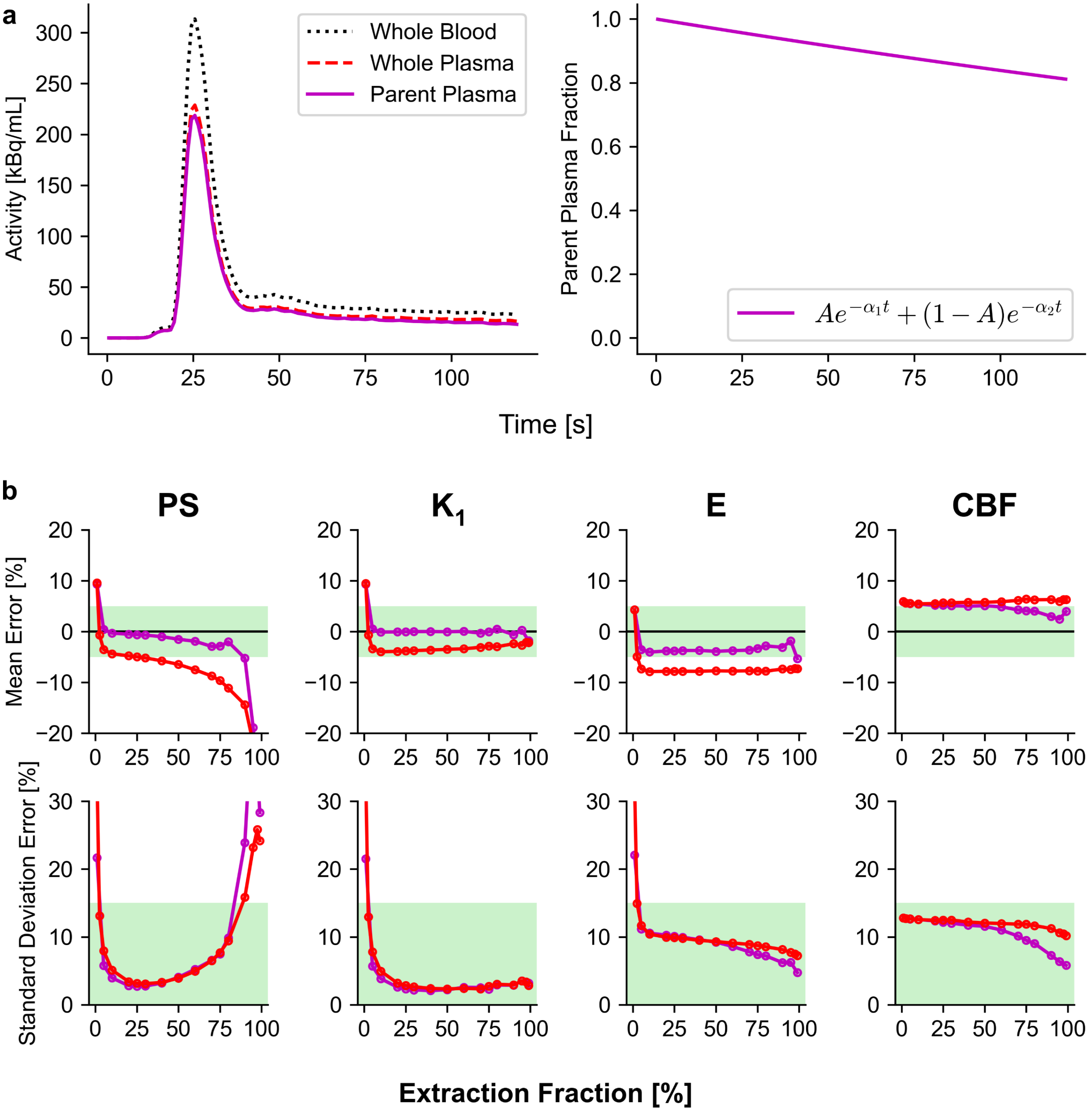

Supplementary Figure 4. Effect of radiometabolites on the practical identifiability of the blood-brain barrier (BBB) permeability-surface area (PS) product, BBB transport rate (K_1), extraction fraction (E), and cerebral blood flow (CBF). (a) A whole-blood arterial input function derived from the average of image-derived input functions from our cohort, with whole and parent arterial plasma curves simulated using a population-based parent plasma fraction of ^{18}F-florbetaben.[54] (b) Practical identifiability analysis when simulating tissue time-activity curves with the parent plasma curve but using the whole plasma curve (red line; i.e., neglecting plasma radiometaboiltes) or the parent plasma curve (purple line) for parameter estimation. For this experiment, we fixed

CBF = 0.50 ml/min/cm^3 and T_c = 5 s. The green boxes indicate our thresholds for practical identifiability.

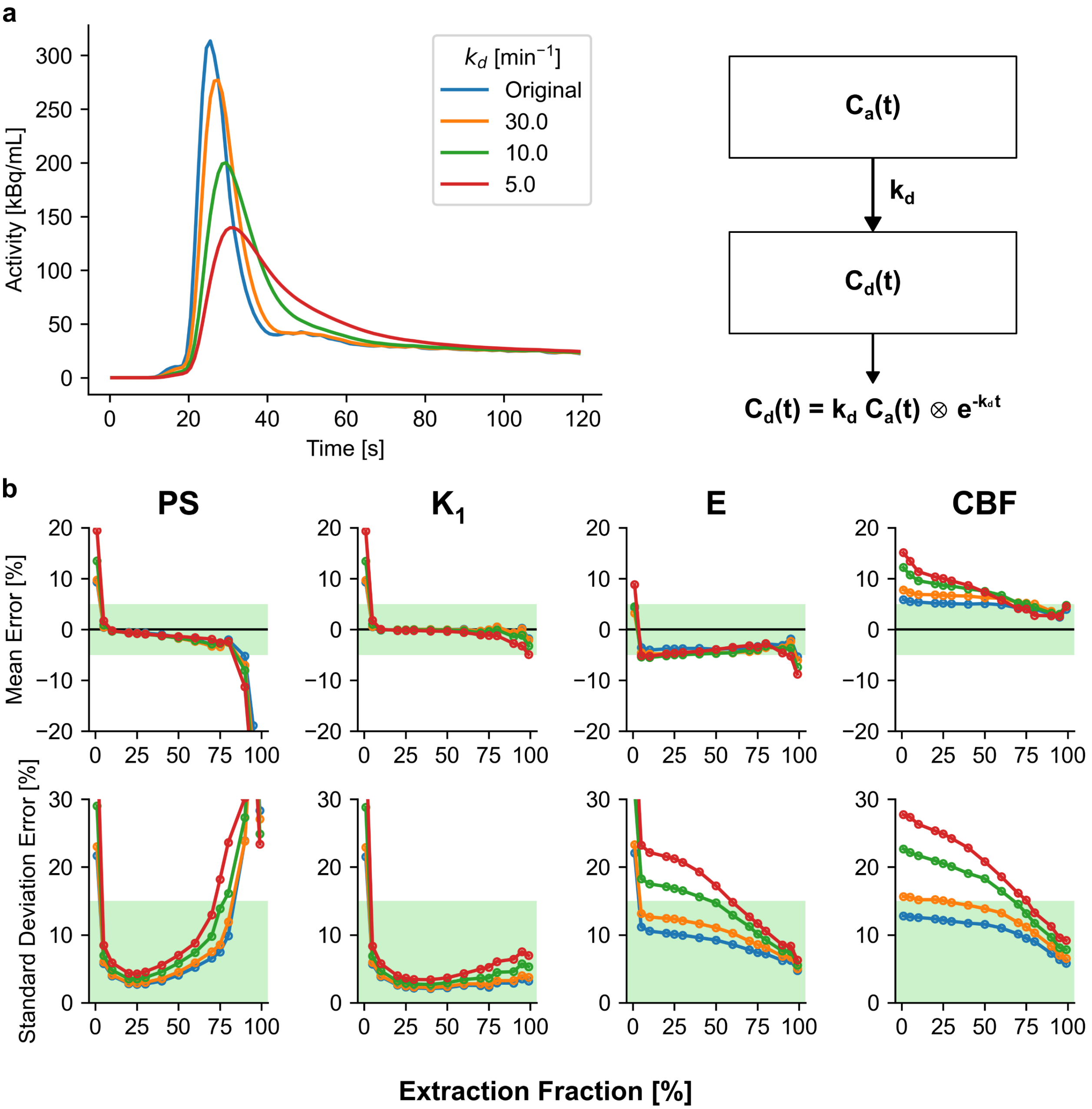

Supplementary Figure 5. Effect of the arterial input function shape on the practical identifiability of the blood-brain barrier (BBB) permeability-surface area (PS) product, BBB transport rate (K_1), extraction fraction (E), and cerebral blood flow (CBF). (a) We generated dispersed versions of a cohort-averaged image-derived arterial input function using a mono-exponential dispersion function, simulating three slower injection protocols. (b) Practical identifiability analysis for each dispersion level. For this experiment, we fixed CBF = 0.50 ml/min/cm^3 and T_c = 5 s. The green boxes indicate our thresholds for practical identifiability.

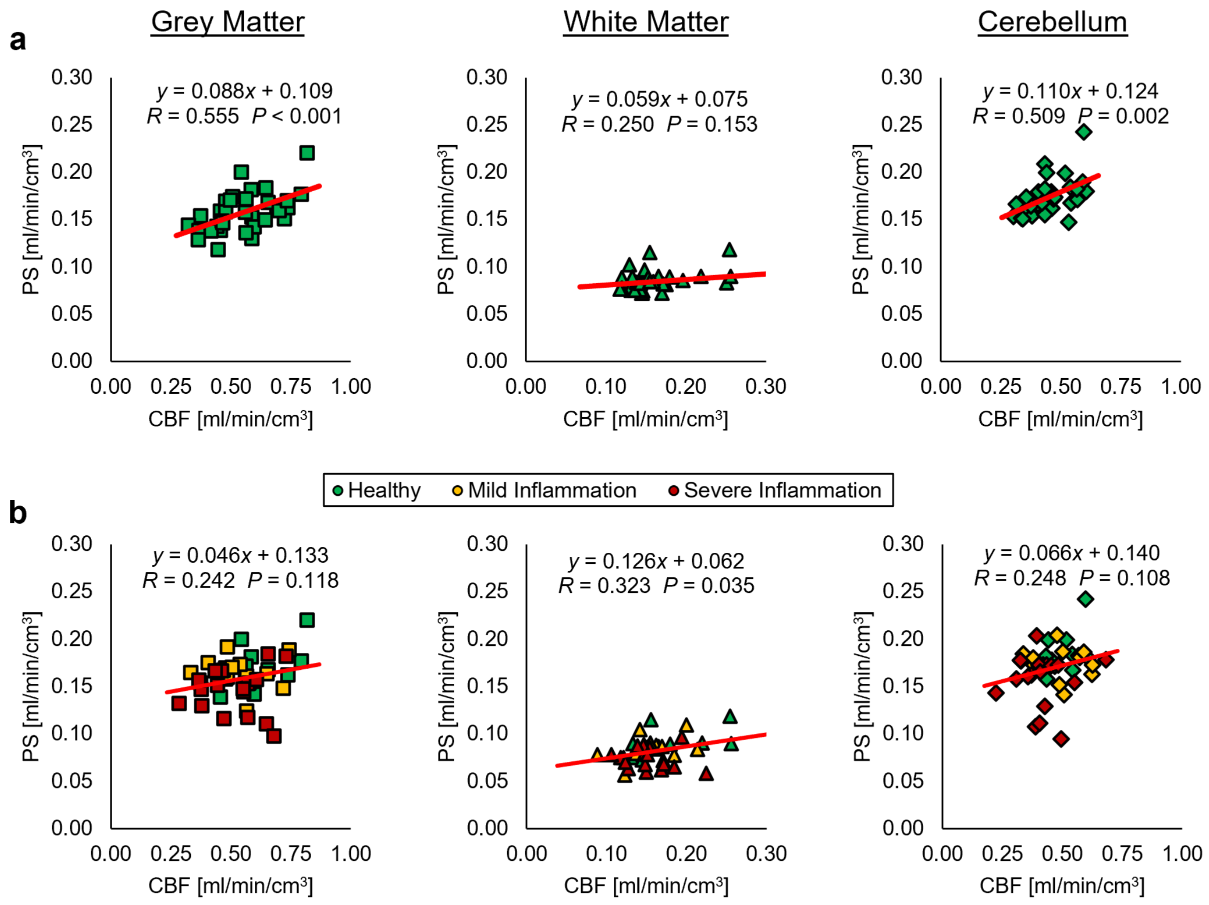

Supplementary Figure 6. Regional cerebral blood flow (CBF) versus the blood-brain barrier (BBB) permeability-surface area (PS) product of ^{18}F-fluorodeoxyglucose (FDG) in (a) 34 healthy subjects and (b) age-matched healthy controls (N=13) and patients with metabolic dysfunction-associated liver disease (MASLD)-related mild liver inflammation (N=13) and severe inflammation (N=17). Data are stratified by grey matter, white matter, and cerebellum. Pearson correlation coefficients (R) were computed with two-tailed significance testing.

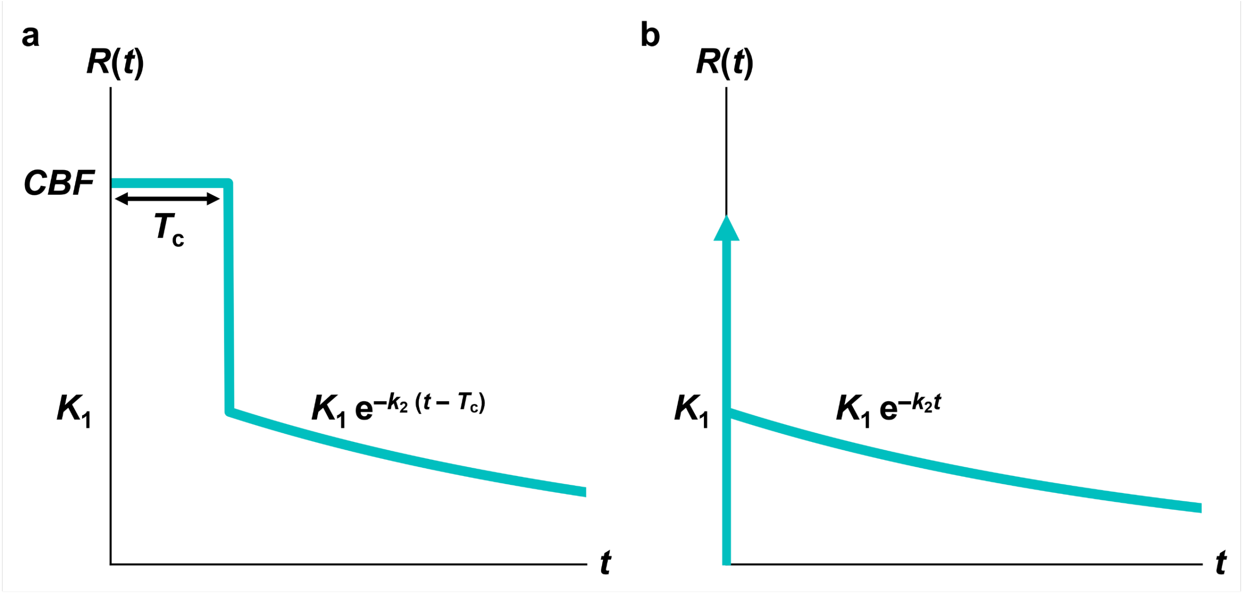

Supplementary Figure 7. Impulse response functions (R(t)) of tracer kinetic models investigated in this study for modeling the early-dynamic PET data of a radiotracer. **a**, The adiabatic approximation to the tissue homogeneity (AATH) model. **b**, The standard one-tissue compartment model. The arrow represents a delta function with an area equal to the blood volume, v_b. CBF indicates cerebral blood flow; K_1, the blood-brain barrier (BBB) transport rate; k_2, BBB clearance rate; T_c, mean vascular transit time.
