# Supplementary material for: Quantitative PET imaging and modeling of molecular blood-brain barrier permeability
Source: Nat Commun. 2025 Mar 30;16:3076. doi: 10.1038/s41467-025-58356-7 (PMC11955546; doi:10.1038/s41467-025-58356-7)
Supplement: Supplementary file 2 — Reporting Summary [file 41467_2025_58356_MOESM2_ESM.pdf]

nature portfolio

Corresponding author(s): Guobao Wang

Last updated by author(s): Mar 1, 2025

Reporting Summary

Nature Portfolio wishes to improve the reproducibility of the work that we publish. This form provides structure for consistency and transparency in reporting. For further information on Nature Portfolio policies, see our Editorial Policies and the Editorial Policy Checklist.

Statistics

For all statistical analyses, confirm that the following items are present in the figure legend, table legend, main text, or Methods section.

n/a	Confirmed	
☐	☒	The exact sample size (*n*) for each experimental group/condition, given as a discrete number and unit of measurement
☐	☒	A statement on whether measurements were taken from distinct samples or whether the same sample was measured repeatedly
☐	☒	The statistical test(s) used AND whether they are one- or two-sided<br>*Only common tests should be described solely by name; describe more complex techniques in the Methods section.*
☐	☒	A description of all covariates tested
☐	☒	A description of any assumptions or corrections, such as tests of normality and adjustment for multiple comparisons
☐	☒	A full description of the statistical parameters including central tendency (e.g. means) or other basic estimates (e.g. regression coefficient) AND variation (e.g. standard deviation) or associated estimates of uncertainty (e.g. confidence intervals)
☐	☒	For null hypothesis testing, the test statistic (e.g. *F*, *t*, *r*) with confidence intervals, effect sizes, degrees of freedom and *P* value noted<br>*Give P values as exact values whenever suitable.*
☒	☐	For Bayesian analysis, information on the choice of priors and Markov chain Monte Carlo settings
☒	☐	For hierarchical and complex designs, identification of the appropriate level for tests and full reporting of outcomes
☐	☒	Estimates of effect sizes (e.g. Cohen's *d*, Pearson's *r*), indicating how they were calculated

Our web collection on statistics for biologists contains articles on many of the points above.

Software and code

Policy information about availability of computer code

Data collection	All the dynamic PET/CT data were acquired using a commercial clinical PET/CT scanner uEXPLORER with vendor-provided software for image reconstruction.
Data analysis	Data analyses were performed using MATLAB and/or Python. Statistical analyses were performed with IBM SPSS Statistics 29.<br><br>Python code for kinetic analysis, which utilizes standard Python libraries, is available on Github (https://github.com/kjch03/bbb-permeability-pet) and Zenodo (https://doi.org/10.5281/zenodo.14954958)

For manuscripts utilizing custom algorithms or software that are central to the research but not yet described in published literature, software must be made available to editors and reviewers. We strongly encourage code deposition in a community repository (e.g. GitHub). See the Nature Portfolio guidelines for submitting code & software for further information.

Data

Policy information about availability of data

All manuscripts must include a data availability statement. This statement should provide the following information, where applicable:

- Accession codes, unique identifiers, or web links for publicly available datasets
- A description of any restrictions on data availability
- For clinical datasets or third party data, please ensure that the statement adheres to our policy

All the data that are associated with this article are present in the paper or the Supplementary Materials. The raw data of human subjects used in this paper can be provided by G.W. pending scientific review and a completed material transfer agreement. Requests for data should be submitted to G.W. The data used are not publicly available as the information could compromise the participants' privacy.

Research involving human participants, their data, or biological material

Policy information about studies with human participants or human data. See also policy information about sex, gender (identity/presentation), and sexual orientation and race, ethnicity and racism.

Reporting on sex and gender	Sex of participants in the studies was reported when appropriate. In the healthy aging study, 21 of the 34 subjects are females. Sex was included in the data analysis as one of the demographic factors and was not associated with a significant finding.
Reporting on race, ethnicity, or other socially relevant groupings	No data of race, ethnicity or other socially relevant groupings were used in this work.
Population characteristics	The healthy subjects in the work had a mean age of 51±13 years (range: 26 to 78 years). For the study of metabolic-dysfunction-associated liver inflammation study, the mean age was 52.4±13.0 y, and 51.0±11.0 y in the mild lobular inflammation and severe lobular inflammation groups.
Recruitment	Data of human subjects were obtained from the healthy subjects and patients with metastatic dysfunction-associated liver inflammation were enrolled at UC Davis Medical Center with IRB approval. Detailed information can be found in the paper.
Ethics oversight	University of California Davis Institutional Review Board (IRB)

Note that full information on the approval of the study protocol must also be provided in the manuscript.

Field-specific reporting

Please select the one below that is the best fit for your research. If you are not sure, read the appropriate sections before making your selection.

☒ Life sciences ☐ Behavioural & social sciences ☐ Ecological, evolutionary & environmental sciences

For a reference copy of the document with all sections, see nature.com/documents/nr-reporting-summary-flat.pdf

Life sciences study design

All studies must disclose on these points even when the disclosure is negative.

Sample size	No sample-size calculation was performed in this observational study. However, statistical tests were performed as appropriate to justify the statistical significance.
Data exclusions	No particular data were excluded. Groups were either based on all available data or formed based on a criterion as described in the paper (such as for age-group or age-match)
Replication	We used Monte Carlo simulation to calculate the uncertainty (bias and standard deviation) of the kinetic parameters to examine the practical identifiability of parametric PET measures.
Randomization	No randomization was used in this observational study. The focus of this paper is on the technical development.
Blinding	The investigators who did the data analysis were blinded to group allocation during data collection but were not blinded during data analysis. This observational study was aimed to demonstrate the potential of the proposed technical method, not trying to make a clinical or biological conclusion.

Reporting for specific materials, systems and methods

We require information from authors about some types of materials, experimental systems and methods used in many studies. Here, indicate whether each material, system or method listed is relevant to your study. If you are not sure if a list item applies to your research, read the appropriate section before selecting a response.

Materials & experimental systems

n/a	Involved in the study
x	Antibodies
x	Eukaryotic cell lines
x	Palaeontology and archaeology
x	Animals and other organisms
	x Clinical data
x	Dual use research of concern
x	Plants

Methods

n/a	Involved in the study
x	ChIP-seq
x	Flow cytometry
x	MRI-based neuroimaging

Clinical data

Policy information about clinical studies

All manuscripts should comply with the ICMJEguidelines for publication of clinical research and a completedCONSORT checklist must be included with all submissions.

Clinical trial registration	There are no direct clinical trials for this study. All data were retrospectively obtained from existing studies without added interventions for this study.
Study protocol	The detail of the protocol is described in the paper. A similar protocol for the patients with liver disease was also described in part in our published papers (PMC10096050).
Data collection	The data for the patients with liver disease were from 30 consecutive patients receiving liver biopsy between July 2020 and February 2023, while healthy subjects were from an existing study that consists of 34 healthy subjects.
Outcomes	The primary outcome of the proposed work is to demonstrate the usefulness of the proposed method for BBB permeability imaging. This is assessed by comparing different tracers, different age groups, and disease status (healthy vs liver disease). The secondary outcome is to evaluate the effect of age and blood glucose by regressing the PET measures against these variables.

Plants

Seed stocks	*Report on the source of all seed stocks or other plant material used. If applicable, state the seed stock centre and catalogue number. If plant specimens were collected from the field, describe the collection location, date and sampling procedures.*
Novel plant genotypes	*Describe the methods by which all novel plant genotypes were produced. This includes those generated by transgenic approaches, gene editing, chemical/radiation-based mutagenesis and hybridization. For transgenic lines, describe the transformation method, the number of independent lines analyzed and the generation upon which experiments were performed. For gene-edited lines, describe the editor used, the endogenous sequence targeted for editing, the targeting guide RNA sequence (if applicable) and how the editor was applied.*
Authentication	*Describe any authentication procedures for each seed stock used or novel genotype generated. Describe any experiments used to assess the effect of a mutation and, where applicable, how potential secondary effects (e.g. second site T-DNA insertions, mosiacism, off-target gene editing) were examined.*
