# Supplementary material for: Quantitative PET imaging and modeling of molecular blood-brain barrier permeability
Source: Nat Commun. 2025 Mar 30;16:3076. doi: 10.1038/s41467-025-58356-7 (PMC11955546; doi:10.1038/s41467-025-58356-7)
Supplement: Supplementary file 3 — Transparent Peer Review file [file 41467_2025_58356_MOESM3_ESM.pdf]

nature portfolio

Peer Review File

Quantitative PET imaging and modeling of molecular blood-brain barrier permeability

Corresponding Author: Dr Guobao Wang

This file contains all reviewer reports in order by version, followed by all author rebuttals in order by version.

Version 0:

Reviewer comments:

Reviewer #1

(Remarks to the Author)
The paper describes an extensive modelling study of high time resolution Total Body PET to estimate CBF as well as K1 (E*CBF where E=(1-exp(-PS/CBF)) from the scans and thus allow estimation of the tracer specific BBB permeability surface area, PS as well. The authors say that the PS of a tracer is related to different methods of transport across the BBB and that it can give more information about the integrity and function of the BBB. The method interrogates the function of the BBB in more depth than the usual DCE MRI scan where the BBB structural integrity is tested by looking for the small and slow leakage of contrast. The study uses the first two minutes of a scan from three separate tracers and shows that it is only at the high temporal resolution that this becomes possible, and that a model which can separate these parameters is preferable. This is a very well written paper which was enjoyable to read and is also a great and novel use of the technological development that is very high sensitivity Total Body PET, making it a very timely article.

One challenge of the presented study - as identified by the authors - is the difficulty in validating the measurement of PS against ex vivo work or other techniques, what is the gold standard for PS? How do we know we are measuring it? Some of my comments/questions below refer to this idea and how it could be given more of a chance.

Introduction:
1. Could be worth expanding on why DCE MRI doesn't provide great measurements of BBB leakage and PS anyway (eg. Drift and high SNR).
2. There should be more of a description about the different processes that the PS from each tracer is referring to and which diseases this might be applicable in or associated with - why is it useful to obtain a measurement of the PS for different tracers? Does it depend on the tracer if it is useful?

Model and method:
3. I don't quite understand the fitting approach - when you say you do a naïve grid search, is this to find the appropriate basis function spread? It was not clear if you do the NNLLS estimation of K1 and F for each step of the grid search? Or you do some kind of optimisation first to find the tc and Td and then fit for K1 and CBF? Please clarify the approach - add a figure in the supplementary information if it could be useful to show the process and the comparison.
4. I would very much like to see a sensitivity analysis of these parameters (K1/PS and CBF) included and the correlation matrices between parameters reported. The AIC demonstration, the error on the parameter estimates and the simulation study as well as the similarity of the CBF maps but not the PS between radiotracers all go some way to showing the method is able to separate parameters. Having said this, it would add a robustness to the method presented if you could show that each of the parameters can be uniquely estimated using a sensitivity analysis if it is possible. The question is: even if the model is the most appropriate and the values are estimated, has the system been sufficiently perturbed to well estimate and separate both the CBF AND the K1/PS? A short discussion is here on pg 516:
https://tauruspet.med.yale.edu/staff/edm42/chapters/kinetic-modeling-chapter-23-wernick-book.pdf
5. Were any other models considered for the CBF component (other than the AATH)?

Simulation:
6. Did you use the same model for simulation and estimation? It appears that it was the same model for both the simulation and the estimation with the addition of noise to the TACs.

Results:
7. What is the regional variation of the PS in the different tracers, particularly butanol?
8. Can you report the td and TC? Does the td or TC show any resemblance to literature values of arterial transit time as measured by ASL or other imaging methods?
9. Do the CBF measurements show resemblance to oxygen/water PET CBF or even ASL? There are some WM and GM values in literature that could be compared to your values.
10. Where are the regional results presented? If not yet in the paper, please add them to the supplementary information.
11. The 18FDG PS and CBF look somewhat similar - how do the regional spreads compare to one another? Are they correlated?
12. Can the authors comment on whether the ability to separate the K1 and the CBF would change depending on the tracer?
13. For some kind of validation, could you compare to the PS for each tracer to the relevant regional mRNA distribution for the appropriate transporter from the Allen Human Brain atlas? mRNA doesn't always lead to expression but it CAN and there may be several markers per transporter available to look at.

Discussion:
14. You mention the PS of gadolinium in the intro and that it increases in BBB breakdown & therefore increases the permeability measured. Would the same kind of leakiness seen in structural differences be also picked up in the PS of a PET tracer? What I mean is, that if there is a glioma or a stroke, or an MS lesion which changes the structural integrity of the BBB, I imagine it would let the small molecules pass through as it does the large gadolinium molecules, but would that also confound any changes brought to the molecular permeability changes? Would those two kinds of permeability be lumped in together? Thus you would see an increase due to leakiness from structural changes but then a decrease due to the molecular permeability and active transport mechanisms? Would it be possible to separate this? Can you please add a comment about what you would expect in this kind of situation?

15. Relatedly, I found it interesting that with the aging population you saw decreased PS and decreased CBF. Again, as above, I am surprised that there is no BBB structural breakdown showing an increase in PS - would you not expect this in an aging population? Perhaps the transporter is becoming less effective at transporting molecules, and therefore it decreases. A comment on this sort of confounding interaction in the discussion would be appreciated and it would be very interesting to test it in a case where there is some structural integrity change, and compare that to the DCE MRI for example.

16. Can you discuss a way you could challenge the PS (or the CBF!) for different tracers and see if the difference can still be well separated? Could you potentially also do a DCE MRI to see how much of a PS is disturbed by the structural changes and how much is functional due to the molecular transport across the BBB? Or compare the CBF to an oxygen PET or a ASL-MRI study (or another MRI measure of CBF)?

Reviewer #2

(Remarks to the Author)
This work presents an interesting modeling approach that leverages the high sensitivity of new long-axial full body scanners to estimate cerebral blood flow and tracer transport rate into brain tissue (K1) from the same (single) scan dynamic data and thereby calculate an estimate of permeability*surface area product to infer BBB permeability from a single scan. This is an interesting method; however, more work is needed to establish the validity of this approach. The novelty of this method from a modeling perspective is the proposed estimation of CBF (and TC (mean vascular transit time)) from high temporal resolution data acquired during the first pass. The results must clearly demonstrate accurate estimation of CBF and separate identifiability of CBF from both E and TC. The challenge in parameter identifiability of CBF and E becomes evident if CBF*E is substituted for K1 in Eq. 4 (it is suggested to present the equation this way); with the current formulation that couples CBF and E estimation its extremely challenging to establish estimation of E independently of CBF. Several practical items are also unaddressed in discussion regarding extension of this potential method beyond the presented data.

The identifiability of CBF separately from T_C needs to be established through simulations, particularly for radiotracers with higher extraction fractions.

The simulation results examining ‘practical identifiability of CBF and E’ only tell the reader about accuracy and variance in E estimation for a single CBF value. The simulations should be expanded to a broader space of reasonably expected CBF values. Results addressing the identifiability of CBF from E must be shown. It is difficult to interpret Figures 4 and 5 without first demonstrating that CBF and E estimates with this model do not covary. A missing but critical piece is a demonstration that consistent PS estimates are made in settings where CBF is experimentally manipulated. While experimental data would be most convincing, simulations could help address this point.

The reported modeling results are quite sparse. Estimated values of T_C and T_d are not reported, but these are quite relevant to the successful implementation of this model and need to be added. Do the estimated t_C values make sense relative to what would be expected biologically?

An important consideration is the potential role for radiolabeled metabolites on this method when using the ascending aorta to estimate the arterial input function. All of the presented radiotracers ([18F]fluciclovine, [18F]FDG, [11C]Butanol) do not have significant radiolabeled metabolites, so this is not particularly relevant to the presented data. However, it is highly relevant for the host of radiotracers for brain targets mentioned in discussion lines 289-292, and this limitation for extension

of this model to other radiotracers requires consideration.

Please state in Methods the duration of the bolus injection of radiotracer for these studies.

Reviewer #3

(Remarks to the Author)
Title: Quantitative PET imaging and modeling of molecular blood-brain-barrier permeability

General
This is an interesting piece of research using new kinetic modeling approaches to study blood-brain barrier permeability enabled by the high sensitivity of LAFOV pet/ct technology. The paper is very clearly written and the findings are of high interest to the scientific community. The limitations are acknowledged and well discussed. Yet, I have some comments that should be clarified.

Minor comments
- Some references are self-centered. It may be good to refer to other LAFOV PET/CT systems as well as they also have ultra-high sensitivity
- Some references to 15O-water pet may be good to mention. Radiowater is still considered to be the reference tracer for perfusion imaging.
- Some more details on the placement and size of the image derived input functions would be nice
- Butanol and Fluciclovine show tracer metabolism. Are the input functions corrected for plasma to blood ratio and metabolites. If so, how. If not, please discuss implications for the models, it may affect k2 estimates? I understand that it may be a minor issue for the first 2 mins of the scan.

Major comments
- The AATH model ignores tissue fraction, as opposed to the standard single tissue model (using 1-Vb). The implication of not considering the tissue fraction should be discussed
- Should future work aim to merge both modelling approaches?
- Please add assessment of CBF grey/white matter ratio's for all 3 tracers to see if regional CBF values are comparable using the various models for the 3 tracers…they should if the estimate of extractions or PS is accurate. We now can compare the reported CBF only with some population values.
- A parametric as well as regional comparison of K1 estimated using AATH and the conventional kinetic model per patient for each tracer would be nice. In particular for butanol with a near 100% extraction the data points should be the same and align along the line of identity. If not, please discuss the reason for it (in case of butanol, for the other tracers it should align with the average extraction fraction)
- How do you deal with delay and dispersion, in particular for the AATH model. I recommend to add some simulations with 0.25,0.5,1.0 and 2.0 s errors in the delay estimation (in both directions) and how that would affect the observed E (or PS) and CBF values.
- Are 2 min scan duration optimal for both models? Some data on shorter or longer scan durations would be nice. Why choose 2 min.
- Does the administration protocol matter…so the speed of administration. For sure you would need a fast bolus administration for the AATH model, but how would the model respond if the administration was a bit dispersed due to issues in the administration line.
- What were the radtiotracer administration settings (speed, volume, etc). Also here it would be good to have some simulations or orther data to get idea on how critical this is.

Reviewer #4

(Remarks to the Author)
This manuscript details the methodological framework for in vivo measurement of the blood-brain barrier transport using high temporal resolution PET imaging in humans. Measurements of permeability-surface area (PS) product are made across three radiotracers and demographic/clinical conditions of aging and MASH. A distributed model was applied for parameter estimation demonstrating the uncoupling of cerebral blood flow and PS measures as well as variability in PS across conditions. The ability to measure BBB permeability via the PS metric is a highly significant methodological advancement in neuroimaging over existing methods (e.g. DCE-MRI), making a potentially profound impact on advancing the basic understanding of BBB physiology as well as translation into clinical care. Further, it demonstrates the scientific utility of the emerging technology of total body PET imaging systems which afford the opportunity of rapid temporal imaging due to increased field-of-view and high detection sensitivity acquisitions. The manuscript is well written providing detailed descriptions of the experiments performed to carefully examine BBB permeability across conditions and results that support the tested hypothesis. Because a somewhat nonstandard analytic method for dynamic PET data was used, additional details and background are warranted to assuage the concerns with a lack of parameter identifiability in the measured outcomes. The presentation of the findings and clarity of the manuscript would be greatly improved by addressing the

following concerns:

AATF model is nonstandard compared to compartment models) for dynamic PET time-series data and additional details equating it to S1TC and S2TC would aid the reader with interpreting these outcomes without having to closely study the Lawrence & Lee (1998) paper. Specifically:
- Distinguishing CBF from K1 in equation 2 is confusing, particularly with referring to K1 as the BBB transport rate. The relationship of K1 = CBF*E should be defined here to aid in the interpretation of this parameter. Further, the parameter "F" is mentioned on this page and supplementary figure 2, but not defined.

- Please provide a more clear definition of the vascular transit time "Tc" as it appears to be the distinguishing feature between the AATH and S1TC models. Specifically if it references the tissue capillary transit time to denote a specificity to the region of perfused tissue as separate from the vascular space between aortic sampling and (through the) tissue of interest.

- Equation 4, please check to limits of integration. For the CBF term, it would seem the first term would only be integrated from 0->Tc (assuming a delay corrected Ca) and the K1 term from Tc -> t.

- Given the necessity for rapid sampling of the tissue, it would appear that some type dispersion in Ca (eq 3) should be included in the model (Hutchins 1986; Meyer 1989) to account for differences between aortic and regional tissue vasculature for examining the effects on CBF and K1 estimates.

- Coupled with the above issue is the "sharpness" of the bolus injection. A short, rapid bolus injection is typically needed to uncouple K1 and k2 using the 1TC model. Is this also required for the estimation of CBF and K1 with the AATH model? Would equation 2) still apply for a long Tc with respect to rapid extravascular clearance? There are no details regarding the injection of any of the radiotracers, e.g. 5- or 30-sec infusion. Was it closely monitored for all of the studies. Details regarding the shape of AIF bolus with CBF and K1 identifiability should be presented.

- please define the 5 parameters that are estimated from equation 4, assuming that time delay correction has already been applied. Although the noise simulation methods for determining parameter identifiability are sound and figure 1S compelling, there remains a concern of high covariance between many of the estimated parameters, particularly with td and Tc. It would be helpful to include some commentary on the limitations of accurate parameter identifiability.

- the assumption is made that no radiolabeled metabolites are present, it would be helpful to know if their presence is negligible on the measured outcomes. Similarly, this could also confirm that varying radiochemical purity (frequently between 90%-100%) of the injected PET drug product can be neglected.

- a limitation should also be included noting the lack of test/retest variability in the PS and CBF estimates, including paradigms that alter CBF (e.g. hypercapnic conditions).

Other Issues:
Intro, p.3, para2 – PET studies with butanol have also been conducted with [O-15]butanol, which would seem to be a better option for this application. It should at least be mentioned that [O-15]butanol would also be an option for these measurements.
Intro, p.3, last sentence – the ability of TB PET to measure image-based AIFs may be oversold a bit, given the need for external metabolite correction for most radiotracers.
p.12, para 1, please provide the effective spatial resolution of the reconstructed images used for this investigation, assuming the selected voxel size (2.344-mm) would not reproduce NEMA-based measures.
p.12, para 2 – knowing that many millions of radiological imaging scans are conducted yearly using ionizing radiation to provide invaluable clinical information, it is not evident why the involvement of radiation exposure (at safe levels) is a limitation. The dosimetry details here and throughout the Methods section seem misaligned to the overall theme of this manuscript focusing on methodological development.
p.15, para 3, the first sentence ends with stating that S1TC can only estimate K1, which leads to the following sentence stating that PS can be derived from K1. Presumably this applies only to the AATH model. If so, please clarify that PS was not estimated from the S1TC K1.

p.15, last paragraph, one of the often stated advantages of TB PET is the potential for injected dose reduction, yet these experiments appear to give standard clinical amounts. It's understandable that high counting statistics should be sought for this methodology development, however, it would be helpful if the authors could comment on the accuracy of the AIF and outcome measures for a reduced dose study.
Figure 1 – this is a very nicely crafted and informative illustration of the concepts being investigated in this study. One suggestion would be to explicitly state in the legend or body text that leakage as visualized by junction openings in a) can also be present in b) - d) but can be neglected due to the relative differences in scale of PS product.
Figure 2b –illustration of AIF with varying temporal sampling. Although it could be obscured by the time-axis scaling, it does not appear that the integrals (i.e. AUC) for the 1-, 5-, 10-sec curves are equivalent as would be expected from total counts divided by frame duration of each point. Can an explanation be given for this?
Figure 3b – the AATH model estimates CBF and K1, and presumably the E (=K1/CBF) and PS images are calculated by equation 7. This isn't evident from the figures for fluciclovine and FDG. The extraction image (E) does, to some degree, reveal enhanced signal consistent with K1. However, lower signal in E does not appear to track with the high signal in CBF (particularly in fluciclovine). Is this an artifact of colorbar scaling?

Figure 6b – please give consideration to the precision of the estimated Vmax and Km listed on the figure and the text on p.8, para 3. Given the high variability shown in the figure, it would seem this precision in the estimates is excessive.

Version 1:

Reviewer comments:

Reviewer #1

(Remarks to the Author)
Review of rebuttal: Quantitative PET imaging and modeling of molecular blood-brain barrier permeability

The extra results generated by the authors, and the modifications to the manuscript have satisfied my queries. No more changes required, thank you.

(Remarks on code availability)

Reviewer #2

(Remarks to the Author)
The revisions contain an impressive amount of work in support of their model.
While I remain uncomfortable with a model that relies so heavily on estimating a key parameter mostly based on 2-8 data points, there is enough evidence to support it being reasonably robust for the tested scenarios.

Two minor comments:

Equation (4): for consistency with Equation (7), shouldnt C_wb in the CBF terms and C_p in the K_1 term really be C_a ?

In R.1.11., The correlation illustrated in Supplementary Figure 6 could indicate parameter coupling. In the authors response, they suggested that these correlations may become decoupled in a disease state, but their reference to Figure 5 doesnt necessarily support that, so an additional correlation plot/analysis would be very useful in support of that point.

(Remarks on code availability)

Reviewer #3

(Remarks to the Author)
The authors substantially revised the manuscript and addressed all my comments (except one, but I accept that this may be part of future work). Additional analysis were performed and added to the manuscript (or supplemental material).

I think it is a very interesting approach, very well explored and evaluated.

Based on the response to my comments and the other 3 reviewers, I have no further major comments and one minor comment

The authors prefer to report CBF per unit voxel volume (rather than tissue volume) based on the average5% blood volume fraction they indicate that this is a minor difference. I partly agree in case of grey matter tissue, but only regions that are not intersected with a larger blood vessel. So, i do think the authors should clarify in the manuscript that K1 (or CBF) is expressed per voxel volume and not per tissue volume (as would be expected when reporting CBF). It is fine to mention that the expected difference is 5% assuming average blood volume fraction of 5%.

(Remarks on code availability)

Reviewer #4

(Remarks to the Author)
The concerns of this reviewer have been thoughtfully and thoroughly addressed.

(Remarks on code availability)

Open Access This Peer Review File is licensed under a Creative Commons Attribution 4.0 International License, which permits use, sharing, adaptation, distribution and reproduction in any medium or format, as long as you give appropriate credit to the original author(s) and the source, provide a link to the Creative Commons license, and indicate if changes were made.
In cases where reviewers are anonymous, credit should be given to 'Anonymous Referee' and the source.
The images or other third party material in this Peer Review File are included in the article's Creative Commons license, unless indicated otherwise in a credit line to the material. If material is not included in the article's Creative Commons license and your intended use is not permitted by statutory regulation or exceeds the permitted use, you will need to obtain permission directly from the copyright holder.
To view a copy of this license, visit https://creativecommons.org/licenses/by/4.0/

Response to Review Comments

We would like to thank the reviewers and editors for their thoughtful consideration of our manuscript. The reviewers raised important questions about the reliability of our methodology as well as helpful suggestions to broaden the breadth of our work. We have conducted additional experiments and made substantial changes in response to these concerns, which we hope clarifies the validity and significance of our proposed approach. The reviewer comments are italicized and marked in dark teal. Relevant changes to the manuscript are in red text.

Reviewer #1 (Remarks to the Author):

*The paper describes an extensive modelling study of high time resolution Total Body PET to estimate CBF as well as K1 (E*CBF where E=(1-exp(-PS/CBF)) from the scans and thus allow estimation of the tracer specific BBB permeability surface area, PS as well. The authors say that the PS of a tracer is related to different methods of transport across the BBB and that it can give more information about the integrity and function of the BBB. The method interrogates the function of the BBB in more depth than the usual DCE MRI scan where the BBB structural integrity is tested by looking for the small and slow leakage of contrast. The study uses the first two minutes of a scan from three separate tracers and shows that it is only at the high temporal resolution that this becomes possible, and that a model which can separate these parameters is preferable. This is a very well written paper which was enjoyable to read and is also a great and novel use of the technological development that is very high sensitivity Total Body PET, making it a very timely article.*

One challenge of the presented study - as identified by the authors - is the difficulty in validating the measurement of PS against ex vivo work or other techniques, what is the gold standard for PS? How do we know we are measuring it? Some of my comments/questions below refer to this idea and how it could be given more of a chance.

Thank you for taking time to review this paper and providing constructive feedback. As pointed out, validating the accuracy of our PS measurement was a central challenge, and the reviewer offered many helpful comments to interpret our method and results in this regard. Here, we have elaborated on the interpretation of our method and results with experiments to support the validity of our approach. Point-by-point response to each comment is provided below.

Introduction:

R1.1. *Could be worth expanding on why DCE MRI doesn't provide great measurements of BBB leakage and PS anyway (e.g. Drift and high SNR).*

Thanks for the suggestion. Here we would like to highlight conceptual differences and avoid technical criticisms of the DCE-MRI method. However, we agree it is also beneficial to briefly mention the technical limitations of DCE-MRI. Thus, we have slightly modified the text in the Introduction (page 3):

… for gadolinium MRI contrast agents.[8] Despite certain technical challenges (e.g., due to signal drift and low signal-to-noise ratio), DCE-MRI measures of BBB permeability …

R1.2. *There should be more of a description about the different processes that the PS from each tracer is referring to and which diseases this might be applicable in or associated with - why is it useful to obtain a measurement of the PS for different tracers? Does it depend on the tracer if it is useful?*

As illustrated in Figure 1, BBB PS may differ between radiotracers and may reflect the function of different BBB transport mechanisms. All BBB PS measurements may be informative about the BBB permeability of a

particular molecule and about the basic physiology of the BBB. Furthermore, each radiotracer has its own existing clinical application and adding tracer-specific PS may enable multiparametric imaging to simultaneously assess different pathophysiological features of the disease. This aspect and relevant clinical applications have been further elaborated in detail in the Discussion (page 13-14). We also added a few phrases to the Introduction to better clarify the concept and potential application (page 3):

> We hypothesize that measuring the BBB PS of PET molecular radiotracers may open new opportunities to probe the human BBB at the molecular level and advance our basic understanding of BBB physiology. … Thus, ^{18}F-FDG PET has the potential to assess both cellular metabolism and molecular BBB function, with applications in Alzheimer's disease to study glucose permeability and metabolism.[1,14] The use of other PET radiotracers may allow us to study the BBB permeability of that specific molecule in addition to its molecular target for multiparametric imaging.

Model and method:

R1.3. *I don't quite understand the fitting approach - when you say you do a naïve grid search, is this to find the appropriate basis function spread? It was not clear if you do the NNLLS estimation of K1 and F for each step of the grid search? Or you do some kind of optimisation first to find the tc and Td and then fit for K1 and CBF? Please clarify the approach - add a figure in the supplementary information if it could be useful to show the process and the comparison.*

All the model parameters are jointly estimated following a nonlinear least-square minimization problem. We clarified the fitting procedure in the Methods (page 19):

> We used the least-squared curve fitting formulation with a basis function algorithm to estimate the parameters of the time delay-corrected AATH model:
>
> $$\boldsymbol{\theta} = \underset{\boldsymbol{\theta}}{\operatorname{argmin}} \sum_{m=1}^{M} w_m \left(Q(t_m) - \hat{Q}(t_m)\right)^2 \quad (5)$$
>
> where $\boldsymbol{\theta} = [CBF, K_1, k_2, T_c, t_d]$ are the model parameters to be estimated, $Q(t)$ and $\hat{Q}(t)$ are the measured and fitted time-activity curves, respectively, M is the number of time frames, t_m is the midpoint time of the mth frame, and w_m is the residual weighting factor ($w_m = 1$ in this work). Specifically, we performed a naïve grid search of $t_d \in [0,16]$ s and $T_c \in [3,16]$ s at 0.25 s intervals and used 100 logarithmically spaced $k_2 \in [0.006, 3]$ min^{-1}, resulting in 344,500 combinations of t_d, T_c, and k_2. Substituting the grid-searched values of t_d, T_c, and k_2 into Equation 4 results in CBF and K_1 as the remaining two unknowns. CBF and K_1 are linear scaling factors and were estimated by a non-negative linear least squares algorithm,[97] which minimized the sum of squared deviation between the measured $Q(t)$ and the fitted AATH time-activity curve.

R1.4. *I would very much like to see a sensitivity analysis of these parameters (K1/PS and CBF) included and the correlation matrices between parameters reported. The AIC demonstration, the error on the parameter estimates and the simulation study as well as the similarity of the CBF maps but not the PS between radiotracers all go some way to showing the method is able to separate parameters. Having said this, it would add a robustness to the method presented if you could show that each of the parameters can be uniquely estimated using a sensitivity analysis if it is possible. The question is: even if the model is the most appropriate and the values are estimated, has the system been sufficiently perturbed to well estimate and separate both the CBF AND the K1/PS? A short discussion is here on pg 516: https://tauruspet.med.yale.edu/staff/edm42/chapters/kinetic-modeling-chapter-23-wernick-book.pdf*

We conducted sensitivity analysis of the AATH model parameters and reported the results in Supplementary Figure 3 and Supplementary Table 3. However, sensitivity analysis only offers a first-order local

approximation to the change in time-activity curve for a small change in a model parameter. A more rigorous approach is practical identifiability analysis that uses more time-consuming and computationally expensive Monte Carlo simulations to characterize the accuracy of parameter estimates in response to noise perturbation across a wide range of kinetic conditions. Hence in this revision, we also conducted additional practical identifiability experiments, expanding the explored parameter space, showing the accuracy of PS, K1, E, and CBF estimates across various simulated values of E (1 to 99%), Tc (3 to 15 s), and CBF (0.25, 0.50, 0.75 ml/min/cm^3). The detailed results are summarized in a new Results subsection "*AATH model parameters are identifiable across experimental conditions*" (pages 7-9). Part of the results were pasted below:

> Sensitivity analysis of the AATH model parameters suggested that CBF and K_1 could be independently estimated (Supplementary Figure 3; Supplementary Table 3). Errors in t_d and T_c estimations appeared strongly correlated with that of CBF and E, but only weakly with PS or K_1 (Supplementary Figure 3). Underestimating t_d led to an overestimation of PS and K_1, whereas this led to an underestimation of E and CBF. In contrast, underestimating T_c generally led to an underestimation of PS and K_1 and overestimation for E and CBF, though the trend was less predictable at high extraction fractions. Despite these correlations, our practical identifiability analysis showed that our parameters of interest had low bias (<5%) and standard deviation (<15%) across a wide range of kinetics.

R1.5. *Were any other models considered for the CBF component (other than the AATH)?*

We indeed considered other models but the AATH stood out for its relatively simple closed-form time-domain solution. We have added the following text below in the Discussion for clarification (page 14):

> Advanced kinetic models and … Here, we used the AATH model as it modeled both CBF and K_1 with a relatively simple closed-form time-domain solution.[46] Alternative approaches include the one-barrier distributed parameter model[18,40,44] and model-independent deconvolution.[24,45] Implementation and parameter estimation with the one-barrier distributed parameter is challenging, and model-independent deconvolution requires regularization,[24,45] possibly biasing parameter estimates. … In future work, we will optimize the protocols and compare the strengths and weaknesses of each method …

Simulation:

R1.6. *Did you use the same model for simulation and estimation? It appears that it was the same model for both the simulation and the estimation with the addition of noise to the TACs.*

Yes, the AATH model was used to simulate time-activity curves for practical identifiability analysis. This is the standard approach to assess practical identifiability.[1–3] The goal is to determine the accuracy of parameter estimates due to noise. Using the same model for simulation and estimation means that all fitting errors are attributed to statistical noise and not inaccuracies of the model to true physiology. This is unlikely to be completely true, but is a reasonable assumption to characterize noise-induced variability. This is also justified by the excellent fitting of the AATH model to the measured time-activity curves (Figure 2c).

Results:

R1.7. *What is the regional variation of the PS in the different tracers, particularly butanol?*

The standard deviation of PS was ≈0.003 and 0.010 ml/min/cm^3 for ^{18}F-fluciclovine and ^{18}F-FDG, respectively, and did not substantially differ between regions. More specific information for each region, radiotracer, and parameter can be found in the new Supplementary Table 1.

As described in the text (Results, *Molecular BBB PS Differs Across PET Radiotracers*), we did not report the PS values of ^{11}C-butanol because the median estimated extraction fraction was 1, and the logarithm in Equation 1 ($PS = -CBF \ln(1-E)$) cannot be evaluated when E = 1.

R1.8. *Can you report the td and TC? Does the td or TC show any resemblance to literature values of arterial transit time as measured by ASL or other imaging methods?*

Following the suggestion, Supplementary Table 1 was added and summarizes the average ± standard deviation of td, Tc, and other model parameters. Instead of comparing our Tc estimates against ASL arterial transit time, which conceptually is slightly different than our Tc parameter, we added a comparison against a dual-tracer PET estimate (Results subsection *High-temporal resolution dynamic PET enables single-scan imaging of CBF and tracer-specific BBB transport rate*) (page 6):

> Estimated mean vascular transit time T_c values ranged on average from 4 to 7 s for ^{18}F-fluciclovine and ^{18}F-FDG with longer vascular transit times in the white matter, agreeing with previous dual-tracer PET estimates.[25] For ^{11}C-butanol, our estimates of T_c and cerebral blood volume were greater than the expected physiological range (Supplementary Table 1). … Nonetheless, we consider T_c a method parameter and despite an overestimation of T_c, our estimates of CBF and K_1 using ^{11}C-butanol were within expected physiological ranges and quantitative values.

R1.9. *Do the CBF measurements show resemblance to oxygen/water PET CBF or even ASL? There are some WM and GM values in literature that could be compared to your values.*

Our CBF estimates with all three investigated radiotracers were within literature ranges derived from flow-tracer PET. We added a new paragraph to the Results subsection: *High-temporal resolution dynamic PET enables single-scan imaging of CBF and tracer-specific BBB transport rate* (page 5):

> The average estimated regional CBF across the fifteen included subjects was 0.476±0.100, 0.173±0.036, and 0.427±0.069 ml/min/cm^3 in the cortical grey matter, white matter, and cerebellum, respectively. These values were within the expected ranges of average regional CBF previously established with flow-tracer PET (grey matter: 0.44 to 0.83 ml/min/cm^3;[17–20,26,27] white matter: 0.16 to 0.32 ml/min/cm^3;[17–19,26,27] cerebellum: 0.41 to 0.56 ml/min/cm^3)[19,21,22]. There were no significant differences ($P>0.50$) in regional CBF across PET tracers (Supplementary Table 1), suggesting our method could estimate CBF consistently across radiotracers. …

R1.10. *Where are the regional results presented? If not yet in the paper, please add them to the supplementary information.*

Figure 3 reports PS, K_1, and CBF estimates of each radiotracer for grey matter, white matter, and cerebellum as indicated by the different coloured bars. The newly added Supplementary Table 1 also reports the kinetics for each radiotracer stratified by these three brain regions.

R1.11. *The 18FDG PS and CBF look somewhat similar - how do the regional spreads compare to one another? Are they correlated?*

A plot comparing regional (grey matter, white matter, cerebellum) CBF versus regional FDG BBB PS across our 34 healthy subject cohort has been added as Supplementary Figure 6. Regional CBF and FDG BBB

PS are indeed correlated (Pearson R=0.82, p<0.001; linear regression y=0.185x+0.067). We commented on this in Results subsection *BBB PS of ^{18}F-FDG Decreases in Healthy Aging* (page 9):

> … Regional CBF and FDG BBBS PS were correlated (R=0.82, P<0.001; Supplementary Figure 6), but differ in scale and each represents a distinct physiological feature.

Despite their correlation under healthy conditions, they may become decoupled, such as in our MASLD study showing that FDG BBB PS was significantly different between differing severities of liver inflammation but not CBF (Figure 5). Future work will study how CBF and BBB PS may differ in other disease states. This was further described in the Discussion (page 13):

> Multiparametric imaging of CBF, molecular BBB permeability … FDG-PET scan. Importantly, each parameter represents a distinct physiological feature and a dysfunction of one parameter may point to a specific pathophysiological mechanism, for example, in our MASLD study, where FDG BBB PS differed between severities of liver inflammation but not CBF (Figure 5). ...

R1.12. *Can the authors comment on whether the ability to separate the K1 and the CBF would change depending on the tracer?*

The ability to separate K1 and CBF does depend on the tracer. Practical identifiability analysis for the specific kinetics of each tracer (Supplementary Table 2) showed that the reliability of CBF and K1 estimations were overall good, but slightly varied between radiotracers. Please see our response to R1.4 for more details. We briefly commented on this further in Results, *AATH model parameters are identifiable across experimental conditions* (page 7):

> The AATH model … spanning our three investigated radiotracers (Supplementary Table 2). Parameter estimation accuracy differed between radiotracers, (e.g., standard deviation of PS and K_1 estimates were lower for ^{18}F-FDG than ^{18}F-fluciclovine), but relative errors were small nonetheless. Our estimates of brain kinetics therefore appear reliable across radiotracers.

R1.13. *For some kind of validation, could you compare to the PS for each tracer to the relevant regional mRNA distribution for the appropriate transporter from the Allen Human Brain atlas? mRNA doesn't always lead to expression but it CAN and there may be several markers per transporter available to look at.*

Thanks for this interesting idea and we have carefully considered it. After some initial tests, we believe the investigation against the Allen Human Brain Atlas is deserving of its own detailed study and could not be completed in this paper. Additional expertise and resource are required to ensure all the methodological process is correct or well designed for conducting the proposed investigation. We also do not have paired BBB PS and mRNA data from the same subjects (which is very challenging to obtain for human brain). In addition, as the reviewer suggested, mRNA levels may not always lead to transporter expression, and therefore may not always be strongly correlated with our molecular PS measurement. However, we agree correlating with transporter expression is a good future direction for an in-depth study and thus we briefly commented on this in the Discussion (page 13):

> The interpretation … Further quantitation with Michaelis-Menten transporter kinetics could explain differences in molecular PS for facilitative transport.[64] To further characterize the biological significance of the BBB PS, a future study may investigate correlations between regional molecular BBB PS and transporter gene expression such as in the Allen Human Brain Atlas.[74]

We have also further expanded the discussion of the limitations of this work and indicated plans for additional studies on page 15 (also see the response to R1.16 below). Despite we could not pursue this mRNA study at

this stage, we have validated or evaluated our method in several other ways, including (1) demonstrating differences in BBB PS between three different PET tracers, (2) the consistency of CBF estimates between tracers, (3) the practical identifiability of CBF, K_1, and PS, (4) that FDG PS decreases with greater blood glucose levels as predicted by Michaelis-Menten transporter kinetics, and importantly (5) its clinical translation usefulness in studying liver-brain crosstalk in metabolic dysfunction-associated steatotic disease. These data together have laid the first groundwork for the proposed method to open more research on molecular BBB permeability of PET radiotracers and their applications to disease.

Discussion:

R1.14. *You mention the PS of gadolinium in the intro and that it increases in BBB breakdown & therefore increases the permeability measured. Would the same kind of leakiness seen in structural differences be also picked up in the PS of a PET tracer? What I mean is, that if there is a glioma or a stroke, or an MS lesion which changes the structural integrity of the BBB, I imagine it would let the small molecules pass through as it does the large gadolinium molecules, but would that also confound any changes brought to the molecular permeability changes? Would those two kinds of permeability be lumped in together? Thus you would see an increase due to leakiness from structural changes but then a decrease due to the molecular permeability and active transport mechanisms? Would it be possible to separate this? Can you please add a comment about what you would expect in this kind of situation?*

Thanks for this good question. Changes to the structural integrity of the BBB would also affect our PET tracer PS measurements. The reviewer is correct that the two types of permeabilities – that which comes from BBB "leakage" and that from the specific molecular transport mechanism – would be "lumped" together. We discussed this further in the Discussion (page 13):

> In comparison, DCE-MRI measures of BBB permeability mainly represent non-specific leakage of contrast material associated with BBB breakdown[8–10] as gadolinium contrast agents are not known to cross an intact BBB effectively.[5] … Although both processes likely occur simultaneously in aging, the resulting changes to DCE-MRI and FDG BBB PS greatly differ in scale (10^{-3} and 10^{-1} ml/min/cm^3, respectively). The effects of subtle BBB leakage were therefore likely obscured by the much greater changes in FDG BBB PS. Our current approach cannot explicitly differentiate the contribution of each transport mechanism to the measured BBB PS. Using DCE-MRI in conjunction with our PET method may help study molecular permeability when there is a more severe breakdown of the BBB, such as in brain tumours.[76]

R1.15. *Relatedly, I found it interesting that with the aging population you saw decreased PS and decreased CBF. Again, as above, I am surprised that there is no BBB structural breakdown showing an increase in PS - would you not expect this in an aging population? Perhaps the transporter is becoming less effective at transporting molecules, and therefore it decreases. A comment on this sort of confounding interaction in the discussion would be appreciated and it would be very interesting to test it in a case where there is some structural integrity change, and compare that to the DCE MRI for example.*

The BBB PS measured in DCE-MRI (≈10^{-3} ml/min/cm^3) are two orders of magnitude smaller than that of FDG BBB PS (≈10^{-1} ml/min/cm^3). Accordingly, an increase in subtle BBB leakage would likely be obscured by the greater decrease in FDG BBB PS. A comment has been added accordingly in the Discussion, as indicated in our revision to R1.14 shown above.

R1.16. *Can you discuss a way you could challenge the PS (or the CBF!) for different tracers and see if the difference can still be well separated? Could you potentially also do a DCE MRI to see how much of a PS is disturbed by the structural changes and how much is functional due to the molecular transport across the BBB? Or compare the CBF to an oxygen PET or a ASL-MRI study (or another MRI measure of CBF)?*

These are great suggestions that we are also considering to highlight the utility of our proposed method. We added these suggestions to the Discussion (page 14):

> A major limitation of this work is the lack of ground truth values in humans for validation of our PS measurements. … To further validate our method, future work includes a test-retest study to characterize the variability and repeatability of BBB PS estimates, validating the quantitative accuracy of CBF estimates against the gold standard (e.g., by ^{11}C-butanol PET),[17,26] as well as experiments manipulating CBF, BBB transporters (e.g., inducing hyperglycemia for ^{18}F-FDG), or transiently opening the BBB using MR-guided focused ultrasound[88] in the same subject to study resulting changes in BBB PS and other transport parameters. As well, it will be necessary to demonstrate our method across a wide range of in vivo physiological and pathophysiological conditions, such prolonged T_c due to small vessel disease[89] or for radiotracers that are rapidly excluded by the BBB.

In addition, as included in the revision responded to R1.14 above, we may also add a DCE-MRI scan in conjunction with our PET method to potentially separate the contributions due to structural and functional changes.

Reviewer #2 (Remarks to the Author):

*This work presents an interesting modeling approach that leverages the high sensitivity of new long-axial full body scanners to estimate cerebral blood flow and tracer transport rate into brain tissue (K1) from the same (single) scan dynamic data and thereby calculate an estimate of permeability*surface area product to infer BBB permeability from a single scan. This is an interesting method; however, more work is needed to establish the validity of this approach. The novelty of this method from a modeling perspective is the proposed estimation of CBF (and TC (mean vascular transit time)) from high temporal resolution data acquired during the first pass. The results must clearly demonstrate accurate estimation of CBF and separate identifiability of CBF from both E and TC. The challenge in parameter identifiability of CBF and E becomes evident if CBF*E is substituted for K1 in Eq. 4 (it is suggested to present the equation this way); with the current formulation that couples CBF and E estimation its extremely challenging to establish estimation of E independently of CBF. Several practical items are also unaddressed in discussion regarding extension of this potential method beyond the presented data.*

Thank you for this nice overview of our method and highlighting important questions that were unanswered in our original manuscript. In this revision, as suggested, we conducted additional simulation studies to show that CBF, K1, E, and PS had good practical identifiability (<5% mean error, <15% error standard deviation) despite any potential parameter covariance. We also expanded on the discussion of our method and results according to the comments by the reviewer.

R2.1. *The identifiability of CBF separately from T_C needs to be established through simulations, particularly for radiotracers with higher extraction fractions.*

Despite that the BBB PS is the main parameter of interest in this work, in this revision, we conducted additional practical identifiability experiments to support that CBF, K_1, and E are also identifiable (Supplementary Figure 1). Specifically, practical identifiability analysis was conducted by simulating different values of Tc (3 to 15 s), extraction fractions (1% to 99%), and typical CBF (0.25, 0.50, 0.75 ml/min/cm^3). We added a new Results

subsection "*AATH model parameters are identifiable across experimental conditions*" (pages 7-9). The following new text was added to specifically respond to the current question (page 8):

> The mean error of CBF was generally consistent across extraction fractions, but the standard deviation of the error decreased at higher extraction fractions. However, the identifiability of CBF differed with T_c and was worse for smaller values of T_c. Of note, a negative mean CBF error was observed for T_c = 3.0 s because the lower bound of T_c estimates was set at 3.0 s (Methods). The PS product – the main parameter of interest in this work – as well as the K_1, were not strongly affected by changes in T_c. The identifiability of all parameters improved with greater simulated CBF. Equations (2) and (4) show that CBF is a linear scaling factor of the AATH time-activity curve when E is fixed; accordingly, a greater CBF improves the signal-to-noise ratio of the time-activity curve when other parameters are kept constant ...

R2.2. *The simulation results examining 'practical identifiability of CBF and E' only tell the reader about accuracy and variance in E estimation for a single CBF value. The simulations should be expanded to a broader space of reasonably expected CBF values. Results addressing the identifiability of CBF from E must be shown. It is difficult to interpret Figures 4 and 5 without first demonstrating that CBF and E estimates with this model do not covary. A missing but critical piece is a demonstration that consistent PS estimates are made in settings where CBF is experimentally manipulated. While experimental data would be most convincing, simulations could help address this point.*

As pointed out by the reviewer, an experimental study manipulating CBF and assessing the reliability of PS estimates would be interesting and valuable, but it is not without critical technical challenges and requires a separate dedicated study for a solid investigation. As kindly suggested by the reviewer, we conducted a simulation study to show that consistent estimates of PS could be made when fixing PS and manipulating CBF. This result was added to Supplementary Figure 2b with the following text added in the manuscript (page 8):

> The mean error of CBF … Estimation of PS was also consistent when simulating a fixed PS while manipulating CBF (Supplementary Figure 2b).

Furthermore, we conducted additional simulation studies to show that our parameter estimates are reliable for different CBF values (low value 0.25, medium value 0.50, and high value 0.75 ml/min/cm^3). These results are reported in Supplementary Figure 1 and described in the new Results subsection "*AATH model parameters are identifiable across experimental conditions*" (page 7), also see response to R2.1.

R2.3. *The reported modeling results are quite sparse. Estimated values of T_C and T_d are not reported, but these are quite relevant to the successful implementation of this model and need to be added. Do the estimated t_C values make sense relative to what would be expected biologically?*

Supplementary Table 1 was added and summarizes the average ± standard deviation of td, Tc, and other model parameters. In a new Results paragraph (subsection *High-temporal resolution dynamic PET enables single-scan imaging of CBF and tracer-specific BBB transport rate*) (page 6), we described that our Tc estimates agreed with dual-tracer PET estimates of mean vascular transit time:

> Estimated mean vascular transit time T_c values ranged on average from 4 to 7 s for ^{18}F-fluciclovine and ^{18}F-FDG with longer vascular transit times in the white matter, agreeing with previous dual-tracer PET estimates.[25] For ^{11}C-butanol, our estimates of T_c and cerebral blood volume were greater than the expected physiological range (Supplementary Table 1). This may be due to the rapid extravasation and complete BBB extraction of ^{11}C-butanol causing the intravascular and extravascular spaces to become indistinguishable. Nonetheless, we consider T_c a method parameter and despite an overestimation of T_c,

our estimates of CBF and K_1 using ^{11}C-butanol were within expected physiological ranges and quantitative values.

R2.4. *An important consideration is the potential role for radiolabeled metabolites on this method when using the ascending aorta to estimate the arterial input function. All of the presented radiotracers ([18F]fluciclovine, [18F]FDG, [11C]Butanol) do not have significant radiolabeled metabolites, so this is not particularly relevant to the presented data. However, it is highly relevant for the host of radiotracers for brain targets mentioned in discussion lines 289-292, and this limitation for extension of this model to other radiotracers requires consideration.*

To address this question, we conducted additional practical identifiability analysis to study the effect of neglecting radiometabolites in our model. As a representative case, we used a population-based parent fraction of ^{18}F-florbetaben[6] to simulate the effect of radiometabolites in the arterial input function and its impact on our kinetic parameter estimates. Neglecting radiometabolites resulted in a systematic bias of approximately 5% in PS, K1, and E, with marginal changes to CBF and the standard deviation of parameter estimation errors. These results are summarized in Supplementary Figure 4 and we added new text in Results subsection *AATH model parameters are identifiable across experimental conditions* (page 8):

> The three investigated radiotracers had negligible radiometabolites during the 2-minute analysis time frame[26,47,53] …. To understand the effects of nonnegligible radiometabolite fraction …, we conducted practical identifiability analysis using tissue time-activity curves generated with a radiometabolite-corrected plasma input function based on a population-based parent fraction of ^{18}F-florbetaben[54] (Supplementary Figure 4). Here, approximately 80% of the parent compound remained in plasma at 2 minutes (Supplementary Figure 4a). Using the uncorrected plasma input function resulted in a systematic bias of approximately 5% in PS, K_1, and E, with marginal changes to CBF and the standard deviation of the parameter estimation errors. Despite the relatively small error, in practice, a population-based model may be used to correct the fraction of metabolites for the plasma input function, like many other studies using conventional kinetic modeling.

R2.5. *Please state in Methods the duration of the bolus injection of radiotracer for these studies.*

Added to Methods, subsection *High-temporal Resolution Dynamic Imaging with Total-body PET* (page 17):

> Dynamic PET imaging commenced immediately prior to bolus injection of the radiotracer. The bolus was rapidly injected by hand in 1 to 2 s for radiotracer volumes <1 ml for ^{18}F-FDG, <2 ml for ^{18}F-fluciclovine, and <6 ml for ^{11}C-butanol.

Reviewer #3 (Remarks to the Author):

Title: Quantitative PET imaging and modeling of molecular blood-brain-barrier permeability

General

This is an interesting piece of research using new kinetic modeling approaches to study blood-brain barrier permeability enabled by the high sensitivity of LAFOV pet/ct technology. The paper is very clearly written and the findings are of high interest to the scientific community. The limitations are acknowledged and well discussed. Yet, I have some comments that should be clarified.

Thank you for favourably reviewing our manuscript and your pertinent comments. We have answered these comments in detail below and made appropriate changes to the manuscript.

Minor comments

R3.1. *- Some references are self-centered. It may be good to refer to other LAFOV PET/CT systems as well as they also have ultra-high sensitivity*

Following the suggestion, we cited additional works and adjusted the location of the citations in the Introduction to better acknowledge the contributions of other groups to total-body PET and kinetic modeling. Specifically on total-body PET systems (refs 31 and 32 in manuscript), high-temporal resolution dynamic imaging (refs 24, 31, 33 in manuscript), image-derived arterial input functions (refs 29 and 36 in the manuscript).

R3.2. *- Some references to 15O-water pet may be good to mention. Radiowater is still considered to be the reference tracer for perfusion imaging.*

We added references in the Introduction (page 4) on cerebral blood flow PET imaging with ^{15}O-water (refs 13, 17–25) as well as ^{15}O-butanol (refs 20, 27). These were also cited in the first Results subsection to establish literature ranges for regional CBF obtained with flow-tracer PET.

R3.3. *- Some more details on the placement and size of the image derived input functions would be nice*

Added a new sentence in the *Image Analysis* subsection of the Methods (page 25):

Regions of interest were delineated using 3D Slicer[90] and by referring to a combination of dynamic and static PET frames and the attenuation correction CT. … For segmentation, we used a circular brush and the adjusted brush diameter according to the subject-specific size of the anatomical region. The median region of interest volume was ≈70 cm^3 for brain subregions and 6 cm^3 for the image-derived input function.

R3.4. *- Butanol and Fluciclovine show tracer metabolism. Are the input functions corrected for plasma to blood ratio and metabolites. If so, how. If not, please discuss implications for the models, it may affect k2 estimates? I understand that it may be a minor issue for the first 2 mins of the scan.*

^{18}F-fluciclovine does not have radiometabolites.[7,8] ^{11}C-butanol produces radiometabolites but is negligible during the first 2 minutes of the dynamic scan.[9]

To study the effect of neglecting radiometabolites, we conducted additional practical identifiability analysis. These results are summarized in Supplementary Figure 4 (please also see our response to R2.4) and we added new text in Results subsection *AATH model parameters are identifiable across experimental conditions* (pages 8-9):

… To understand the effects of nonnegligible radiometabolite fraction …, we conducted practical identifiability analysis using tissue time-activity curves generated with a radiometabolite-corrected plasma input function based on a population-based parent fraction of ^{18}F-florbetaben[54] (Supplementary Figure 4). … Using the uncorrected plasma input function resulted in a systematic bias of approximately 5% in PS, K_1, and E, with marginal changes to CBF and the standard deviation of the parameter estimation errors. Despite the relatively small error, in practice, a population-based model may be used to correct the fraction of metabolites for the plasma input function, like many other studies using conventional kinetic modeling.

In this work, we are not interested in estimating k2 and did not benchmark its identifiability. Even if k2 is not identifiable, the identifiability of our main parameter of interest (PS) is acceptable.

Major comments

R3.5. *- The AATH model ignores tissue fraction, as opposed to the standard single tissue model (using 1-Vb). The implication of not considering the tissue fraction should be discussed*

The tissue fraction (1-Vb) was "absorbed" into the K1 and as such K1 is expressed per unit "voxel" volume rather than "tissue" volume. For the brain, where the cerebral blood volume fraction is small (~5%),[10,11] the difference in computing K1 per unit tissue volume or per unit voxel volume is negligible. We elaborated on this in the Methods subsection *High-Temporal Resolution Kinetic Modeling and Measuring BBB PS* (page 20):

> Of note, the tissue volume fraction, $v_t = 1 - v_b$, commonly seen as a scaling factor of the second term of Equation (7), is included as a part of K_1. This was chosen to be consistent with the original AATH model[46] and is also commonly used in DCE-MRI studies.[44,98] K_1 is therefore expressed per unit voxel volume as opposed to tissue volume. CBF and PS also follow this convention. As the brain has a cerebral blood volume fraction of ≈5%,[19,25] differences in absolute values due to following this convention is negligible.

R3.6. *- Should future work aim to merge both modelling approaches?*

As described in our response to R3.5, excluding (1-Vb) does not make a substantial difference in the brain due to its small Vb. Furthermore, it can be shown that the S1TC model is a special case of the AATH model when Tc approaches 0. As such, the CBF term in Equation (4) (AATH model) replaces the v_b term in Equation (7) (S1TC model), so an extra v_b term is not needed in the AATH model. We elaborated on our description and interpretation of the AATH model in the Methods (*High-Temporal Resolution Kinetic Modeling and Measuring BBB PS)* (page 19):

> … The S1TC differs from the AATH model as it assumes instantaneous distribution of tracer in the intravascular space and neglects the finite transit time required for tracer to traverse the blood vessel volume (i.e., $T_c = 0$). As such, the S1TC response function lacks a finite-length vascular phase and the model describes that tracer is immediately cleared from tissue at $t > 0$. This is consistent with the observation that the AATH impulse response function is equal to that of the S1TC model when substituting $CBF = v_b/T_c$ in Equation (2) and taking the limit as T_c approaches 0.

R3.7. *- Please add assessment of CBF grey/white matter ratio's for all 3 tracers to see if regional CBF values are comparable using the various models for the 3 tracers…they should if the estimate of extractions or PS is accurate. We now can compare the reported CBF only with some population values.*

We added Supplementary Table 1 summarizing regional brain kinetics, including grey and white matter CBF, for each investigated radiotracer. Also added to the Results subsection *High-temporal resolution dynamic PET enables single-scan imaging of CBF and tracer-specific BBB transport rate* (page 6):

> White to grey matter CBF ratios were 0.359±0.048, 0.354±0.069, and 0.395±0.032 for ^{18}F-fluciclovine, ^{18}F-FDG, and ^{11}C-butanol, respectively, in agreement with other flow-tracer brain PET studies.[19,20,25]

R3.8. *- A parametric as well as regional comparison of K1 estimated using AATH and the conventional kinetic model per patient for each tracer would be nice. In particular for butanol with a near 100% extraction the data*

points should be the same and align along the line of identity. If not, please discuss the reason for it (in case of butanol, for the other tracers it should align with the average extraction fraction)

Below in Figure R1 we have plotted the correlation between AATH and S1TC K_1 values for ^{18}F-fluciclovine, ^{18}F-FDG, and ^{11}C-butanol studied in this work. Indeed, the two estimates are strongly correlated as expected but the linear regression slope is not equal to 1 as the S1TC K_1 is generally greater than that of the AATH model. This is because the S1TC model is effectively fitting while Tc = 0 s and underestimates the vascular transit time. This leads to an overestimation of the tissue distribution and accordingly the K_1. An example comparison of FDG grey matter time-activity curve fits is shown in Figure R2, where the extravascular component of the fitted curve (green dashed line) is larger in the S1TC fit than the AATH fit, resulting in S1TC K_1 > AATH K_1.

This data has not been included in the manuscript as this comparison is not critical to the main message of this work. We plan to describe this difference in greater detail in a separate future work, and further explore whether the difference in S1TC K_1 and AATH K_1 differs between radiotracers (e.g., with lower extraction fraction) and regions (with different CBF, Tc, etc.). We hope the reviewer is okay with our decision and plan.

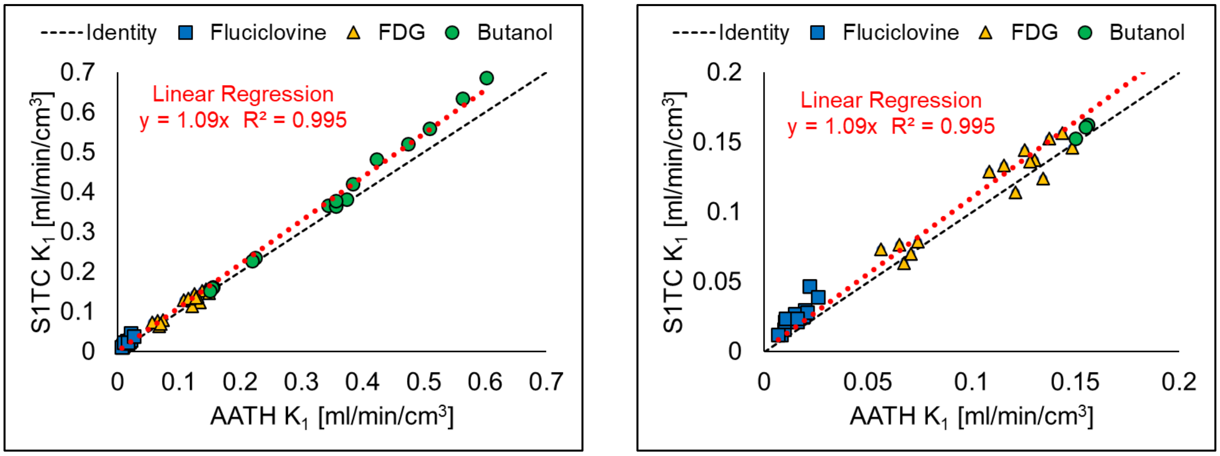

Figure R1. AATH vs S1TC K_1 correlation plots across PET tracers. Left: shown is the entire range of K_1 values; right: the axes have been zoomed in to 0 to 0.2 ml/min/cm^3.

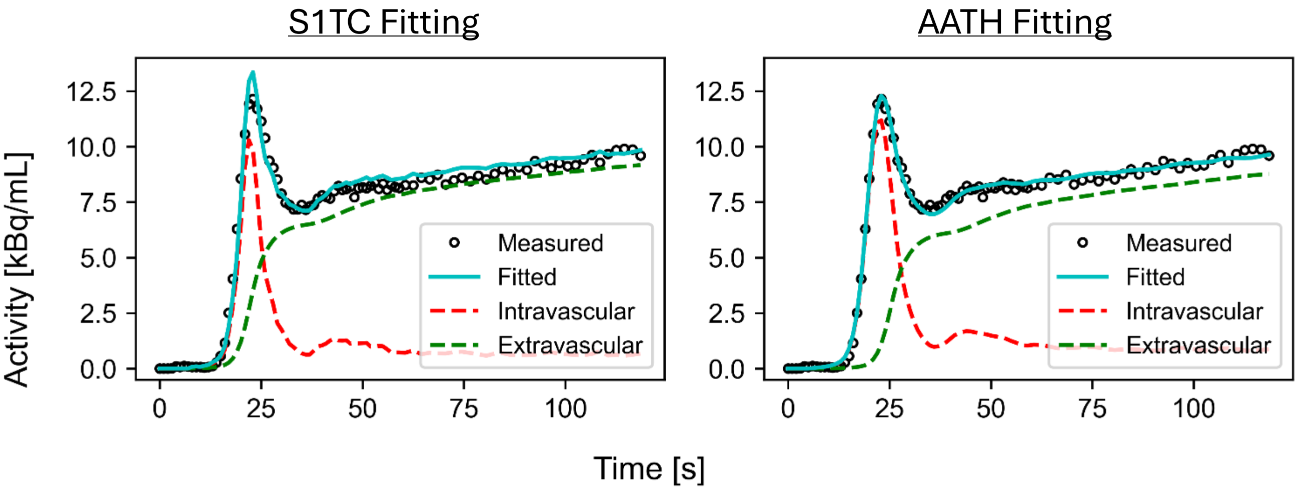

Figure R2. S1TC vs AATH model fits in FDG grey matter time-activity curves. The extravascular component (green dashed line) is larger in the S1TC over the AATH fit. This results in a greater estimate of K_1 over the AATH.

R3.9. *- How do you deal with delay and dispersion, in particular for the AATH model. I recommend to add some simulations with 0.25,0.5,1.0 and 2.0 s errors in the delay estimation (in both directions) and how that would affect the observed E (or PS) and CBF values.*

Time delay (td) was explicitly modeled and estimated as a part of the curve fitting (described in Methods, Eq. (3), *High Temporal Resolution Kinetic Modeling and Measuring BBB PS*). We added a new Supplementary Table 1 showing regional kinetics including t_d, which was on average 2 to 3 s across the three investigated radiotracers.

Additional results on the effect of error in td are shown in Supplementary Figure 1 and Supplementary Figure 3 and described in the Results subsection "AATH model parameters are identifiable across experimental conditions" (page 8). An error in the estimation of td affects the estimation of other parameters like CBF and E. Nonetheless, our practical identifiability analysis shows that our parameters of interest (PS, K1, CBF, E) have low bias (<5%) and standard deviation (<15%) across a wide range of kinetics (Supplementary Figure 1). Supplementary Figure 3 shows an example for one parameter set (representative of ^{18}F-FDG) where, when td is underestimated, CBF is also underestimated (column 1, row 3). The two parameter estimates are strongly correlated across noise realizations (R = 0.94, p<0.001). This trend was opposite for E (underestimated td --> overestimated E). The trend of td versus PS estimates was much weaker but still significant (R = -0.37, p<0.001).

Dispersion was not explicitly modeled in our work. Meyer[12] previously approximated arterial dispersion to be 1 s between the heart and brain.[12] Figure R3 in response to R4.4 shows that a dispersion of 1 s produces a very small effect on a representative ascending aorta image-derived arterial input function. We added a comment about dispersion in the Methods, *High-Temporal Resolution Kinetic Modeling and Measuring BBB PS* (page 20):

> For all investigated tracers and for both AATH and S1TC methods, we assumed an absence of metabolites[26,47,53] and that the whole-blood tracer activity was equal to that in blood plasma over the first two-minutes of the dynamic PET scan. Similar to a recent study that also uses 1-s high temporal resolution dynamic PET,[24] we assumed that arterial input function dispersion was negligible due to the relatively short distance between the ascending aorta and the brain.

R3.10. *- Are 2 min scan duration optimal for both models? Some data on shorter or longer scan durations would be nice. Why choose 2 min.*

The choice of a 2-minute scan duration was guided by several earlier works estimating FDG K_1 from the first 2-mins of the dynamic scan to approximate blood flow. Currently, studying the effect of scan duration is challenging for tracers like FDG because the effect of phosphorylation cannot be neglected for longer scan durations. We added the following in the Discussion (page 14):

> Advanced kinetic models … as in our approach. The use of the first two minutes for our method was guided by prior studies using early-dynamic FDG-PET to approximate blood flow using FDG K_1[83,84] as well as to minimize the effect of phosphorylation in the model.[34,85] In future work, we will optimize the protocols and compare the strengths and weaknesses of each method in measuring and quantifying CBF and PS. Furthermore, the AATH model will be extended to allow additional tissue compartments such as the phosphorylation of ^{18}F-FDG.

R3.11. *– Does the administration protocol matter…so the speed of administration. For sure you would need a fast bolus administration for the AATH model, but how would the model respond if the administration was a bit dispersed due to issues in the administration line.*

To address this question, we conducted additional simulation studies to characterize the accuracy of our parameter estimates when varying the shape of the arterial input function, the shape (e.g., sharpness of the first pass) in large part determined by the administration protocol. We added a paragraph in the Results subsection *AATH model parameters are identifiable across experimental conditions* (page 8):

> The three investigated radiotracers … we further studied how the characteristics of the arterial input function impacts the identifiability of AATH parameters. … Second, we studied the effect of using a slower injection protocol by simulating three levels of dispersion in the arterial input function (Supplementary Figure 5). The simulated slower injections had the most impact on the identifiability of CBF, with error mean and standard deviation increasing with greater levels of dispersion. This also affected the standard deviation of E estimates, but otherwise appeared to have a small effect on the identifiability of PS and K_1.

R3.12. *- What were the radtiotracer administration settings (speed, volume, etc). Also here it would be good to have some simulations or orther data to get idea on how critical this is.*

Added to Methods, subsection *High-temporal Resolution Dynamic Imaging with Total-body PET* (page 17):

> Dynamic PET imaging commenced immediately prior to bolus injection of the radiotracer. The bolus was rapidly injected by hand in 1 to 2 s for radiotracer volumes <1 ml for ^{18}F-FDG, <2 ml for ^{18}F-fluciclovine, and <6 ml for ^{11}C-butanol.

Simulations on the effect of the shape of the arterial input function (in large part determined by the radiotracer administration settings) were described in our response above to R3.11.

Reviewer #4 (Remarks to the Author):

This manuscript details the methodological framework for in vivo measurement of the blood-brain barrier transport using high temporal resolution PET imaging in humans. Measurements of permeability-surface area (PS) product are made across three radiotracers and demographic/clinical conditions of aging and MASH. A distributed model was applied for parameter estimation demonstrating the uncoupling of cerebral blood flow and PS measures as well as variability in PS across conditions. The ability to measure BBB permeability via the PS metric is a highly significant methodological advancement in neuroimaging over existing methods (e.g. DCE-MRI), making a potentially profound impact on advancing the basic understanding of BBB physiology as well as translation into clinical care. Further, it demonstrates the scientific utility of the emerging technology of total body PET imaging systems which afford the opportunity of rapid temporal imaging due to increased field-of-view and high detection sensitivity acquisitions. The manuscript is well written providing detailed descriptions of the experiments performed to carefully examine BBB permeability across conditions and results that support the tested hypothesis. Because a somewhat nonstandard analytic method for dynamic PET data was used, additional details and background are warranted to assuage the concerns with a lack of parameter identifiability in the measured outcomes. The presentation of the findings and clarity of the manuscript would be greatly improved by addressing the following concerns:

AATF model is nonstandard compared to compartment models) for dynamic PET time-series data and additional details equating it to S1TC and S2TC would aid the reader with interpreting these outcomes without having to closely study the Lawrence & Lee (1998) paper. Specifically:

Thank you for this comprehensive summary of our methodology and findings. In response to these recommendations, we have considerably elaborated on the characterization and description of the AATH model. Our responses to specific comments are below.

***R4.1.** - Distinguishing CBF from K1 in equation 2 is confusing, particularly with referring to K1 as the BBB transport rate. The relationship of K1 = CBF*E should be defined here to aid in the interpretation of this parameter. Further, the parameter "F" is mentioned on this page and supplementary figure 2, but not defined.*

We have clarified the description of K1 following Equation 2 (page 18):

> … where CBF is the cerebral blood flow (ml/min/cm^3), T_c is the mean vascular transit time (s), K_1 is the BBB transport rate (ml/min/cm^3) of the radiotracer and the product of CBF and extraction fraction ($K_1 = CBF \cdot E$), k_2 is in BBB clearance rate (min^{-1})

The F in Supplementary Figure 2 (now Supplementary Figure 6) was a typo and should have been CBF. This has been corrected. Thank you for noticing this typo.

***R4.2.** – Please provide a more clear definition of the vascular transit time "Tc" as it appears to be the distinguishing feature between the AATH and S1TC models. Specifically if it references the tissue capillary transit time to denote a specificity to the region of perfused tissue as separate from the vascular space between aortic sampling and (through the) tissue of interest.*

Following the suggestion, we clarified the definition of Tc in Methods, *High-Temporal Resolution Kinetic Modeling and Measuring BBB PS* (page 18). We use the notation Tc to be consistent with the original AATH paper.[14]

> Of note, the mean vascular transit time T_c describes the average time required for tracer to traverse the entire intravascular volume residing in a voxel, including arteries, arterioles, capillaries, venules, and veins.

We also added the following new data about Tc estimates with our proposed method in Results, *High-temporal resolution dynamic PET enables single-scan joint imaging of CBF and tracer-specific BBB transport rate from a single dynamic scan* (page 6):

> Estimated mean vascular transit time T_c values ranged on average from 4 to 7 s for ^{18}F-fluciclovine and ^{18}F-FDG with longer vascular transit times in the white matter, agreeing with previous dual-tracer PET estimates.[25] For ^{11}C-butanol, our estimates of T_c and cerebral blood volume were greater than the expected physiological range (Supplementary Table 1). This may be due to the rapid extravasation and complete BBB extraction of ^{11}C-butanol causing the intravascular and extravascular spaces to become indistinguishable. Nonetheless, we consider T_c a method parameter and despite an overestimation of T_c, our estimates of CBF and K_1 using ^{11}C-butanol were within expected physiological ranges and quantitative values.

***R4.3.** - Equation 4, please check to limits of integration. For the CBF term, it would seem the first term would only be integrated from 0->Tc (assuming a delay corrected Ca) and the K1 term from Tc -> t.*

The confusion might stem from putting $t - T_c$ in the upper bound, which we can remedy by performing a change of variables. Our fitting results were not affected by this notation change. We also modified the lower limit of integration to indicate that the integral is zero when t is less than the lower bound (Equation (4), Methods, High-Temporal Resolution Kinetic Modeling and Measuring BBB PS, page 8):

$$Q(t) = CBF\left[\int_{t_d}^{t} C_{wb}(\tau - t_d)\,d\tau - \int_{t_d+T_c}^{t} C_{wb}(\tau - t_d - T_c)\,d\tau\right] + K_1\int_{t_d+T_c}^{t} C_p(\tau - t_d - T_c)e^{-k_2(t-\tau)}H(t-\tau)\,d\tau \quad (4)$$

where each integral is zero when t is less than its respective lower limit of integration. The first integral describes the accumulation of tracer in blood and tissue due to its arterial delivery by CBF. The second integral describes the tracer's venous clearance by CBF after the mean vascular transit time, T_c. The third integral describes tracer extraction across the BBB, return of the extracted tracer back to blood, and its subsequent venous clearance from the voxel volume.

R4.4. *- Given the necessity for rapid sampling of the tissue, it would appear that some type dispersion in Ca (eq 3) should be included in the model (Hutchins 1986; Meyer 1989) to account for differences between aortic and regional tissue vasculature for examining the effects on CBF and K1 estimates.*

Early works that highlight dispersion correction were between radial artery and brain, where dispersion was around 4-6 s. Meyer 1989 *J Nucl Med* (cited by the reviewer)[12] suggested that the time constant of dispersion between the heart and brain may only be approximately 1 s. Figure R3 below shows that a dispersion of 1 s produces a very small effect on the arterial input function. Thus, we did not include dispersion correction using a justification similar to a recent study.[13] We commented about dispersion in the Methods, *High-Temporal Resolution Kinetic Modeling and Measuring BBB PS* (page 20):

> For all investigated tracers and for both AATH and S1TC methods, we assumed an absence of metabolites[26,47,52] and that the whole-blood tracer activity was equal to that in blood plasma over the first two-minutes of the dynamic PET scan. Similar to a recent study that also used 1-s high temporal resolution dynamic PET,[24] we assumed that arterial input function dispersion was negligible due to the relatively short distance between the ascending aorta and the brain.

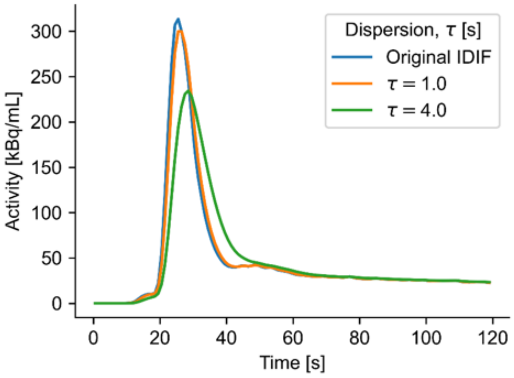

Figure R3. Simulated dispersed arterial input function with dispersion rate constants $\tau = 1$ s and 4 s using $C_d(t) = \frac{1}{\tau}C_a(t) \otimes e^{-\frac{t}{\tau}}$

R4.5. *- Coupled with the above issue is the "sharpness" of the bolus injection. A short, rapid bolus injection is typically needed to uncouple K1 and k2 using the 1TC model. Is this also required for the estimation of CBF and K1 with the AATH model? Would equation 2) still apply for a long Tc with respect to rapid extravascular clearance? There are no details regarding the injection of any of the radiotracers, e.g. 5- or 30-sec infusion. Was*

it closely monitored for all of the studies. Details regarding the shape of AIF bolus with CBF and K1 identifiability should be presented.

Yes, a rapid bolus injection was used for the proposed method in all the studies. We added the following description about the bolus injection protocol to the Methods, subsection *High-temporal Resolution Dynamic Imaging with Total-body PET* (page 17):

> Dynamic PET imaging commenced immediately prior to bolus injection of the radiotracer. The bolus was rapidly injected by hand in 1 to 2 s for radiotracer volumes <1 ml for ^{18}F-FDG, <2 ml for ^{18}F-fluciclovine, and <6 ml for ^{11}C-butanol.

Furthermore, we conducted an additional simulation study to characterize the identifiability of AATH model parameters (in particular, PS, K_1, CBF, E) when a slower injection protocol was used. We added a paragraph in the Results subsection *AATH model parameters are identifiable across experimental conditions* (page 8):

> The three investigated radiotracers … we further studied how the characteristics of the arterial input function impacts the identifiability of AATH parameters. … Second, we studied the effect of using a slower injection protocol by simulating three levels of dispersion in the arterial input function (Supplementary Figure 5). The simulated slower injections had the most impact on the identifiability of CBF, with error mean and standard deviation increasing with greater levels of dispersion. This also affected the standard deviation of E estimates, but otherwise appeared to have a small effect on the identifiability of PS and K_1.

Conceptually, Equation (2) would still apply in cases where Tc is large and extravascular clearance is fast. Our studied tracers did not demonstrate a rapid extravascular clearance, and more work is needed to characterize our method on those situations. We acknowledged this in the Discussion (page 15):

> A major limitation of this work is the lack of ground truth values in humans for validation of our PS measurements. … To further validate our method, … As well, it will be necessary to demonstrate our method across a wide range of in vivo physiological and pathophysiological conditions, such prolonged T_c due to small vessel disease[89] or for radiotracers that are rapidly excluded by the BBB.

R4.6. *- please define the 5 parameters that are estimated from equation 4, assuming that time delay correction has already been applied. Although the noise simulation methods for determining parameter identifiability are sound and figure 1S compelling, there remains a concern of high covariance between many of the estimated parameters, particularly with td and Tc. It would be helpful to include some commentary on the limitations of accurate parameter identifiability.*

We have clarified which model parameters to be estimated in the Methods subsection *(right* following Equation (5), page 19):

> … where $\boldsymbol{\theta} = [CBF, K_1, k_2, T_c, t_d]$ are the model parameters to be estimated, …

Additionally, a new Results subsection *AATH model parameters are identifiable across experimental conditions* (pasted below) was added to further support that our method is robust and allows reliable estimation of these parameters across a wide range of tracer kinetics, despite any potential covariance between parameters. We further highlight that the BBB PS is the main parameter of interest in this work. Limitations are also discussed in the same subsection (page 8).

> We then … The practical identifiability of PS was generally excellent …, but deteriorated at very low (E<2.5%) and high (E>75%) extraction fractions (Supplementary Figure 2). At very low values of E, the identifiability of K_1 and E worsened as only a small proportion of radiotracer is extravasated into the brain,

and accordingly, the identifiability of PS is poor. Similarly, for high values of E, a small change in extraction fraction can cause a large change in PS (Equation 1).

The mean error of CBF … However, the identifiability of CBF differed with T_c and was worse for smaller values of T_c. … The PS product – the main parameter of interest in this work – as well as the K_1, were not strongly affected by changes in T_c …

Sensitivity analysis … Despite these correlations, our practical identifiability analysis showed that our parameters of interest had low bias (<5%) and standard deviation (<15%) across a wide range of kinetics.

R4.7. *- the assumption is made that no radiolabeled metabolites are present, it would be helpful to know if their presence is negligible on the measured outcomes. Similarly, this could also confirm that varying radiochemical purity (frequently between 90%-100%) of the injected PET drug product can be neglected.*

We conducted additional simulation studies to evaluate the effect of neglecting radiometabolites in the arterial input function and on the practical identifiability of AATH model parameters. The results are summarized in Supplementary Figure 4 and we added new text in Results subsection *AATH model parameters are identifiable across experimental conditions* (page 8):

The three investigated radiotracers had negligible radiometabolites during the 2-minute analysis time frame[26,47,53] … To understand the effects of nonnegligible radiometabolite fraction …, we conducted practical identifiability analysis using tissue time-activity curves generated with a radiometabolite-corrected plasma input function based on a population-based parent fraction of ^{18}F-florbetaben[54] (Supplementary Figure 4). Here, approximately 80% of the parent compound remained in plasma at 2 minutes (Supplementary Figure 4a). Using the uncorrected plasma input function resulted in a systematic bias of approximately 5% in PS, K_1, and E, with marginal changes to CBF and the standard deviation of the parameter estimation errors. Despite the relatively small error, in practice, a population-based model may be used to correct the fraction of metabolites for the plasma input function, like many other studies using conventional kinetic modeling …

R4.8. *- a limitation should also be included noting the lack of test/retest variability in the PS and CBF estimates, including paradigms that alter CBF (e.g. hypercapnic conditions).*

In the Discussion, we commented on future experiments for test-retest reproducibility and manipulating CBF and saturating BBB transporters (page 15):

A major limitation of this work is the lack of ground truth values in humans for validation of our PS measurements. … To further validate our method, future work includes a test-retest study to characterize the variability and repeatability of BBB PS estimates, … as well as experiments manipulating CBF, BBB transporters (e.g., inducing hyperglycemia for ^{18}F-FDG), or transiently opening the BBB using MR-guided focused ultrasound[88] in the same subject to study resulting changes in BBB PS and other transport parameters …

We also added in the limitations (page 15-16):

This study also had other limitations. … Lastly, we did not scan the same participant with each of our three investigated radiotracer when comparing BBB PS across tracers. As such, subject-specific CBF and extraction may have potentially confounded our PS comparisons, but these effects were likely small relative to the order of magnitude differences in BBB PS we observed between radiotracers.

Other Issues:

***R4.9.** Intro, p.3, para2 – PET studies with butanol have also been conducted with [O-15]butanol, which would seem to be a better option for this application. It should at least be mentioned that [O-15]butanol would also be an option for these measurements.*

Added to the Introduction (page 4):

> Currently, two PET scans … flow radiotracer (e.g., ^{15}O-water,[13,17–25] ^{11}C-butanol,[17,18,26] or ^{15}O-butanol[20,27]).

***R4.10.** Intro, p.3, last sentence – the ability of TB PET to measure image-based AIFs may be oversold a bit, given the need for external metabolite correction for most radiotracers.*

We have briefly noted this limitation in the first subsection of Results (page 4):

> [Total-body PET's] extended axial field-of-view …, obviating the need for invasive arterial blood sampling with minimal delay and dispersion effects[39] for PET kinetic analysis (Figure 2a) of radiotracers without significant radiometabolites.[29]

We also added in the discussion (page 14):

> Beyond ^{18}F-FDG, …, radiotracers with radiometabolites may require arterial blood sampling for metabolite correction …

***R4.11.** p.12, para 1, please provide the effective spatial resolution of the reconstructed images used for this investigation, assuming the selected voxel size (2.344-mm) would not reproduce NEMA-based measures.*

Using a ^{22}Na point source scan reconstructed with the same voxel size and reconstruction settings as used in our human imaging studies, we found that the full width at half maximum resolution was 3.3 mm at the approximate axial position corresponding to the brain and 3.7 mm for the axial position near the heart.

Spatial resolution depends on several factors including the reconstruction method (FBP vs OSEM), the use of point spread function modeling, voxel size, etc. NEMA provides one way of standardizing spatial resolution measurements to allow fair comparisons between systems. As such, we propose to keep the NEMA spatial resolution measurement originally provided in our paper ("≈3.0 mm full width at half maximum resolution by the NEMA standard", page 17).

***R4.12.** p.12, para 2 – knowing that many millions of radiological imaging scans are conducted yearly using ionizing radiation to provide invaluable clinical information, it is not evident why the involvement of radiation exposure (at safe levels) is a limitation. The dosimetry details here and throughout the Methods section seem misaligned to the overall theme of this manuscript focusing on methodological development.*

Because the journal has a broad audience, some of whom may be concerned about the PET method compared to MRI, we think it is still worth mentioning that this PET method involves ionizing radiation exposure and providing some specific dosimetry data. However, we agree with the reviewer that perhaps this is not a limitation considering this work's theme on methodological development. We moved the discussion of radiation dose to a different paragraph in the discussion but kept the effective dose information in the Methods section and noted the following (Methods, *High-temporal Resolution Dynamic Imaging with Total-body PET*, page 17):

> The effective dose of PET scans … resulting in a mean effective dose of 7.0 mSv, 6.8 mSv and 1.1 mSv for the three studies, respectively. These effective doses are well within the acceptable range for healthy

subjects as compared to the average annual natural background radiation of 3.1 mSv in the United States. Protocols were approved by our institutional review board and ethics committee.

R4.13. *p.15, para 3, the first sentence ends with stating that S1TC can only estimate K1, which leads to the following sentence stating that PS can be derived from K1. Presumably this applies only to the AATH model. If so, please clarify that PS was not estimated from the S1TC K1.*

As suggested, we clarified it in the Methods, *High-Temporal Resolution Kinetic Modeling and Measuring BBB PS* (page 20):

> An advantage of the AATH model[46] ... The PS product (Equation (1)) can then be calculated from the AATH CBF and AATH K_1 using a single-tracer dynamic PET scan by rearranging the Renkin-Crone equation[49,50]

R4.14. *p.15, last paragraph, one of the often stated advantages of TB PET is the potential for injected dose reduction, yet these experiments appear to give standard clinical amounts. It's understandable that high counting statistics should be sought for this methodology development, however, it would be helpful if the authors could comment on the accuracy of the AIF and outcome measures for a reduced dose study.*

Our MASLD study with total-body PET was conducted with an average ^{18}F-FDG injected activity of 186±13 MBq (5.0±0.4 mCi), approximately half that of conventional ^{18}F-FDG injected activities at our institution. Our method was able to extract high-quality IDIFs and detect significant differences in FDG BBB PS between clinically relevant groups. As such, we expect our method to be reasonably feasible at lower injected activities, especially on a regional level. Parametric imaging is admittedly more challenging at lower injected activities as the signal-to-noise ratio is poorer at the voxel level. We added text to the discussion (page 12):

> The proposed method … Advances in brain-dedicated PET imagers[67] and image reconstruction methods[68,69] … Our MASLD study with total-body PET used approximately half the injected activity used for conventional FDG-PET (Methods) and nevertheless our proposed method was able to detect significant differences in FDG BBB PS across clinically-relevant groups. In the future, we will investigate the feasibility of our proposed method for both regional quantification and voxel-wise parametric imaging with reduced injected activities.

R4.15. *Figure 1 – this is a very nicely crafted and informative illustration of the concepts being investigated in this study. One suggestion would be to explicitly state in the legend or body text that leakage as visualized by junction openings in a) can also be present in b) - d) but can be neglected due to the relative differences in scale of PS product.*

Added to the caption of Figure 1:

> Of note, the non-specific BBB leakage depicted in (a) can be present in (b) to (d) but can be neglected due to the relative difference in scales of BBB PS.

R4.16. *Figure 2b –illustration of AIF with varying temporal sampling. Although it could be obscured by the time-axis scaling, it does not appear that the integrals (i.e. AUC) for the 1-, 5-, 10-sec curves are equivalent as would be expected from total counts divided by frame duration of each point. Can an explanation be given for this?*

This indeed appears to be a time scaling effect. Below (Figure R4) is the plot zoomed into the first 60 seconds showing that at lower temporal resolutions, certain areas are underestimated (e.g., 5 to 15 s at 10 s

intervals) and others are underestimated (e.g., 15 to 25 s at 10 s intervals). To confirm, we computed the area underneath the curve of each temporal resolution for these specific curves, and they were 66.5, 66.4, and 65.8 kBq·min/mL, at 1, 5, and 10 s framings, respectively. The difference in areas between 1 s and 10 s framings is marginal ≈1%.

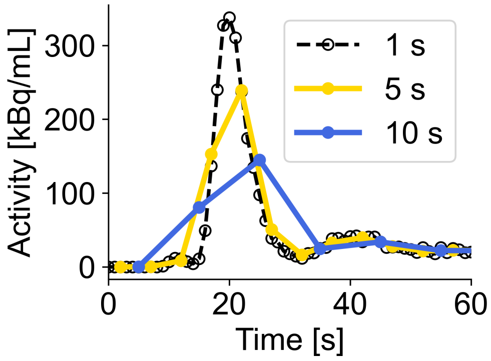

Figure R4. Image-derived input function plot in Figure 2 (main manuscript) zoomed to the first 60 seconds

R4.17. *Figure 3b – the AATH model estimates CBF and K1, and presumably the E (=K1/CBF) and PS images are calculated by equation 7. This isn't evident from the figures for fluciclovine and FDG. The extraction image (E) does, to some degree, reveal enhanced signal consistent with K1. However, lower signal in E does not appear to track with the high signal in CBF (particularly in fluciclovine). Is this an artifact of colorbar scaling?*

No, it is not an artifact of colorbar scaling. E does not always track CBF but depends on the permeability of the tissue type to the specific radiotracer/molecule. Using the ^{18}F-fluciclovine data in Figure 3b as an example, we commented on this in the Results subsection *Molecular BBB PS Differs Across PET Radiotracers* (page 7):

> CBF maps (Figure 3b) appeared visually comparable between PET tracers, though the prominence of veins (e.g., sagittal sinus) appeared to decrease for radiotracers with higher PS possibly due to their higher tissue extraction leaving less radiotracer concentration cleared through the venous circulation. Of note, E is not always coupled with CBF, as higher ^{18}F-fluciclovine E in the choroid plexus did not result in a higher CBF, and conversely, low ^{18}F-fluciclovine E in the brain parenchyma did not result in a low CBF.

R4.18. *Figure 6b – please give consideration to the precision of the estimated Vmax and Km listed on the figure and the text on p.8, para 3. Given the high variability shown in the figure, it would seem this precision in the estimates is excessive.*

Figure 6b has been updated such that the estimated Michaelis-Menten parameters have 1 decimal place.

References

1. Mankoff, D. A. *et al.* Kinetic analysis of 2-[carbon-11]thymidine PET imaging studies: compartmental model and mathematical analysis. *J Nucl Med* **39**, 1043–1055 (1998).

2. Mankoff, D. A., Muzi, M. & Zaidiy, H. Quantitative Analysis in Nuclear Oncologic Imaging. in *Quantitative Analysis in Nuclear Medicine Imaging* (ed. Zaidi, H.) 494–536 (Springer US, Boston, MA, 2006). doi:10.1007/0-387-25444-7_16.

3. Zuo, Y. *et al.* Structural and practical identifiability of dual-input kinetic modeling in dynamic PET of liver inflammation. *Phys. Med. Biol.* **64**, 175023 (2019).

4. Yoshida, K., Mullani, N. & Gould, K. L. Coronary flow and flow reserve by PET simplified for clinical applications using rubidium-82 or nitrogen-13-ammonia. *J Nucl Med* **37**, 1701–1712 (1996).

5. Zuo, Y. *et al.* Multiparametric cardiac ^{18}F-FDG PET in humans: pilot comparison of FDG delivery rate with ^{82}Rb myocardial blood flow. *Phys. Med. Biol.* **66**, 155015 (2021).

6. Patt, M. *et al.* Metabolite analysis of [18F]Florbetaben (BAY 94-9172) in human subjects: a substudy within a proof of mechanism clinical trial. *J Radioanal Nucl Chem* **284**, 557–562 (2010).

7. Shoup, T. M. *et al.* Synthesis and evaluation of [18F]1-amino-3-fluorocyclobutane-1-carboxylic acid to image brain tumors. *J Nucl Med* **40**, 331–338 (1999).

8. Michaud, L. *et al.* 18F-Fluciclovine (18F-FACBC) PET imaging of recurrent brain tumors. *Eur J Nucl Med Mol Imaging* **47**, 1353–1367 (2020).

9. Li, E. J. *et al.* Total-Body Perfusion Imaging with [11C]-Butanol. *J Nucl Med* **64**, 1831–1838 (2023).

10. Rostrup, E. *et al.* The relationship between cerebral blood flow and volume in humans. *NeuroImage* **24**, 1–11 (2005).

11. Leenders, K. L. *et al.* CEREBRAL BLOOD FLOW, BLOOD VOLUME AND OXYGEN UTILIZATION: NORMAL VALUES AND EFFECT OF AGE. *Brain* **113**, 27–47 (1990).

12. Meyer, E. Simultaneous correction for tracer arrival delay and dispersion in CBF measurements by the H215O autoradiographic method and dynamic PET. *J Nucl Med* **30**, 1069–1078 (1989).

13. Larsson, H. B. W. *et al.* Brain perfusion estimation by Tikhonov model-free deconvolution in a long axial field of view PET/CT scanner exploring five different PET tracers. *Eur J Nucl Med Mol Imaging* **51**, 707–720 (2024).

14. St Lawrence, K. S. & Lee, T.-Y. An Adiabatic Approximation to the Tissue Homogeneity Model for Water Exchange in the Brain: II. Experimental Validation. *J Cereb Blood Flow Metab* **18**, 1378–1385 (1998).

15. Berridge, M. S. *et al.* Measurement of Human Cerebral Blood Flow with [^{15}O]Butanol and Positron Emission Tomography. *J Cereb Blood Flow Metab* **11**, 707–715 (1991).

16. Wolburg, H. & Paulus, W. Choroid plexus: biology and pathology. *Acta Neuropathol* **119**, 75–88 (2010).

17. Zhao, L., Taso, M., Dai, W., Press, D. Z. & Alsop, D. C. Non-invasive measurement of choroid plexus apparent blood flow with arterial spin labeling. *Fluids Barriers CNS* **17**, 58 (2020).

Response to Reviewer Comments

Changes in the manuscripts are marked in red.

Reviewer #1

Review of rebuttal: Quantitative PET imaging and modeling of molecular blood-brain barrier permeability

The extra results generated by the authors, and the modifications to the manuscript have satisfied my queries.

No more changes required, thank you.

Reviewer #2

The revisions contain an impressive amount of work in support of their model. While I remain uncomfortable with a model that relies so heavily on estimating a key parameter mostly based on 2-8 data points, there is enough evidence to support it being reasonably robust for the tested scenarios.

Two minor comments:

Equation (4): for consistency with Equation (7), shouldnt C_wb in the CBF terms and C_p in the K_1 term really be C_a ?

We changed Equation (7) to be consistent with Equation (4) (Methods, page 20):

$$Q^{S1TC}(t) = v_b C_{wb}(t - t_d) + K_1 \int_{t_d}^{t} C_p(\tau - t_d) e^{-k_2(t-\tau)} H(t - \tau)\, d\tau \qquad (7)$$

Note, the whole blood/plasma notation is commonly used in PET kinetic modeling. Also, we clarified some notation in the Methods (page 19):

> *where $C_{wb}(t)$ and $C_p(t)$ are the whole-blood and plasma arterial input functions, respectively, and the distinction accounts for whole-blood flowing through the blood vessels whereas tracer exchange with the extravascular space occurs in plasma.*

In R.1.11., The correlation illustrated in Supplementary Figure 6 could indicate parameter coupling. In the authors response, they suggested that these correlations may become decoupled in a disease state, but their reference to Figure 5 doesnt necessarily support that, so an additional correlation plot/analysis would be very useful in support of that point.

We have clarified our analysis by plotting CBF vs FDG BBB PS according to brain subregions (grey matter, white matter, cerebellum). This analysis showed that CBF and FDG BBB PS were generally correlated in our healthy cohort even at the subregion level, but the correlation became weaker (and non-significant) for our MASLD cohort in grey matter and cerebellum. This data was added to Supplementary Figure 6:

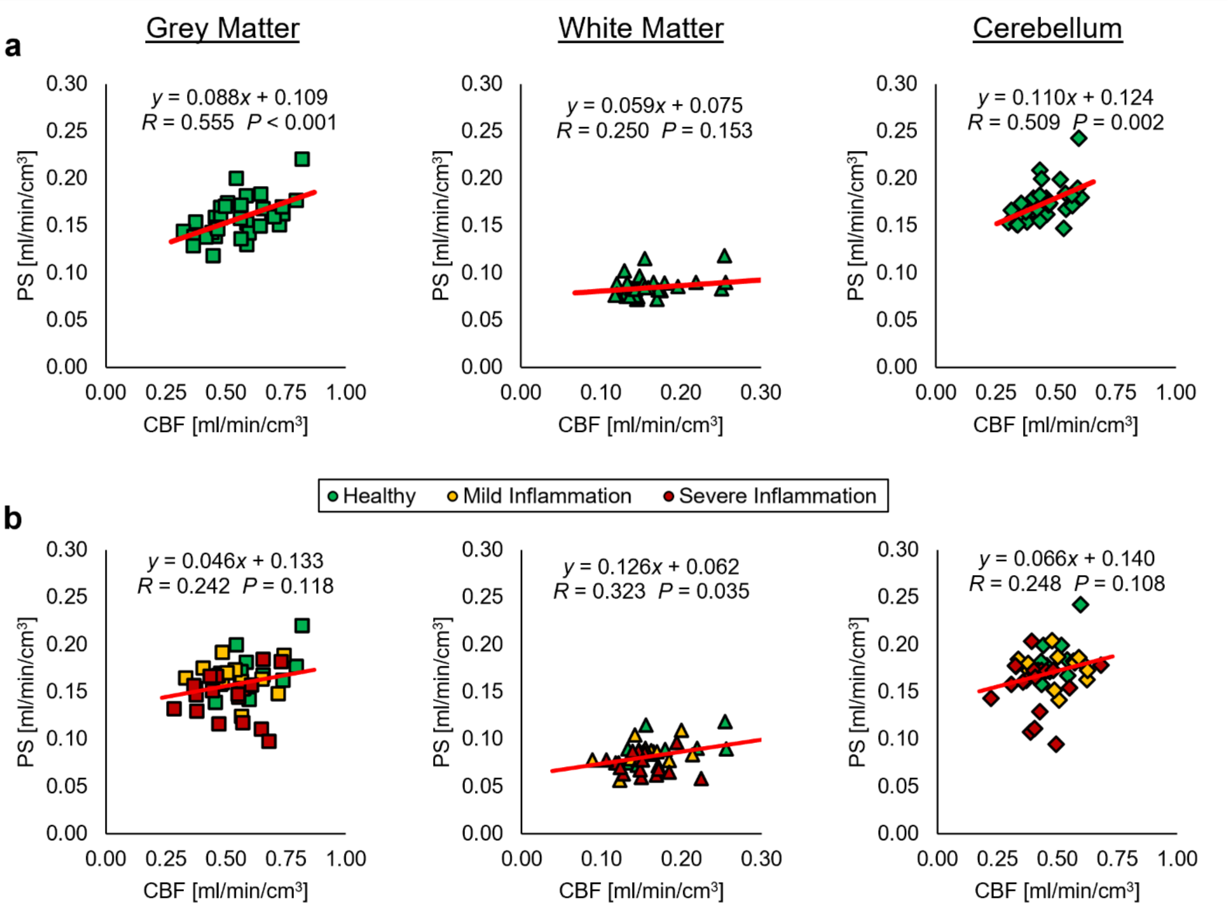

Supplementary Figure 6. Regional cerebral blood flow (CBF) versus the blood-brain barrier (BBB) permeability-surface area (PS) product of ^{18}F-fluorodeoxyglucose (FDG) in (a) 34 healthy subjects and (b) age-matched healthy controls (N=13) and patients with metabolic dysfunction-associated liver disease (MASLD)-related mild liver inflammation (N=13) and severe inflammation (N=17). Data are stratified by grey matter, white matter, and cerebellum.

We additionally commented on this in the Results (Page 10-11):

> *CBF did not significantly differ (P>0.05) between the three groups in any brain regions of interest, suggesting an effect on the BBB but perhaps not CBF in our cohort of MASLD patients with severe liver inflammation. Accordingly, CBF and FDG BBB PS did not strongly correlate in this cohort at the regional level in contrast to our analysis of healthy volunteers (Supplementary Figure 6). Taken together, severe liver inflammation may be a contributing factor to MASLD-related BBB dysregulation, possibly through proinflammatory cytokines circulating in blood and disrupting BBB transport.*[4]

Reviewer #3

The authors substantially revised the manuscript and addressed all my comments (except one, but I accept that this may be part of future work). Additional analysis were performed and added to the manuscript (or supplemental material). I think it is a very interesting approach, very well explored and evaluated. Based on the response to my comments and the other 3 reviewers, ***I have no further major comments and one minor comment***

The authors prefer to report CBF per unit voxel volume (rather than tissue volume) based on the average 5% blood volume fraction they indicate that this is a minor difference. I partly agree in case of grey matter tissue, but

only regions that are not intersected with a larger blood vessel. So, i do think the authors should clarify in the manuscript that K1 (or CBF) is expressed per voxel volume and not per tissue volume (as would be expected when reporting CBF). It is fine to mention that the expected difference is 5% assuming average blood volume fraction of 5%.

This was also discussed in the Methods (Page 20):

> *Of note, the tissue volume fraction, v_t=1-v_b, commonly seen as a scaling factor of the second term of Equation (7), is included as a part of K1. This was chosen to be consistent with the original AATH model[46] and is also commonly used in DCE-MRI studies.[44,98] K1 is therefore expressed per unit voxel volume as opposed to tissue volume. CBF and PS also follow this convention. As the brain has a cerebral blood volume fraction of ≈5%,[19,25] differences in absolute values due to following this convention is negligible.*

For further clarification, we added to the Results (Page 6):

> *Of note, we follow the unit convention used by the original AATH model and in DCE-MRI literature in which CBF, K1, and PS are expressed per unit voxel volume rather than extravascular tissue volume as commonly used in PET. This generally has negligible impact on quantification in the brain due to its small regional blood volume fraction (Supplementary Table 1) but may have greater impact on regions that intersect with larger cerebral vessels.*

Reviewer #4

The concerns of this reviewer have been thoughtfully and thoroughly addressed.

Thank you for reviewing the paper again.
